# Temporal trends and geographical variations in pediatric urinary tract infections: a comprehensive analysis using the global burden of disease study 2021

**DOI:** 10.1186/s41182-025-00829-y

**Published:** 2025-11-24

**Authors:** Yulin Zhang, Shenghan Huang, Yaru Wang, Wei Huang, Xiangxiang Chen, Cuimin Su, Liping Lin, Ruoji Chen

**Affiliations:** Jinjiang Municipal Hospital, Shanghai Sixth People’s Hospital Fujian, Quanzhou, China

**Keywords:** Global burden of disease, Pediatric, Urinary tract infections, Socio-demographic index, Disability-adjusted life years, Geographical variations, Age-standardized incidence

## Abstract

**Background:**

Urinary tract infections (UTIs) are a major public health concern, yet the burden in children remains poorly quantified. This study analyzed global, regional, and national trends in pediatric UTIs from 1990 to 2021.

**Methods:**

Using Global Burden of Disease (GBD) 2021 data, we assessed incidence and disability-adjusted life years (DALYs) for individuals aged ≤ 14 years. Age-standardized incidence (ASIR) and DALY rates (ASDR), along with estimated annual percentage changes (EAPC), were calculated by age, sex, and region.

**Results:**

From 1990 to 2021, the incidence of urinary tract infections and the global burden of associated diseases remained consistently higher among pediatric populations compared to the general population. Globally, there were 50,173,655 UTI cases in 2021, marking a 10% increase from 45,485,831 in 1990. The age-standardized incidence rate per 100,000 population decreased from 2,615.40 in 1990 to 2,493.89 in 2021, with an estimated annual percentage change of −17% (95% confidence interval [CI] −0.33 to −0.01). Additionally, the number of disability-adjusted life years associated with UTIs in pediatric populations decreased by 33%, from 827,127 in 1990 to 554,185 in 2021. The age-standardized disability rate also decreased from 0.53 per 100,000 in 1990 to 0.30 per 100,000 in 2021, with an EAPC of −1.36 (95% CI −1.51 to −1.21). The highest burden was in low-middle SDI regions, especially South Asia and Sub-Saharan Africa.

**Conclusion:**

Despite a modest decline in age-standardized rates, the absolute burden of pediatric UTIs increased from 1990 to 2021. Disparities across SDI regions highlight the need for targeted, age- and region-specific public health strategies to reduce the global impact of pediatric UTIs.

**Supplementary Information:**

The online version contains supplementary material available at 10.1186/s41182-025-00829-y.

## Introduction

Urinary tract infection in children is one of the most common bacterial infections in childhood, affecting approximately 1.7% of boys and 8.4% of girls by the age of 7 years [1]. Urinary tract infections have diverse symptoms and can present as asymptomatic, but they can also lead to short-term complications such as fever, dysuria, and low back pain, and may also lead to long-term kidney damage [2]. According to a survey, the global incidence of UTI was 400 million cases in 2019, a 60.40% increase from 1990 [3]. With the complex impact of rapid advances in medical diagnostic and therapeutic technologies, rapid changes in socio-economic development, and rapid shifts in social factors such as changes in lifestyle and the environment, UTIs have become an important health problem that requires global attention and has placed a significant burden on the global healthcare system [4]. Therefore, the global burden of the disease of UTI warrants an updated assessment to better understand its specific impact on public health.

Urinary tract infections can occur through both hematogenous and epithelial pathways and are primarily associated with bacterial infections, host innate immune responses, and urogenital malformations [5]. The bacterial virulence factor lipopolysaccharide (LPS) binds to toll-like receptor 4 (TLR4) via CD14 on the cell surface, initiating TLR4 signaling. This activation triggers the release of transcription factors that facilitate neutrophil recruitment and cytokine production, crucial for bacterial clearance. Consequently, UTIs are predominantly treated with a range of antibiotics [6]. These underlying mechanisms contribute to the clinical manifestations of UTIs, such as fever, pyuria, and extra-urinary tract infections. Furthermore, involvement of the renal parenchyma in UTIs may provoke an inflammatory response, potentially resulting in permanent renal damage. The long-term sequelae of such damage include hypertension and impaired renal function [7]. The global burden of UTIs is on the rise, and there are notable disparities in the incidence and outcomes of UTIs across different countries. These differences can largely be attributed to variations in the availability and affordability of antibiotics in regions with different economic development levels. Additional factors such as governmental regulation, sanitation, and hygiene also play significant roles in shaping the burden of UTIs [8]. For instance, certain regions of Latin America and Europe report high mortality rates for UTIs across all age groups, whereas sub-Saharan Africa has the lowest mortality rates. The discrepancies may be explained by factors including inadequate data quality due to limited laboratory infrastructure, insufficient government regulation leading to easy access to antibiotics without prescriptions, and the circulation of counterfeit medications [9].

The Global Burden of Disease study, a leading data system in international public health, provides a comprehensive framework for understanding the epidemiological status of various diseases. It serves as a crucial resource, offering insights into disease prevalence, incidence, mortality, and disability-adjusted life years. In contrast to earlier studies of urinary tract infections using GBD data [9], this study incorporates the most recent disease-specific data for the year 2021. Furthermore, it presents a more detailed categorization of UTI incidence, mortality, disease burden, and risk factors, broken down by age, sex, geographic region, and SDI. The analysis emphasizes SDI distributions and temporal trends in disease burden. Additionally, we applied an Autoregressive Integrated Moving Average Model (ARIMA) to forecast future epidemiological trajectories. This updated framework can aid clinicians, epidemiologists, and health policymakers in improving the allocation of healthcare resources and in developing more effective public health strategies.

## Method

### Study population and data source

The Global Burden of Disease (GBD) 2021 dataset provides the most recent estimates for 369 diseases and injuries, 288 causes of death, and 88 risk factors across 204 countries and territories from 1990 to 2021 [10]. In this study, we focused on the pediatric population. We obtained annual statistics on morbidity, mortality, disability-adjusted life years, and their corresponding age-standardized rates (ASRs) for pediatric urinary tract infections, along with 95% uncertainty intervals (UIs), from the Global Health Data Exchange (GHDx) website (https://vizhub.healthdata.org/gbd-results/). Additionally, we utilized the GBD 2021 classification system [11, 12], which categorizes the world into 21 geographic regions based on epidemiological similarity and geographic proximity. This approach provides a more nuanced understanding of geographic variations in disease burden, which can inform the development of targeted public health policies and interventions.

The SDI is a composite indicator introduced in 2015 by the Institute for Health Metrics and Evaluation (IHME) in the United States to assess the development level of countries or regions, emphasizing the interconnections between social development and population health outcomes [13]. Specifically, the SDI is the geometric mean of three components: the total fertility rate (TFR) for the population under 25 years of age, the mean educational attainment of individuals aged 15 years and older, and the lagged distribution of per capita income [14]. In the GBD 2021 dataset, SDI values range from 0 to 1, where 0 represents the lowest income, lowest educational attainment, and highest fertility, while 1 indicates the highest income, highest educational attainment, and lowest fertility [15]. Based on these values, the 204 countries and territories are categorized into five SDI zones: low, medium–low, medium, medium–high, and high [10].

### Ethics statement

For this study, an ethical review was conducted, and an informed consent waiver was granted. The study adhered to the Guidelines for Accurate and Transparent Reporting of Health Estimates (GATHER).

### Statistical analysis

Previous studies have extensively outlined the protocols and methods used in Global Burden of Disease (GBD) studies [13]. The primary indicators of the burden of urinary tract infections in children include the number of cases, disability-adjusted life years, and their corresponding age-standardized rates (ASRs). These indicators help examine the distribution of the burden of urinary tract infections across different age cohorts and genders. In this study, the age-standardized incidence rate (ASIR) and age-standardized disability-adjusted life years (ASDR) were calculated using the following formula:

$${\text{ASR}}\, = \,\frac{{\mathop \sum \nolimits_{{\text{i = 1}}}^{{\text{A}}} {\upalpha }_{{\text{i}}} {\text{w}}_{{\text{i}}} }}{{\mathop \sum \nolimits_{{\text{i = 1}}}^{{\text{A}}} {\text{w}}_{{\text{i}}} }}$$

(${\alpha}_i$: age-standardized rate for the i-th age group; W: number of individuals in the corresponding age group in the standard population; A: total number of age groups).

In epidemiological studies, the estimated annual percentage change (EAPC) is a commonly used metric for assessing changes in disease age-standardized rates (ASR) over time [16]. To evaluate trends in the burden of urinary tract infections in children from 1990 to 2021, we calculated the EAPC for the age-standardized incidence rate (ASIR) and age-standardized disability-adjusted life years. To ensure the validity of our model, we verified the linear regression assumption through a multi-step approach. First, we confirmed the linear trend of the natural log-transformed ASR over calendar years using scatterplots and residual analysis. Second, we tested the independence of the residuals using the Durbin-Watson statistic and examined their normality through residual histograms and Q-Q plots. Finally, we further validated the model by assessing the variance of residuals using residual-fitted-value scatterplots and a chi-square test [17]. Using the natural logarithmic fitted linear regression model, we determined the EAPC and its 95% confidence interval (CI)

$${\text{y}}\, = \,\alpha \, + \,\beta {\text{x}}\, + \,\varepsilon$$

In this model, y represents ln(ASR), while x denotes the corresponding calendar year. The coefficient β represents the geometric mean ratio, and ε is the error term. The estimated EAPC is 100 × (e^β−1) with a 95% confidence interval (CI). Positive EAPC values and their corresponding 95% CIs indicate an upward trend in ASR, while negative EAPC values and their corresponding 95% CIs indicate a downward trend. If the 95% CI includes 0, the corresponding EAPC for ASR is considered not statistically significant.

In addition, to examine the relationship between the age-standardized rates (ASRs) of childhood urinary tract infections and the Socio-Demographic Index, we applied LOESS regression analysis. LOESS (locally estimated scatterplot smoothing) is a nonparametric regression method used to fit smooth curves to scatterplots. In this method, data points within the neighborhood of each data point are assigned different weights, with points closer to the target receiving higher weights. This approach allows for modeling the potentially complex relationship between SDI and the burden of urinary tract infections in children [18]. All statistical analyses were performed using R software (version 4.2.1).

## Results

### Global burden and trend of pediatric urinary tract infections

From 1990 to 2021, the incidence and disease burden associated with paediatric urinary tract infections consistently remained higher in the paediatric population compared to the general population. Globally, the number of paediatric UTI cases showed a gradual yet persistent increase over this period. Specifically, the total number of cases rose from 45,485,831 in 1990 to 50,173,655 in 2021, reflecting a 10% increase. However, when adjusted for population changes, the ASIR per 100,000 population exhibited a decline, falling from 2,615.40 in 1990 to 2,493.89 in 2021. This corresponds to an EAPC of −17% (95% CI −0.33 to −0.01) (Table 1). In terms of mortality, deaths attributable to pediatric UTIs decreased by 34%, from 9,206 in 1990 to 6,065 in 2021. Similarly, ASDR per 100,000 population declined from 0.53 to 0.30 during the same interval. This represents an EAPC of −1.36% (95% CI −1.51 to −1.21) (Table 2).

**Table 1:** The incidence cases and ASIR of pediatric urinary tract infection in 1990 and 2021, with temporal trends from 1990 to 2021

	Incidence cases (95% CI)		Case change	ASIR (95% CI)		1990–2021 EAPCs(95%CI)
	1990	2021		1990	2021	
Global	45,485,831 (40,328,913–51,549,924)	50,173,655 (44,639,939–56,681,228)	0.10 (0.04–0.14)	2615.40 (2318.88–2964.09)	2493.89 (2218.84–2817.35)	−0.17 (−0.33 to −0.01)
SDI						
High SDI	6,367,488 (5,633,217–7,238,628)	5,148,699 (4,526,943–5,885,618)	−0.19 (−0.21 to −0.17)	3426.92 (3031.74–3895.76)	2984.12 (2623.76–3411.23)	−0.71 (−0.84 to −0.59)
High-middle SDI	7,497,134 (6,648,345–8,397,305)	6,408,094 (5,701,694–7,198,173)	−0.15 (−0.18 to −0.12)	2739.95 (2429.75–3068.93)	2775.38 (2469.44–3117.57)	0.29 (−0.00 to 0.58)
Middle SDI	11,916,961 (10,426,792–13,549,131)	13,518,144 (11,845,739–15,281,145)	0.13 (0.08–0.17)	2064.57 (1806.40–2347.33)	2384.74 (2089.71–2695.75)	0.45 (0.32–0.59)
Low-middle SDI	14,917,132 (12,952,426–17,549,846)	17,309,562 (15,163,347–19,737,405)	0.16 (0.05–0.22)	3159.66 (2743.51–3717.30)	2985.22 (2615.09–3403.93)	−0.20 (−0.39 to −0.00)
Low SDI	4,742,658 (4,102,302–5,653,506)	7,750,855 (6,696,072–8,790,013)	0.63 (0.47–0.76)	2071.81 (1792.08–2469.71)	1684.14 (1454.95–1909.94)	−0.53 (−0.88 to −0.18)
Central Europe, eastern Europe, and central Asia						
Central Asia	893,714 (781,358–1,022,909)	1,000,661 (880,705–1,146,626)	0.12 (0.07–0.17)	3576.12 (3126.53–4093.08)	3615.65 (3182.22–4143.07)	0.07 (0.03–0.11)
Central Europe	1,397,651 (1,245,921–1,565,831)	945,128 (833,927–1,051,923)	−0.32 (−0.35 to −0.29)	4740.43 (4225.81–5310.85)	5339.34 (4711.13–5942.66)	−0.75 (−1.09 to −0.41)
Eastern Europe	4,547,772 (4,029,543–5,075,722)	3,701,228 (3,299,980–4,144,617)	−0.19 (−0.23 to −0.14)	8837.15 (7830.14–9863.05)	10442.46 (9310.40–11693.41)	0.72 (0.31–1.13)
High income region						
High-income Asia Pacific	2,226,429 (1,967,097–2,525,417)	1,359,836 (1,202,615–1,553,545)	−0.39 (−0.42 to −0.36)	6325.17 (5588.42–7174.57)	6063.78 (5362.70–6927.57)	0.01 (−0.08 to 0.10)
High-income North America	1,924,581 (1,679,769–2,225,204)	1,845,683 (1,616,438–2,113,576)	−0.04 (−0.07 to −0.01)	3120.39 (2723.46–3607.80)	2812.73 (2463.37–3220.99)	−1.02 (−1.44 to −0.59)
Western Europe	1,341,260 (1,144,212–1,581,272)	1,241,827 (1,064,526–1,453,932)	−0.07 (−0.11 to −0.03)	1888.61 (1611.15–2226.57)	1823.04 (1562.76–2134.42)	0.07 (−0.03 to 0.18)
Australasia	174,826 (148,844–206,182)	224,050 (191,309–264,668)	0.28 (0.14–0.43)	3812.20 (3245.65–4495.95)	3909.34 (3338.07–4618.08)	0.02 (−0.01 to 0.05)
Latin America and Caribbean						
Andean Latin America	637,733 (538,412–758,398)	874,682 (731,445–1,077,794)	0.37 (0.23–0.59)	4293.91 (3625.17–5106.36)	4833.89 (4042.30–5956.38)	0.59 (0.51–0.68)
Caribbean	496,200 (424,121–578,152)	466,808 (394,287–558,120)	−0.06 (−0.12 to 0.01)	4347.90 (3716.31–5065.99)	4057.40 (3427.07–4851.07)	−0.23 (−0.27 to −0.20)
Southern Latin America	337,261 (286,235–395,717)	336,381 (282,128–409,260)	−0.00 (−0.10 to 0.10)	2259.49 (1917.64–2651.12)	2320.59 (1946.31–2823.36)	−0.02 (−0.16 to 0.11)
Tropical Latin America	2,021,894 (1,759,763–2,346,940)	2,537,306 (2,215,505–2,933,404)	0.25 (0.20–0.31)	3771.21 (3282.29–4377.48)	5055.10 (4413.97–5844.24)	1.12 (1.01–1.23)
Central Latin America	4,158,818 (3,577,915–4,811,611)	5,308,918 (4,597,359–6,157,562)	0.28 (0.23–0.34)	6459.68 (5557.39–7473.63)	8362.48 (7241.65–9699.25)	1.02 (0.74–1.29)
North Africa and Middle East						
North Africa and Middle East	3,775,706 (3,227,330–4,414,644)	4,925,490 (4,154,540–5,856,810)	0.30 (0.25–0.36)	2687.61 (2297.27–3142.42)	2686.80 (2266.25–3194.82)	−0.03 (−0.06 to 0.01)
South Asia						
South Asia	17,625,702 (14,974,064–21,381,931)	18,914,089 (16,490,026–21,753,525)	0.07 (−0.05 to 0.13)	4067.20 (3455.33–4933.97)	3730.43 (3252.33–4290.45)	−0.36 (−0.59 to −0.12)
Southeast Asia, east Asia, and Oceania						
East Asia	904,571 (788,071–1,078,318)	498,184 (429,582–570,270)	−0.45 (−0.51 to −0.41)	274.25 (238.93–326.93)	186.34 (160.68–213.30)	−1.60 (−1.97 to −1.23)
Oceania	8,081 (6,947–9,411)	14,740 (12,209–17,541)	0.82 (0.67–0.97)	301.55 (259.26–351.20)	290.11 (240.30–345.24)	−0.09 (−0.11 to −0.07)
Southeast Asia	744,809 (635,343–879,903)	788,382 (669,954–930,141)	0.06 (0.02–0.11)	436.21 (372.10–515.32)	456.63 (388.03–538.73)	−0.16 (−0.30 to −0.01)
Sub-Saharan Africa						
Central Sub-Saharan Africa	228,912 (194,682–272,159)	534,166 (455,986–646,763)	1.33 (1.18–1.51)	904.84 (769.54–1075.79)	910.28 (777.05–1102.16)	−0.02 (−0.03 to −0.00)
Eastern Sub-Saharan Africa	627,562 (543,612–730,760)	1,397,306 (1,185,121–1,652,309)	1.23 (1.15–1.33)	692.90 (600.21–806.84)	783.11 (664.19–926.02)	0.12 (0.05–0.19)
Southern Sub-Saharan Africa	357,131 (309,487–415,747)	413,251 (355,759–487,894)	0.16 (0.11–0.21)	1726.16 (1495.88–2009.48)	1717.17 (1478.27–2027.33)	−0.04 (−0.09 to 0.00)
Western Sub-Saharan Africa	1,055,210 (912,279–1,200,578)	2,845,529 (2,488,332–3,232,349)	1.70 (1.56–1.81)	1200.74 (1038.10–1366.16)	1324.96 (1158.64–1505.07)	−0.23 (−0.46 to −0.01)

**Table 2:** The death cases and ASDR of pediatric urinary tract infection in 1990 and 2021, with TEMPORAL TRENDS from 1990 to 2021

	Deaths cases (95% CI)		Case change	ASDR (95% CI)		1990–2021 EAPCs (95%CI)
	1990	2021		1990	2021	
Global	9,206 (6,746–11,325)	6,065 (4,661–7,465)	−0.34 (−0.48 to −0.07)	0.53 (0.39–0.65)	0.30 (0.23–0.37)	−1.36 (−1.51 to −1.21)
SDI						
High SDI	99 (92–108)	54 (49–59)	−0.45 (−0.51 to −0.38)	0.05 (0.05–0.06)	0.03 (0.03–0.03)	−1.23 (−1.42 to −1.03)
High-middle SDI	303 (255–340)	103 (92–116)	−0.66 (−0.71 to −0.58)	0.11 (0.09–0.12)	0.04 (0.04–0.05)	−2.56 (−2.73 to −2.40)
Middle SDI	1,661 (1,391–1,893)	813 (690–939)	−0.51 (−0.59 to −0.39)	0.29 (0.24–0.33)	0.14 (0.12–0.17)	−1.43 (−1.67 to −1.19)
Low-middle SDI	4,403 (3,135–5,507)	2,819 (2,081–3,648)	−0.36 (−0.52 to −0.02)	0.93 (0.66–1.17)	0.49 (0.36–0.63)	−1.68 (−1.81 to −1.55)
Low SDI	2,735 (1,724–3,618)	2,271 (1,702–2,834)	−0.17 (−0.36 to 0.27)	1.19 (0.75–1.58)	0.49 (0.37–0.62)	−2.68 (−2.78 to −2.57)
Central Europe, eastern Europe, and central Asia						
Central Asia	72 (64–81)	69 (58–85)	−0.04 (−0.21 to 0.18)	0.29 (0.26–0.33)	0.25 (0.21–0.31)	0.29 (−0.18 to 0.76)
Central Europe	27 (26–29)	6 (5–7)	−0.77 (−0.80 to −0.72)	0.09 (0.09–0.10)	0.04 (0.03–0.04)	−2.05 (−3.40 to −0.69)
Eastern Europe	66 (63–70)	21 (20–23)	−0.67 (−0.69 to −0.65)	0.13 (0.12–0.14)	0.06 (0.06–0.07)	−2.38 (−2.92 to −1.84)
High income region						
High-income Asia Pacific	8 (8–10)	4 (4–5)	−0.44 (−0.55 to −0.32)	0.03 (0.02–0.03)	0.02 (0.02–0.03)	0.51 (0.14–0.89)
High-income North America	34 (33–35)	23 (21–25)	−0.31 (−0.36 to −0.24)	0.06 (0.05–0.06)	0.04 (0.03–0.04)	−0.91 (−1.22 to −0.59)
Western Europe	15 (14–15)	15 (13–17)	0.02 (−0.09 to 0.14)	0.02 (0.02–0.02)	0.02 (0.02–0.03)	0.89 (0.20–1.57)
Australasia	3 (3–3)	2 (1–2)	−0.40 (−0.49 to −0.27)	0.08 (0.07–0.08)	0.04 (0.03–0.04)	−0.90 (−1.28 to −0.52)
Latin America and Caribbean						
Andean Latin America	102 (85–123)	50 (35–66)	−0.51 (−0.67 to −0.31)	0.69 (0.58–0.83)	0.28 (0.19–0.37)	−2.43 (−2.70 to −2.15)
Caribbean	24 (13–32)	26 (14–41)	0.11 (−0.30 to 0.66)	0.21 (0.12–0.28)	0.23 (0.13–0.36)	0.94 (0.77–1.10)
Southern Latin America	7 (7–8)	14 (12–17)	0.90 (0.61–1.28)	0.05 (0.05–0.06)	0.10 (0.09–0.12)	2.88 (2.20–3.56)
Tropical Latin America	305 (270–339)	189 (150–228)	−0.38 (−0.51 to −0.22)	0.57 (0.50–0.63)	0.38 (0.30–0.46)	−0.20 (−0.57 to 0.17)
Central Latin America	250 (231–273)	148 (115–191)	−0.41 (−0.54 to −0.23)	0.39 (0.36–0.43)	0.23 (0.18–0.30)	−0.73 (−1.16 to −0.31)
North Africa and Middle East						
North Africa and Middle East	367 (258–457)	157 (112–193)	−0.57 (−0.67 to −0.41)	0.26 (0.18–0.33)	0.09 (0.06–0.11)	−2.93 (−3.10 to −2.75)
South Asia						
South Asia	4,900 (3,506–6,214)	3,057 (2,214–4,133)	−0.38 (−0.54 to −0.03)	1.13 (0.81–1.43)	0.60 (0.44–0.82)	−1.67 (−1.75 to −1.58)
Southeast Asia, east Asia, and Oceania						
East Asia	388 (239–484)	44 (36–59)	−0.88 (−0.91 to −0.78)	0.12 (0.07–0.15)	0.02 (0.01–0.02)	−6.62 (−6.89 to −6.34)
Oceania	4 (2–7)	7 (4–10)	0.58 (0.07–1.41)	0.17 (0.09–0.26)	0.14 (0.09–0.21)	−0.42 (−0.61 to −0.23)
Southeast Asia	419 (268–523)	250 (198–297)	−0.40 (−0.53 to −0.10)	0.25 (0.16–0.31)	0.15 (0.11–0.17)	−1.37 (−1.49 to −1.25)
Sub-Saharan Africa						
Central Sub-Saharan Africa	96 (43–140)	72 (49–101)	−0.24 (−0.46 to 0.48)	0.38 (0.17–0.55)	0.12 (0.08–0.17)	−3.31 (−3.57 to −3.06)
Eastern Sub-Saharan Africa	1,214 (695–1,748)	881 (625–1,179)	−0.27 (−0.47 to 0.45)	1.34 (0.77–1.93)	0.49 (0.35–0.66)	−3.01 (−3.14 to −2.87)
Southern Sub-Saharan Africa	9 (5–12)	9 (6–11)	−0.01 (−0.32 to 0.42)	0.04 (0.03–0.06)	0.04 (0.03–0.05)	−0.21 (−0.44 to 0.03)
Western Sub-Saharan Africa	886 (465–1,171)	1,010 (630–1,345)	0.14 (−0.11 to 0.55)	1.01 (0.53–1.33)	0.47 (0.29–0.63)	−2.14 (−2.28 to −2.01)

**Fig. 1:**
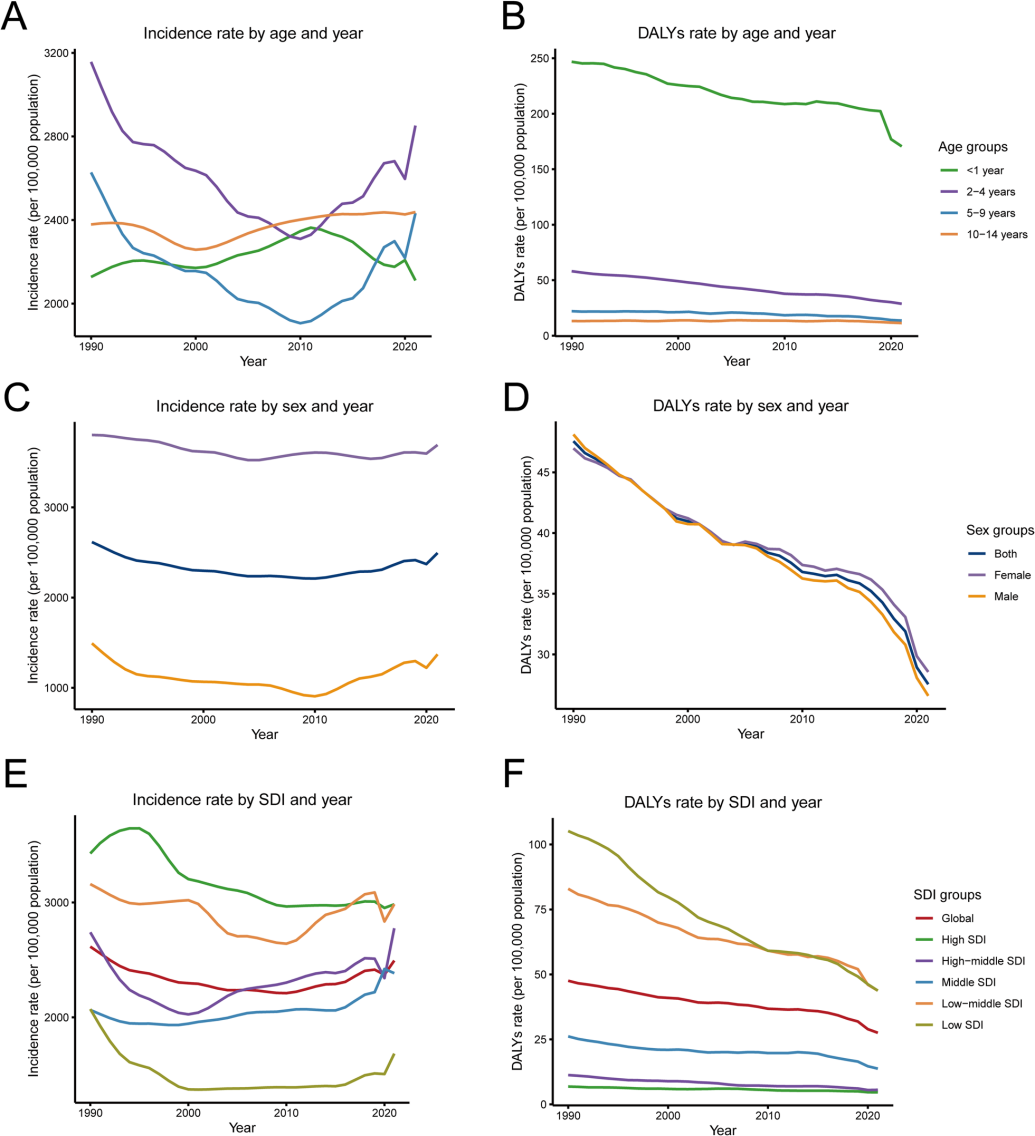
Age-standardized incidence rate and age-standardized DALYs rate per 100,000 population of pediatric urinary tract infections grouped by age, sex, and SDI quintiles from 1990 to 2023. Incidence rate (**A**) and DALYs rate (**B**) by age group and year. Incidence rate (**C**) and DALYs rate (**D**) by sex group and year.Incidence rate (**E**) and DALYs rate (**F**) by SDI group and year. *DALYs* disability-adjusted life-years, *SDI* socio-demographic index

**Fig. 2:**
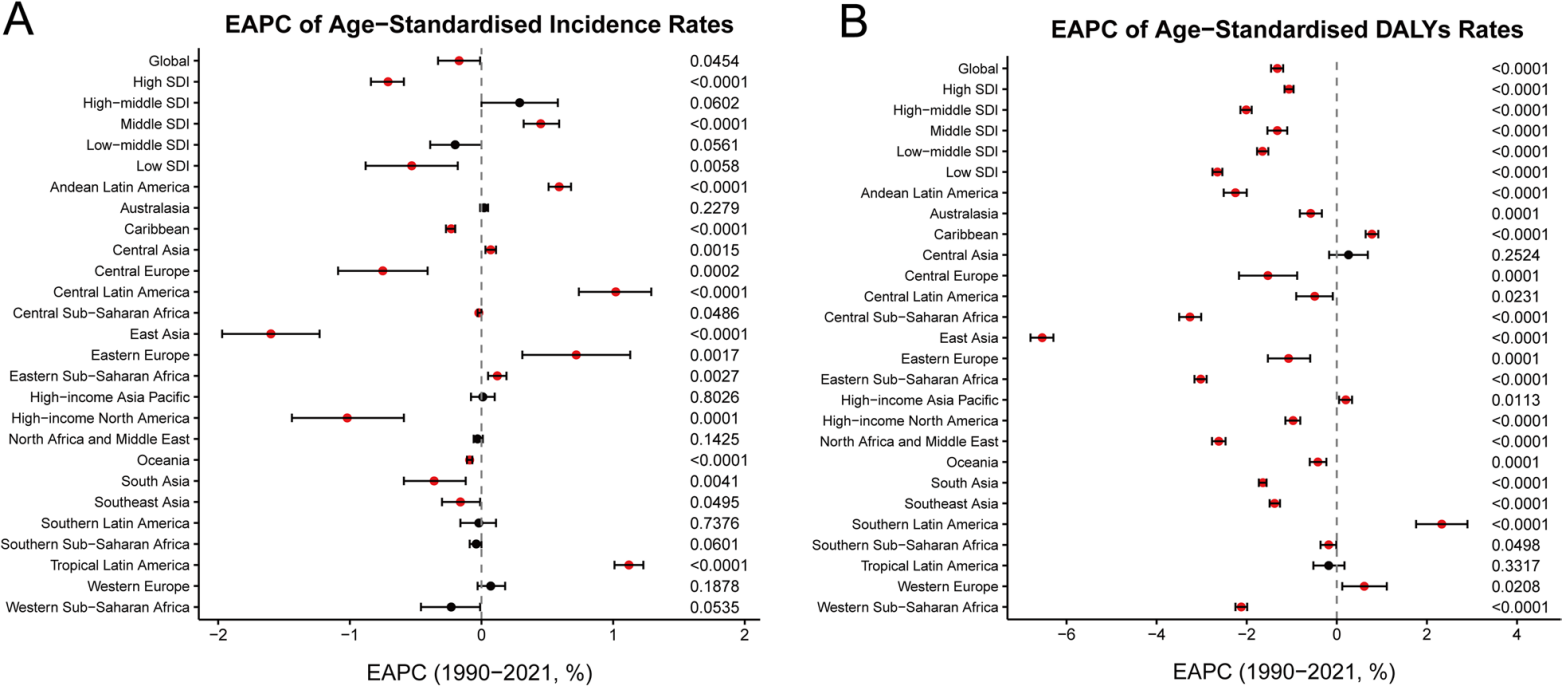
Estimated annual percentage changes of age-standardised incidence rates (**A**) and age-standardised DALYs rates (**B**) for pediatric urinary tract infections from 1990 to 2021, by SDI region and GBD region. The circle represents the point estimate of EAPC of the age-standardised incidence rate or age-standardised DALYs rate, and the error bar represents the corresponding 95% confidence intervals. *EAPC* estimated annual percentage change, *SDI* socio-demographic index, *DALYs* disability-adjusted life-years

Between 1990 and 2021, the epidemiological trend of paediatric urinary tract infections exhibited a non-linear pattern, characterized by an initial overall decline followed by a subsequent increase. Analysis by age subgroup revealed that the highest incidence of UTIs was consistently observed in children aged 2–4 years. A similar temporal pattern in both incidence and disease burden was observed for the adjacent age group of 5–9 year-olds (Fig. 1A). When assessing the disease burden through DALYs, the distribution across age groups differed from that of incidence. The greatest DALYs burden was concentrated among children under 1 year of age, with the 2–4 year-old group representing the second highest burden. In contrast, the lowest burden of DALYs was found in the oldest paediatric subgroup, children aged 10–14 years (Fig. 2B).

### Pediatric urinary tract infections among different SDI regions

Analysis by SDI regions revealed pronounced disparities in the trends of paediatric urinary tract infection incidence from 1990 to 2021. The most substantial increase in case numbers occurred in low SDI regions, which saw a rise of 63%. This was followed by more moderate increases in low-middle (16%) and middle (13%) SDI regions. In contrast, high SDI regions experienced the most significant decrease in incidence (19%), trailed by high-middle SDI regions. By 2021, the low-middle SDI region not only accounted for the highest absolute number of UTI cases (17,309,562) but also exhibited the highest ASIR at 2,985.22 per 100,000 population (Table 1). When assessing the temporal trend in ASIR, all SDI regions except two showed a decline over the 31 years. The high SDI region recorded the most pronounced decrease, with an EAPC of −71% (95% CI −0.84 to −0.59). Notably, the high-middle and middle SDI regions were exceptions to this overall downward trend, demonstrating significant increases in ASIR with EAPCs of 29% (95% CI 0.00–0.58) and 45% (95% CI 0.32–0.59), respectively (Table 1, Fig. 2A).

Between 1990 and 2021, all SDI regions witnessed a reduction in DALYs attributable to paediatric urinary tract infections. The most substantial decline in the number of DALYs was observed in the high-middle SDI region, which decreased by 58% (Table 3). By 2021, the burden of disease was disproportionately concentrated in the low-middle SDI region. This region not only recorded the highest absolute number of DALYs (253,737) but also the highest ASDR at 0.49 per 100,000 population (Tables 2 and 3). Over the entire study period, ASDR demonstrated a consistent downward trend across all SDI regions. Notably, the low SDI region achieved the most pronounced decline in ASDR, with an EAPC of −2.68 (95% CI −2.78 to −2.57) (Table 2 and Fig. 2B).

**Table 3:** The DALYs and age-standardized DALY rate of pediatric urinary tract infection in 1990 and 2021, with temporal trends from 1990 to 2021

	DALYs (95% CI)		Case change	Age-standardized DALY rate (95% CI)		1990–2021 EAPCs (95%CI)
	1990	2021		1990	2021	
Global	827,127 (612,187–1,014,914)	554,185 (434,172–676,905)	−0.33 (−0.47 to −0.07)	47.56 (35.20–58.36)	27.55 (21.58–33.65)	−1.32 (−1.46 to −1.19)
SDI						
High SDI	12,720 (11,015–14,912)	7,962 (6,628–9,774)	−0.37 (−0.43 to −0.32)	6.85 (5.93–8.03)	4.61 (3.84–5.66)	−1.06 (−1.16 to −0.96)
High-middle SDI	30,848 (26,233–35,043)	12,896 (10,929–15,153)	−0.58 (−0.64 to −0.50)	11.27 (9.59–12.81)	5.59 (4.73–6.56)	−2.01 (−2.14 to −1.89)
Middle SDI	150,966 (128,206–172,191)	77,918 (66,527–89,765)	−0.48 (−0.56 to −0.37)	26.15 (22.21–29.83)	13.75 (11.74–15.84)	−1.32 (−1.54 to −1.10)
Low-middle SDI	391,554 (281,528–489,103)	253,737 (188,554–326,994)	−0.35 (−0.52 to −0.01)	82.94 (59.63–103.60)	43.76 (32.52–56.39)	−1.65 (−1.77 to −1.52)
Low SDI	240,724 (153,092–318,171)	201,419 (151,059–250,194)	−0.16 (−0.36 to 0.27)	105.16 (66.88–138.99)	43.77 (32.82–54.36)	−2.65 (−2.76 to −2.54)
Central Europe, eastern Europe, and central Asia						
Central Asia	6,709 (5,996–7,619)	6,447 (5,486–7,848)	−0.04 (−0.19 to 0.17)	26.85 (23.99–30.49)	23.30 (19.83–28.36)	0.26 (−0.17 to 0.69)
Central Europe	3,291 (2,913–3,815)	1,175 (920–1,533)	−0.64 (−0.69 to −0.59)	11.17 (9.88–12.94)	6.64 (5.20–8.66)	−1.53 (−2.17 to −0.88)
Eastern Europe	8,593 (7,391–10,165)	4,273 (3,304–5,524)	−0.50 (−0.56 to −0.45)	16.70 (14.36–19.75)	12.06 (9.32–15.59)	−1.07 (−1.53 to −0.59)
High income region						
High-income Asia Pacific	2,238 (1,635–3,000)	1,328 (967–1,801)	−0.41 (−0.47 to −0.36)	6.36 (4.65–8.53)	5.92 (4.31–8.03)	0.20 (0.05–0.34)
High-income North America	4,177 (3,704—4,802)	3,209 (2,720–3,846)	−0.23 (−0.29 to −0.18)	6.77 (6.01–7.79)	4.89 (4.15–5.86)	−0.97 (−1.14 to −0.81)
Western Europe	2,104 (1,796–2,572)	2,069 (1,742–2,526)	−0.02 (−0.10 to 0.08)	2.96 (2.53–3.62)	3.04 (2.56–3.71)	0.61 (0.12–1.11)
Australasia	414 (366–482)	322 (256–406)	−0.22 (−0.35 to −0.08)	9.04 (8.00–10.52)	5.63 (4.48–7.09)	−0.58 (−0.82 to −0.33)
Latin America and Caribbean						
Andean Latin America	9,403 (7,951–11,317)	4,895 (3,605–6,312)	−0.48 (−0.64 to −0.28)	63.32 (53.54–76.20)	27.05 (19.93–34.88)	−2.25 (−2.51 to −2.00)
Caribbean	2,456 (1,511–3,201)	2,658 (1,586–3,890)	0.08 (−0.28 to 0.53)	21.52 (13.24–28.06)	23.11 (13.79–33.81)	0.78 (0.64–0.92)
Southern Latin America	893 (793–1,033)	1,481 (1,270–1,727)	0.66 (0.39–0.93)	5.99 (5.32–6.92)	10.22 (8.77–11.92)	2.33 (1.76–2.90)
Tropical Latin America	28,281 (25,208–31,354)	18,041 (14,797–21,518)	−0.36 (−0.49 to −0.20)	52.75 (47.02–58.48)	35.95 (29.48–42.87)	−0.18 (−0.52 to 0.17)
Central Latin America	24,564 (22,442–27,349)	16,211 (12,904–20,223)	−0.34 (−0.47 to −0.18)	38.16 (34.86–42.48)	25.54 (20.33–31.86)	−0.49 (−0.90 to −0.09)
North Africa and Middle East						
North Africa and Middle East	34,564 (24,603–42,789)	16,723 (12,747–20,449)	−0.52 (−0.62 to −0.35)	24.60 (17.51–30.46)	9.12 (6.95–11.15)	−2.62 (−2.77 to −2.47)
South Asia						
South Asia	435,394 (315,104–551,302)	274,673 (200,654–370,094)	−0.37 (−0.54 to −0.03)	100.47 (72.71–127.22)	54.17 (39.58–72.99)	−1.64 (−1.73 to −1.56)
Southeast Asia, east Asia, and Oceania						
East Asia	33,878 (20,935–42,107)	4,046 (3,320–5,284)	−0.88 (−0.91 to −0.77)	10.27 (6.35–12.77)	1.51 (1.24–1.98)	−6.54 (−6.80 to −6.29)
Oceania	412 (224–635)	654 (396–971)	0.59 (0.08–1.40)	15.40 (8.37–23.73)	12.88 (7.81–19.13)	−0.42 (−0.60 to −0.23)
Southeast Asia	35,962 (22,955–44,743)	21,506 (17,018–25,496)	−0.40 (−0.53 to −0.10)	21.06 (13.44–26.20)	12.46 (9.86–14.77)	−1.38 (−1.49 to −1.26)
Sub-Saharan Africa						
Central Sub-Saharan Africa	8,573 (3,988–12,482)	6,628 (4,626–9,134)	−0.23 (−0.44 to 0.53)	33.89 (15.77–49.34)	11.30 (7.88–15.57)	−3.26 (−3.50 to −3.01)
Eastern Sub-Saharan Africa	105,794 (60,678–152,527)	76,461 (54,335–102,250)	−0.28 (−0.48 to 0.44)	116.81 (67.00–168.41)	42.85 (30.45–57.31)	−3.02 (−3.16 to −2.89)
Southern Sub-Saharan Africa	1,016 (677–1,352)	1,024 (783–1,287)	0.01 (−0.26 to 0.33)	4.91 (3.27–6.54)	4.26 (3.26–5.35)	−0.18 (−0.36 to −0.01)
Western Sub-Saharan Africa	78,401 (41,671–103,329)	90,351 (57,399–119,842)	0.15 (−0.09 to 0.57)	89.21 (47.42–117.58)	42.07 (26.73–55.80)	−2.12 (−2.25 to −1.99)

### Pediatric urinary tract infections among different GBD regions

According to GBD 2021, urinary tract infection incidence cases and urinary tract infection-associated DALYs were recorded in all 21 GBD regions worldwide.

**Fig. 3:**
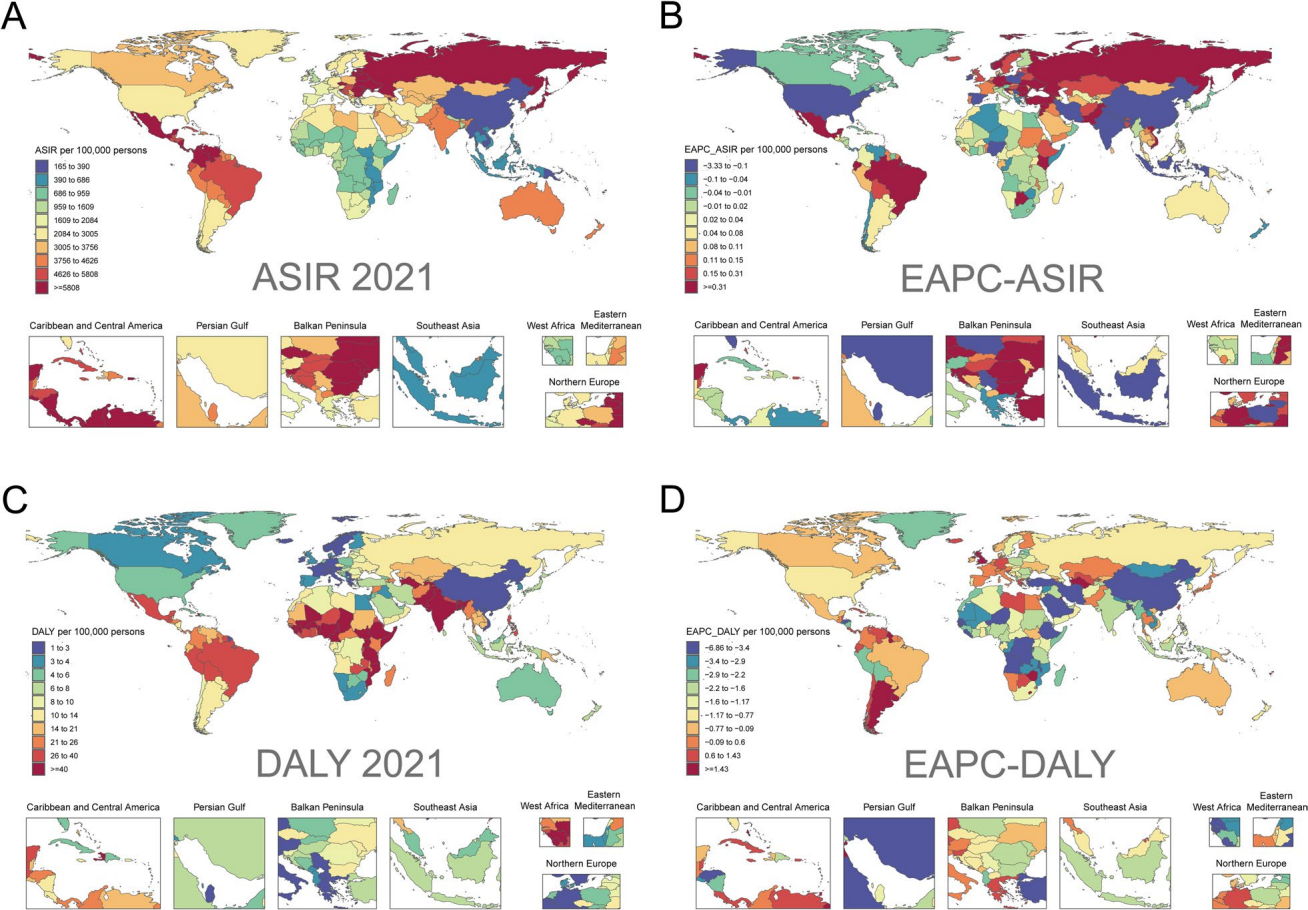
ASIR and DALYs rate of pediatric urinary tract infections in 204 countries or territories worldwide from 1990 to 2021, along with their corresponding EAPCs. ASIR in 2021 (**A**) and DALYs rate in 2021 (**C**). EAPCs of ASIR from 1990 to 2021 (**B**) and EAPCs of DALYs rates from 1990 to 2021 (**D**). *ASIR* Age-standardized incidence rates, *EAPC* estimated annual percentage change, *DALYs* disability-adjusted life-years

The incidence of urinary tract infections was highest in South Asia, with 18,914,089 cases in 2021 (Table 1). From 1990 to 2021, the highest increase in the incidence of urinary tract infections was in Western Sub-Saharan Africa with an increase of 170%, followed by Central Sub-Saharan Africa. Eastern Europe consistently has the highest burden of UTI incidence with an ASIR of 10442.46 cases per 100,000 people in 2021, followed by Central Latin America (ASIR of 8362.48 cases per 100,000 people), High-income Asia Pacific (ASIR of 6,063.78 cases per 100,000 population) (Table 1 and Fig. 3A). From 1990 to 2021, nine regions showed an increasing trend in ASIR, with Tropical Latin America having the highest increasing trend in ASIR (EAPC = 1.12, 95% CI 1.01–1.23). Twelve regions showed a decreasing trend in ASIR, with East Asia (EAPC =−1.60, 95% CI −1.97 to −1.23) showing the highest decreasing trend (Table 1 and Fig. 3B).

Among all GBD regions, South Asia consistently had the highest burden of DALYs due to urinary tract infections highest number of DALYs (274,673 in 2021) and ASDR (0.60 per 100,000 people in 2021) (Tables 2, 3, and Fig. 3C). From 1990 to 2021, Southern Latin America had the highest increase in the number of DALYs associated with urinary tract infections, with an increase of 66%, followed by Oceania (59%) and Western Sub-Saharan Africa (15%) (Table 3). Five GBD regions showed decreasing trends in ASDR. Southern Latin America had the largest increasing trend in ASDR (EAPC = 2.88, 95% CI 2.20–3.56), and 16 GBD regions had a decreasing trend in ASDR, with East Asia having the largest decrease (EAPC =−6.62, 95% CI, −6.89 to −6.34) followed by Central Sub-Saharan Africa (EAPC =−3.31, 95% CI −3.57 to −3.06) (Table 2 and Fig. 3D).

### Pediatric urinary tract infections among different countries and territories

**Fig. 4:**
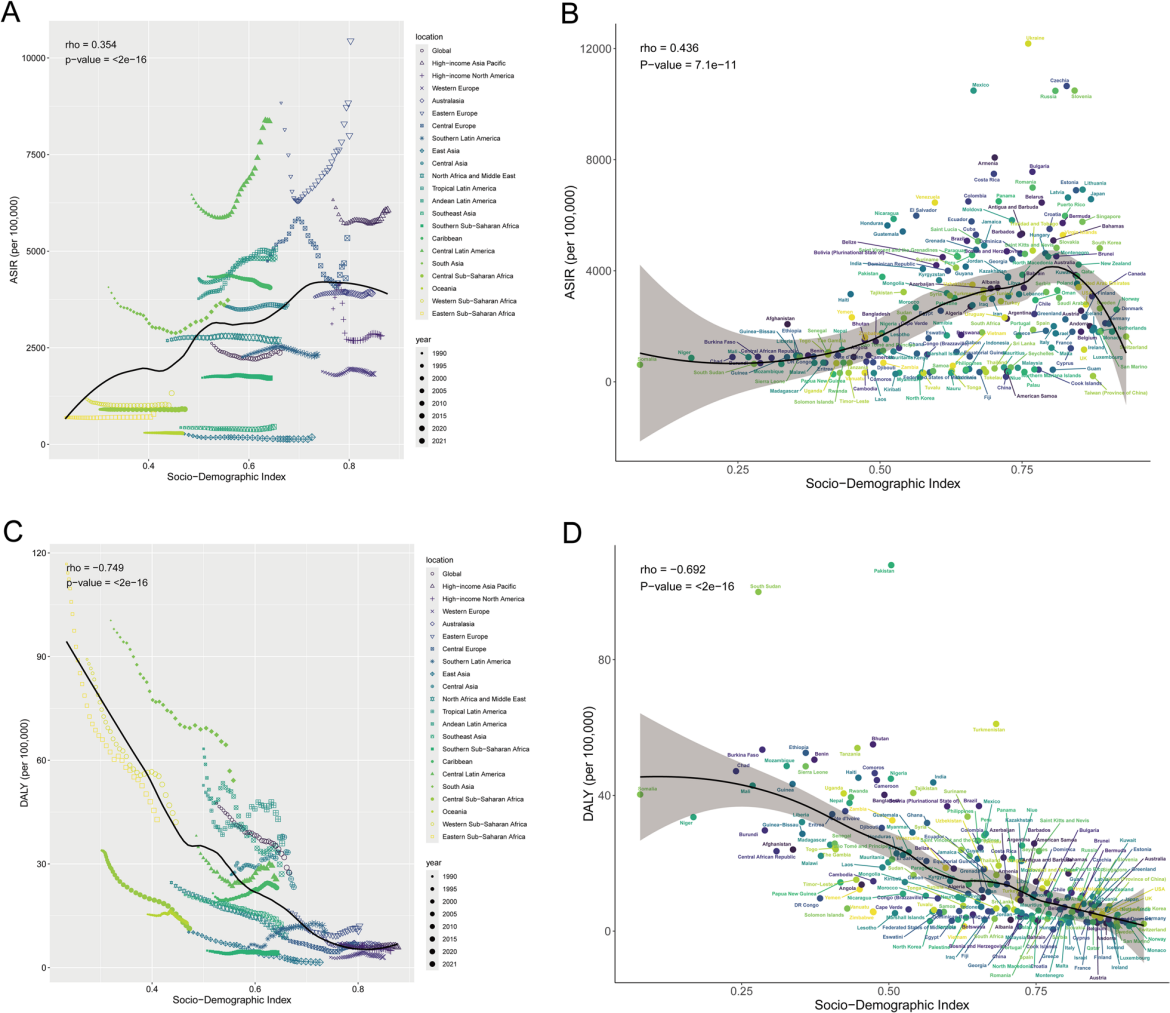
Age-standardised incidence rates (**A**) and age-standardised DALYs rates (**C**) per 100,000 population of pediatric urinary tract infections for 21 GBD regions by SDI, 1990–2021. Age-standardised incidence rates (**B**) and age-standardised DALYs rates (**D**) per 100,000 population of pediatric urinary tract infections in 204 countries or territories worldwide from 1990 to 2021 by SDI. *SDI* socio-demographic index, *DALYs* disability-adjusted life-years, *GBD* Global Burden of Diseases, Injuries, and Risk Factors Study

According to GBD 2021, a total of 204 countries or territories worldwide have recorded pediatric urinary tract infections, causing urinary tract infections-associated DALYs in 204 countries or territories (Fig. 4).

In 2021, India had the highest incidence of urinary tract infections, accounting for almost 1/3 of the total global incidence of paediatric UTIs (14,911,593/45,485,831 = 32.78%), followed by Mexico (3,363,520) and Pakistan (3,230,181). These three countries accounted for 47.28% of the total dengue incidence among children and adolescents worldwide (Table S1). Most of the countries or regions (182 out of 204) showed an increase in the incidence of urinary tract infections, with Qatar showing the highest increase of 609% from 1990 to 2021, followed by the United Arab Emirates (412%) and Jordan (323%) (Table S1). In 2021, the highest ASIR for pediatric urinary tract infections country was Ukraine (12182.70 cases per 100,000 population), followed by Czechia (10653.78 cases per 100,000 population), and Slovenia (10489.10 cases per 100,000 population) (Table S1 and Fig. 4B). A total of 133 countries or territories showed an increasing trend in ASIR between 1990 and 2021. The largest increase was observed in Pakistan (EAPC = 2.91, 95% CI 2.03–3.79), followed by Slovenia (EAPC = 2.16, 95% CI 1.55–2.76) and Mexico (EAPC = 1.89, 95% CI 1.37–2.40) (Table S1 and Fig. 3B). 71 countries or regions showed a decreasing trend in pediatric ASIR, mainly in Asia, such as China, India, and Indonesia (Table S1 and Fig. 3B).

In 2021, the number of DALYs associated with urinary tract infections was highest in India (160,543), followed by Pakistan (92,162) and Nigeria (45,633), which cumulatively accounted for 53.83% of the global burden of pediatric DALYs associated with urinary tract infections (Table S3). The burden of DALYs due to urinary tract infections in 2021. The highest country was Pakistan with 1.23 ASDRs per 100,000 people in 2021, followed by South Sudan with 1.15 ASDRs per 100,000 people. The remaining countries or regions had ASDRs below 0.7 per 100,000 people (Table S2). From 1990 to 2021, 37 of the countries or territories with documented DALYs due to urinary tract infections showed an increasing trend in ASDR (37 out of 204 countries), with the largest increase in Kuwait (EAPC = 456%, 95% CI 2.74–6.42), followed by Dominica (EAPC = 392%, 95% CI 3.65–4.19) and Argentina (EAPC = 388%, 95% CI 3.18–4.58) (Table S2). A total of 167 countries or regions showed a decreasing trend in ASDR, mainly in East and West Asia, such as China, Qatar, and Saudi Arabia (Fig. 3D).

### Future forecasts of global burden of pediatric urinary tract infections

**Fig. 5:**
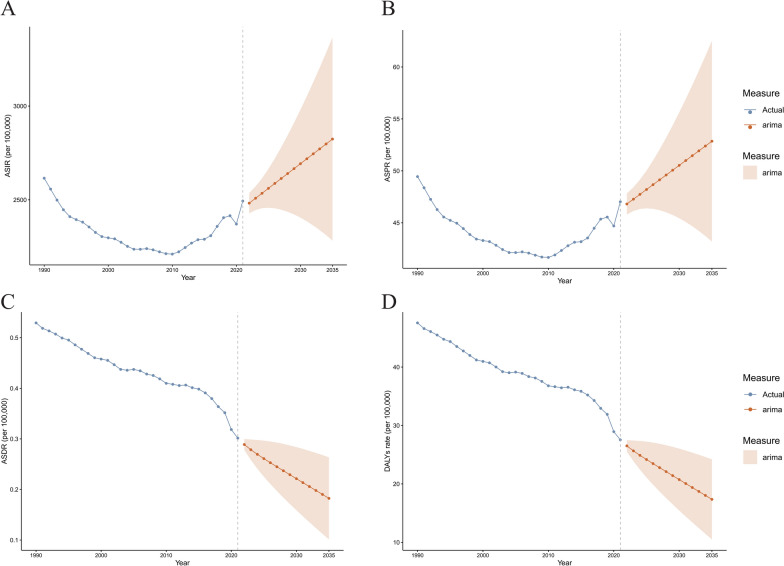
Temporal trends of ASPR, ASDR, ASIR, and DALY rates across different regions. The solid blue line represents the observed values, the dashed orange line represents the mean, and the light orange shaded area represents the fluctuation range of the measurements. Each subplot corresponds to a specific region, with the x-axis representing time and the y-axis indicating the number of events or indicators per 100,000 population. Temporal trends of the ASIR (**A**), ASPR (**B**), ASDR (**C**), and DALYs rates (**D**) globally. *ASIR* age-standardized incidence rate, *ASPR* age-standardized prevalence rate, *ASDR* age-standardized DALY rate, *DALYs* disability-adjusted life-years

This study employed an Autoregressive Integrated Moving Average (ARIMA) model to project significant increases in ASIR and ASPR for pediatric urinary tract infections from 2022 to 2035, while the ASDR and DALYs rate are expected to decrease significantly. Globally, the ASIR in 2035 is projected to be 2,824.18 per 100,000 population (95% CI 2,283.17–3,365.18). The ASPR in 2035 is projected to be 52.85 per 100,000 population (95% CI 43.19–62.51). The ASDR in 2035 is expected to remain relatively low at 0.18 per 100,000 population (95% CI 0.10–0.26). The DALYs rate in 2035 is projected to be 17.35 per 100,000 population (95% CI 10.50–24.21) (Fig. 5).

## Discussion

Our study found that pediatric urinary tract infections consistently impose a higher disease burden on children and adolescents than on the general population. The incidence of UTIs in this age group has increased from 1990 to 2021, with approximately 50.17 million affected globally in 2021, reflecting a 10% increase since 1990. The age-standardized incidence rate was 2493.89 per 100,000 population, with an estimated annual percentage change of −17%. The highest prevalence and DALYs were observed in regions with low-middle and middle SDI. These findings underscore the global burden of pediatric UTIs, highlighting the need for continued research and intervention to address this growing issue.

Globally, the ASIR of urinary tract infections in children and adolescents declined from 1990 to 2010, followed by a gradual increase. The initial decline may reflect improvements in public health infrastructure [3, 19], whereas the subsequent rise likely relates to increasing antibiotic resistance [20]. The morbidity and mortality burden of UTIs is substantially higher in children than in other age groups [21], consistent with our findings that the global prevalence of pediatric UTIs exceeds that of the general population, highlighting the need for greater attention to this issue.

Among children and adolescents, those aged 2–4 years exhibit the highest prevalence of UTIs, although this group experiences a lower burden of DALYs. This may be due to the incomplete development of the urinary system in this age group, as well as the shorter urethra, which increases susceptibility to bacterial contamination [22]. Furthermore, children in this age range are often not fully aware of hygiene practices, contributing to the higher prevalence of infections [23]. Daniel M et al. [22] confirm that children aged 2–4 years have a higher incidence of lower urinary tract infections with milder symptoms, resulting in a lower DALY burden.

Our study indicates that pediatric urinary tract infections pose the greatest threat in South Asia, where the burden of morbidity and DALYs is highest. Within this region, India bears a significant burden of UTI-related diseases [3]. The prevalence of pediatric UTIs in both South and North India surpasses that of the general population, and similar trends have been reported in Nepal, where UTI cases are increasing [24]. This is largely due to poor sanitation and a high prevalence of congenital urinary tract anomalies [25, 26]. From 1990 to 2021, ASIR of UTIs in children and adolescents increased across nine regions, with the most significant rise observed in tropical Latin America. In this region, antibiotic resistance has exacerbated the situation, as UTI pathogens show high resistance to commonly used antibiotics, driving the increase in morbidity [19]. Regarding mortality, southern Latin America showed the greatest rise in the ASDR for pediatric UTIs, while East Asia experienced the most substantial decrease.

Our study revealed a slightly different distribution pattern of the pediatric UTI burden across SDI regions compared to the general population. Specifically, the burden was highest in low-middle SDI regions and showed the greatest reduction in high and high-middle SDI regions. Zi H et al. found that low-middle SDI regions had higher morbidity, mortality, and disability-adjusted life years associated with UTIs, which aligns with our findings [27]. A population-wide study also identified the highest UTI burden in low SDI areas, with rising age-standardized incidence and death rates [19]. In contrast, our study observed a decrease in both ASIR and ASDR across most regions. This discrepancy may be attributed to our more comprehensive data selection and the increased focus on pediatric UTI research in recent years. Additionally, economic development has improved healthcare infrastructure, and the implementation of stricter diagnostic criteria for UTIs [28] has reduced misdiagnosis and overdiagnosis.

Urinary tract infections are common bacterial infections [29, 30], with the pediatric population at higher risk due to their underdeveloped anatomy and immune systems. Nearly 8% of girls and 2% of boys experience UTIs [31]. Waller TA et al. found that antibiotics are the most frequently prescribed treatment for UTIs [32]. However, recent evidence shows significant antibiotic resistance, particularly against Escherichia coli and Enterococci, the primary causative organisms [33]. The rising resistance, coupled with the growing burden of pediatric UTIs, underscores the urgent need to enhance health education and disease prevention, especially in regions with inadequate healthcare infrastructure.

Pediatric urinary tract infections are common bacterial infections in children [34] and can lead to serious complications, imposing a significant economic burden on society [35]. Treatment goals include symptomatic relief, infection eradication, recurrence prevention, and kidney damage reduction. While sepsis rates are declining, severe UTIs are on the rise. Antimicrobial resistance poses a major challenge to effective treatment [36]. Treatment regimens should be tailored to the infection type and clinical features. For acute cystitis, oral antibiotics (e.g., amoxicillin/clavulanate potassium) are preferred [37]. Although a 7–14 day treatment course has been standard, recent studies suggest 3–7 day courses may be effective for uncomplicated cases, adjusted for age and drug resistance risk [38]. For acute pyelonephritis, initial intravenous antibiotics (e.g., ceftriaxone or piperacillin/tazobactam) should be chosen based on severity. Once fever subsides, oral antibiotics can be used for sequential therapy, typically over 10–14 days. Severe or drug-resistant infections may require up to 21 days of treatment [39, 40]. Symptomatic treatment includes NSAIDs for fever and pain, along with rehydration (≥ 2,000 mL per day) to aid bacterial clearance [41]. Recurrent infections should prompt investigation of anatomical urinary tract abnormalities, and long-term low-dose antibiotics (e.g., nitrofurantoin or cefuroxime) may be considered to prevent recurrence [42]. In resource-limited areas, rising antibiotic resistance and slow vaccine development complicate treatment, emphasizing the need for optimized regimens tailored to local resistance profiles and cost-effective prophylactic strategies [43].

However, this study has several limitations. First, our analysis is dependent on the availability and quality of raw data from GBD 2021. As with all GBD studies, limitations in methodology may introduce bias into our estimates [19, 44, 45]. Data from different regions and countries vary in quality, comparability, accuracy, and completeness [46]. In regions with high urinary tract infection prevalence, limitations in surveillance systems and reporting mechanisms hinder data accuracy and comprehensiveness, which could be improved. Previous studies have also noted discrepancies between GBD estimates and reported cases of urinary tract infections [4, 47], highlighting the need for more comprehensive analyses in areas with significant differences. Second, we lacked access to serological data for urinary tract infections, which is critical for understanding the global spread and epidemiology of the disease and for developing vaccine policies [48–50]. Third, we used linear regression, following methods in previous studies [19, 51], to assess long-term trends in pediatric urinary tract infection burden from 1990 to 2021. However, regardless of the model chosen, there is a risk of model misspecification. Assuming a linear relationship between the natural logarithm of the ASR and calendar year may not capture the complexities of trends, such as inflection points or abrupt changes. Alternative methods, such as joinpoint regression, may identify these nuances. Future analyses should consider more flexible modeling approaches to better understand temporal trends in urinary tract infection burden. Additionally, consistent with previous studies [19, 51, 52], we based our trend estimates on the annual percent change and its 95% CI. However, caution is needed in interpretation to avoid over-relying on significance tests. Even when the 95% CI for certain changes exceeds zero or the p-value nears 0.05 (or other thresholds) [53, 54], the practical significance of the change in public health or clinical practice remains crucial. Fourth, while GBD 2021 data remain widely used and robust, the release of GBD 2023 may lead to updated estimates and refined methodologies that could affect projections and trends. Thus, the findings should be understood in the context of GBD 2021, with future studies incorporating GBD 2023 data potentially yielding different results. Ongoing research is necessary to examine the impact of these updates on pediatric UTI trends worldwide. Finally, although we subdivided the data by age, the classifications were based on GBD-defined age groups, which may not capture the full range of differences in urinary tract infections among pediatric patients. More precise age categories could provide additional insights for vaccine policy. Standardizing reporting mechanisms and refining age group classifications are essential for more accurately quantifying the burden of urinary tract infections in pediatric populations, which could inform more effective vaccination and prevention strategies.

Despite these limitations, our study offers valuable insights into the global burden of pediatric urinary tract infections and serves as a foundation for future research and policy development focused on preventing and managing pediatric UTIs. We recognize these constraints to encourage careful interpretation of our findings and highlight areas for improvement in future studies. To the best of our knowledge, this GBD-based study represents the most comprehensive effort to date in analyzing the global burden of childhood UTIs, covering incidence, prevalence, mortality, and DALYs, while also evaluating the role of various risk factors and projecting future trends.

## Conclusion

This study comprehensively assessed the global burden and temporal trends of pediatric urinary tract infections (UTIs) from 1990 to 2021, revealing a significant and growing public health challenge. Despite a slight decline in age-standardized incidence rates (ASIR) over the study period, the overall incidence of pediatric UTIs increased by 10%, with 50,173,655 cases reported globally in 2021. Notably, the burden of disability-adjusted life years (DALYs) associated with pediatric UTIs decreased by 33%, indicating some progress in reducing the severity of the disease. However, the highest incidence and DALY burden were observed in regions with low-middle and middle Socio-Demographic Index (SDI), particularly in South Asia and Sub-Saharan Africa, highlighting disparities in healthcare infrastructure and access. The findings underscore the urgent need for targeted public health interventions and improved antibiotic stewardship to mitigate the impact of pediatric UTIs, especially in high-burden regions. Future research should focus on enhancing surveillance systems, addressing antibiotic resistance, and developing region-specific prevention strategies to reduce the global burden of pediatric UTIs.

## Supplementary Information

Supplementary material 1.

## Data Availability

The data used in this study are available free of charge online at https://vizhub.healthdata.org/gbd-results on request. The datasets used and/or analysed during the current study available from the corresponding author on reasonable request.
