# Supplementary material for: Temporal trends and geographical variations in pediatric urinary tract infections: a comprehensive analysis using the global burden of disease study 2021
Source: Trop Med Health. 2025 Nov 24;53:170. doi: 10.1186/s41182-025-00829-y (PMC12642102; doi:10.1186/s41182-025-00829-y)
Supplement: Supplementary file 1 — Supplementary material 1. [file 41182_2025_829_MOESM1_ESM.docx]

**Table S1.** The incidence cases and ASIR of Pediatric Urinary Tract Infection in 1990 and 2021, with Temporal Trends from 1990 to 2021 in 204 countries or territories.

| **location** | **Num_1990** | **ASIR_1990** | **Num_2021** | **ASIR_2021** | **Num_change** | **EAPC_ASIR** |
| --- | --- | --- | --- | --- | --- | --- |
| Afghanistan | 91,438 (76,061 - 109,412) | 2122.48 (1765.54 - 2539.71) | 294,933 (245,565 - 361,356) | 2076.90 (1729.26 - 2544.65) | 2.19 (1.84 - 2.64) | 0.17 (0.10 - 0.24) |
| Albania | 39,705 (33,561 - 45,994) | 3553.78 (3003.87 - 4116.71) | 15,040 (12,847 - 17,698) | 3389.77 (2895.50 - 3988.99) | -0.04 (-0.14 - 0.05) | -0.07 (-0.15 - 0.01) |
| Algeria | 291,869 (244,034 - 349,317) | 2721.48 (2275.45 - 3257.15) | 360,817 (296,253 - 431,703) | 2712.56 (2227.18 - 3245.48) | 0.89 (0.69 - 1.08) | -0.09 (-0.18 to -0.01) |
| American Samoa | 79 (67 - 92) | 414.25 (351.18 - 484.35) | 53 (44 - 63) | 376.03 (312.71 - 445.80) | 0.33 (0.19 - 0.49) | -0.34 (-0.36 to -0.32) |
| Andorra | 181 (150 - 218) | 1902.36 (1574.03 - 2292.01) | 204 (166 - 249) | 2004.15 (1634.82 - 2454.04) | 0.50 (0.32 - 0.69) | 0.14 (0.04 - 0.24) |
| Angola | 41,790 (35,253 - 49,540) | 886.37 (747.72 - 1050.73) | 136,589 (114,691 - 164,808) | 895.93 (752.29 - 1081.03) | 2.32 (2.00 - 2.73) | -0.03 (-0.06 - 0.00) |
| Antigua and Barbuda | 924 (768 - 1,114) | 5079.34 (4219.95 - 6125.66) | 902 (723 - 1,116) | 5335.15 (4275.46 - 6605.13) | 0.63 (0.46 - 0.82) | 0.04 (-0.02 - 0.09) |
| Argentina | 225,089 (189,734 - 266,421) | 2220.85 (1872.03 - 2628.66) | 231,900 (190,674 - 282,088) | 2277.24 (1872.40 - 2770.09) | 0.59 (0.41 - 0.81) | 0.04 (0.02 - 0.06) |
| Armenia | 41,143 (35,001 - 48,206) | 3943.90 (3355.17 - 4620.93) | 47,856 (41,408 - 54,860) | 8078.73 (6990.32 - 9261.09) | 0.14 (0.01 - 0.28) | 0.44 (-0.02 - 0.91) |
| Australia | 141,869 (119,569 - 168,944) | 3747.38 (3158.33 - 4462.54) | 182,609 (154,942 - 217,654) | 3844.87 (3262.34 - 4582.74) | 0.52 (0.36 - 0.69) | 0.05 (0.02 - 0.07) |
| Austria | 34,185 (26,453 - 46,129) | 2535.31 (1961.91 - 3421.12) | 33,210 (25,793 - 45,548) | 2560.46 (1988.62 - 3511.73) | 0.25 (0.06 - 0.49) | -0.02 (-0.36 - 0.33) |
| Azerbaijan | 84,519 (71,987 - 97,869) | 3482.53 (2966.18 - 4032.63) | 79,349 (67,265 - 93,871) | 3361.42 (2849.51 - 3976.62) | 0.62 (0.46 - 0.82) | 0.07 (0.00 - 0.13) |
| Bahamas | 3,918 (3,230 - 4,754) | 4857.72 (4004.73 - 5893.55) | 4,134 (3,432 - 5,166) | 5091.83 (4226.92 - 6363.95) | 0.69 (0.50 - 0.94) | 0.21 (0.16 - 0.26) |
| Bahrain | 5,350 (4,421 - 6,617) | 3277.00 (2708.27 - 4053.15) | 10,054 (8,216 - 12,474) | 3388.53 (2769.10 - 4204.19) | 2.03 (1.73 - 2.35) | 0.11 (0.07 - 0.15) |
| Bangladesh | 672,497 (566,094 - 797,417) | 1374.93 (1157.39 - 1630.33) | 662,324 (545,445 - 801,434) | 1447.20 (1191.81 - 1751.16) | 1.09 (0.86 - 1.36) | 0.22 (0.20 - 0.25) |
| Barbados | 3,257 (2,701 - 4,020) | 5223.12 (4331.39 - 6446.99) | 2,493 (2,029 - 3,009) | 5293.54 (4307.72 - 6390.05) | 0.28 (0.12 - 0.43) | 0.04 (0.03 - 0.06) |
| Belarus | 156,561 (133,536 - 181,388) | 6512.87 (5555.03 - 7545.65) | 101,898 (84,939 - 120,152) | 6456.67 (5382.11 - 7613.34) | -0.06 (-0.14 - 0.04) | 0.22 (0.14 - 0.30) |
| Belgium | 32,454 (26,719 - 38,886) | 1796.86 (1479.34 - 2153.01) | 34,557 (28,501 - 40,794) | 1807.29 (1490.54 - 2133.46) | 0.11 (-0.01 - 0.26) | 0.26 (-0.08 - 0.60) |
| Belize | 3,493 (2,954 - 4,209) | 4266.70 (3608.20 - 5141.71) | 5,530 (4,512 - 6,832) | 4491.32 (3664.84 - 5549.00) | 1.72 (1.45 - 2.03) | 0.18 (0.16 - 0.20) |
| Benin | 22,159 (18,250 - 25,767) | 914.97 (753.59 - 1063.97) | 57,913 (49,018 - 69,035) | 952.45 (806.17 - 1135.36) | 2.04 (1.75 - 2.35) | 0.12 (0.09 - 0.14) |
| Bermuda | 662 (562 - 790) | 5552.86 (4714.00 - 6624.54) | 483 (391 - 586) | 5720.31 (4638.94 - 6944.80) | 0.03 (-0.09 - 0.16) | 0.08 (0.06 - 0.10) |
| Bhutan | 3,774 (3,231 - 4,427) | 1439.55 (1232.37 - 1688.33) | 2,828 (2,322 - 3,454) | 1511.06 (1240.50 - 1845.19) | 0.63 (0.48 - 0.82) | 0.22 (0.18 - 0.25) |
| Bolivia (Plurinational State of) | 107,766 (89,927 - 129,694) | 4012.36 (3348.19 - 4828.81) | 146,395 (121,186 - 178,900) | 4198.88 (3475.83 - 5131.19) | 1.22 (0.95 - 1.50) | 0.17 (0.12 - 0.22) |
| Bosnia and Herzegovina | 52,079 (44,695 - 60,397) | 4753.47 (4079.56 - 5512.71) | 23,051 (19,456 - 27,067) | 4700.03 (3967.01 - 5518.82) | -0.20 (-0.29 to -0.11) | 0.10 (0.02 - 0.18) |
| Botswana | 9,985 (8,497 - 11,701) | 1691.04 (1439.07 - 1981.78) | 11,800 (10,072 - 13,959) | 1689.85 (1442.46 - 1999.07) | 1.18 (0.92 - 1.45) | 0.32 (-0.12 - 0.77) |
| Brazil | 1,946,705 (1,694,125 - 2,261,888) | 3747.67 (3261.42 - 4354.44) | 2,446,354 (2,135,673 - 2,825,830) | 5076.97 (4432.21 - 5864.50) | 0.68 (0.61 - 0.75) | 1.16 (1.05 - 1.27) |
| Brunei Darussalam | 4,290 (3,707 - 5,012) | 4736.15 (4092.07 - 5533.03) | 4,278 (3,627 - 5,012) | 4522.67 (3834.42 - 5298.13) | 0.76 (0.60 - 0.96) | -0.17 (-0.19 to -0.14) |
| Bulgaria | 67,497 (58,793 - 78,655) | 3887.59 (3386.26 - 4530.23) | 73,839 (63,588 - 85,366) | 7565.01 (6514.74 - 8745.97) | -0.09 (-0.19 - 0.03) | 0.59 (0.18 - 1.01) |
| Burkina Faso | 42,098 (35,364 - 49,576) | 892.08 (749.39 - 1050.55) | 93,657 (79,671 - 113,277) | 902.96 (768.12 - 1092.13) | 1.57 (1.29 - 1.87) | 0.05 (0.03 - 0.08) |
| Burundi | 16,896 (14,420 - 20,299) | 644.62 (550.15 - 774.45) | 39,421 (31,957 - 49,088) | 673.38 (545.89 - 838.51) | 1.49 (1.25 - 1.78) | 0.04 (-0.01 - 0.10) |
| Cabo Verde | 1,888 (1,610 - 2,215) | 1200.37 (1023.55 - 1408.07) | 1,750 (1,485 - 2,087) | 1222.32 (1036.82 - 1457.49) | 0.99 (0.78 - 1.24) | 0.08 (0.05 - 0.12) |
| Cambodia | 14,885 (12,381 - 17,706) | 319.36 (265.63 - 379.88) | 16,901 (13,932 - 20,960) | 330.33 (272.30 - 409.65) | 1.32 (1.05 - 1.64) | 0.10 (0.04 - 0.15) |
| Cameroon | 46,451 (40,259 - 54,558) | 951.46 (824.64 - 1117.51) | 129,196 (109,370 - 154,658) | 959.36 (812.14 - 1148.43) | 2.24 (1.83 - 2.67) | 0.03 (0.01 - 0.06) |
| Canada | 174,831 (149,745 - 208,326) | 3039.65 (2603.49 - 3621.99) | 190,215 (159,785 - 228,625) | 3081.99 (2588.95 - 3704.34) | 0.54 (0.38 - 0.69) | -0.04 (-0.06 to -0.01) |
| Central African Republic | 10,295 (8,684 - 12,155) | 841.98 (710.28 - 994.12) | 19,698 (16,377 - 24,435) | 862.49 (717.07 - 1069.93) | 1.14 (0.90 - 1.41) | 0.06 (0.04 - 0.08) |
| Chad | 26,163 (22,540 - 31,154) | 894.03 (770.25 - 1064.61) | 80,321 (66,771 - 95,845) | 890.98 (740.67 - 1063.19) | 1.86 (1.56 - 2.16) | -0.00 (-0.02 - 0.02) |
| Chile | 93,643 (77,947 - 110,373) | 2357.63 (1962.47 - 2778.84) | 89,176 (74,336 - 107,877) | 2441.94 (2035.58 - 2954.03) | 0.61 (0.43 - 0.81) | -0.10 (-0.55 - 0.35) |
| China | 882,263 (769,234 - 1,054,403) | 277.11 (241.61 - 331.18) | 484,098 (417,348 - 554,697) | 186.46 (160.75 - 213.65) | 0.45 (0.37 - 0.55) | -1.65 (-2.03 to -1.27) |
| Colombia | 756,547 (642,069 - 899,961) | 6486.73 (5505.17 - 7716.37) | 689,671 (570,984 - 849,091) | 6498.35 (5380.03 - 8000.46) | 0.66 (0.49 - 0.82) | 0.02 (-0.00 - 0.04) |
| Comoros | 1,535 (1,289 - 1,826) | 721.61 (606.20 - 858.68) | 1,753 (1,425 - 2,159) | 729.85 (593.50 - 899.02) | 1.01 (0.81 - 1.23) | 0.09 (0.06 - 0.11) |
| Congo | 10,366 (8,706 - 12,318) | 984.50 (826.78 - 1169.84) | 18,977 (15,696 - 22,396) | 983.65 (813.55 - 1160.87) | 1.58 (1.31 - 1.87) | -0.08 (-0.10 to -0.05) |
| Cook Islands | 28 (24 - 33) | 430.84 (356.64 - 502.72) | 17 (14 - 20) | 438.70 (372.40 - 521.02) | 0.28 (0.14 - 0.44) | -0.02 (-0.06 - 0.02) |
| Costa Rica | 85,660 (71,715 - 101,292) | 7619.67 (6379.28 - 9010.23) | 76,204 (63,871 - 90,401) | 7490.82 (6278.53 - 8886.38) | 0.65 (0.49 - 0.82) | -0.01 (-0.03 - 0.01) |
| Côte d'Ivoire | 54,707 (46,389 - 64,149) | 959.22 (813.37 - 1124.76) | 111,114 (92,966 - 132,571) | 960.21 (803.39 - 1145.64) | 1.53 (1.24 - 1.88) | -0.04 (-0.07 - -0.00) |
| Croatia | 49,845 (42,978 - 57,854) | 5050.07 (4354.35 - 5861.55) | 32,886 (27,618 - 37,621) | 5507.21 (4625.10 - 6300.12) | 0.05 (-0.06 - 0.19) | 0.33 (0.03 - 0.62) |
| Cuba | 131,513 (109,813 - 155,788) | 5252.05 (4385.44 - 6221.48) | 94,155 (78,207 - 115,173) | 5298.49 (4401.00 - 6481.27) | 0.06 (-0.06 - 0.19) | -0.02 (-0.05 - 0.01) |
| Cyprus | 2,399 (2,016 - 2,855) | 1212.03 (1018.51 - 1442.45) | 2,644 (2,240 - 3,218) | 1209.05 (1024.40 - 1471.53) | 0.88 (0.66 - 1.17) | -0.29 (-0.55 to -0.04) |
| Czechia | 110,709 (95,455 - 127,439) | 5023.82 (4331.59 - 5783.01) | 182,856 (155,827 - 209,427) | 10653.78 (9078.99 - 12201.92) | 0.25 (0.11 - 0.38) | 1.71 (1.09 - 2.33) |
| Democratic People's Republic of Korea | 10,417 (8,670 - 12,307) | 175.10 (145.73 - 206.87) | 7,881 (6,526 - 9,449) | 165.09 (136.71 - 197.94) | 0.48 (0.31 - 0.67) | -0.22 (-0.26 to -0.18) |
| Democratic Republic of the Congo | 160,379 (135,943 - 192,291) | 905.90 (767.87 - 1086.16) | 346,672 (292,670 - 417,251) | 912.32 (770.21 - 1098.06) | 1.52 (1.26 - 1.82) | -0.00 (-0.02 - 0.01) |
| Denmark | 18,399 (15,272 - 22,859) | 2082.88 (1728.82 - 2587.77) | 20,141 (16,514 - 24,935) | 2110.87 (1730.78 - 2613.30) | 0.10 (-0.04 - 0.23) | 0.10 (-0.15 - 0.35) |
| Djibouti | 1,237 (1,046 - 1,459) | 710.48 (600.68 - 838.08) | 2,910 (2,416 - 3,528) | 704.35 (584.77 - 853.84) | 2.63 (2.26 - 3.00) | -0.12 (-0.15 to -0.08) |
| Dominica | 1,088 (919 - 1,300) | 4384.54 (3703.49 - 5238.52) | 633 (514 - 787) | 4622.99 (3759.89 - 5750.63) | 0.01 (-0.09 - 0.13) | 0.11 (0.07 - 0.14) |
| Dominican Republic | 107,335 (89,666 - 130,059) | 3982.08 (3326.58 - 4825.11) | 117,554 (98,072 - 143,723) | 4000.55 (3337.56 - 4891.10) | 0.67 (0.49 - 0.87) | -0.00 (-0.06 - 0.05) |
| Ecuador | 161,619 (137,249 - 192,163) | 4181.30 (3550.81 - 4971.51) | 292,930 (236,566 - 356,104) | 5776.70 (4665.19 - 7022.53) | 1.44 (1.07 - 1.79) | 1.79 (1.49 - 2.09) |
| Egypt | 557,951 (464,315 - 667,936) | 2515.36 (2093.23 - 3011.20) | 943,265 (763,002 - 1,146,079) | 2559.38 (2070.27 - 3109.68) | 1.00 (0.83 - 1.20) | -0.02 (-0.07 - 0.02) |
| El Salvador | 127,820 (105,204 - 152,215) | 5922.83 (4874.86 - 7053.22) | 108,838 (91,347 - 131,287) | 5984.07 (5022.38 - 7218.37) | 0.36 (0.23 - 0.50) | 0.06 (0.03 - 0.09) |
| Equatorial Guinea | 1,782 (1,493 - 2,167) | 905.21 (758.19 - 1100.71) | 5,341 (4,410 - 6,399) | 913.01 (753.89 - 1093.87) | 2.84 (2.44 - 3.30) | 0.02 (0.01 - 0.04) |
| Eritrea | 10,662 (8,871 - 12,762) | 669.78 (557.25 - 801.70) | 17,324 (14,495 - 21,343) | 686.19 (574.16 - 845.41) | 1.26 (1.03 - 1.54) | 0.07 (0.05 - 0.08) |
| Estonia | 24,010 (20,220 - 28,153) | 6877.57 (5791.94 - 8064.27) | 14,914 (12,869 - 17,393) | 6900.46 (5954.09 - 8047.49) | -0.19 (-0.29 to -0.09) | 0.24 (0.13 - 0.34) |
| Eswatini | 6,042 (5,060 - 7,179) | 1566.24 (1311.85 - 1861.06) | 6,488 (5,388 - 7,802) | 1572.41 (1305.74 - 1890.80) | 0.68 (0.49 - 0.87) | -0.07 (-0.11 to -0.03) |
| Ethiopia | 170,231 (147,014 - 198,781) | 698.70 (603.41 - 815.89) | 418,414 (356,652 - 491,187) | 943.43 (804.17 - 1107.51) | 1.42 (1.29 - 1.57) | 0.21 (0.05 - 0.38) |
| Fiji | 981 (826 - 1,160) | 348.49 (293.63 - 412.05) | 953 (787 - 1,139) | 349.75 (288.82 - 418.04) | 0.46 (0.27 - 0.65) | 0.11 (0.08 - 0.14) |
| Finland | 21,569 (17,813 - 25,747) | 2235.21 (1846.02 - 2668.28) | 19,833 (16,561 - 24,375) | 2341.32 (1955.00 - 2877.51) | 0.05 (-0.07 - 0.18) | -0.01 (-0.16 - 0.15) |
| France | 200,548 (167,000 - 243,399) | 1712.00 (1425.62 - 2077.81) | 209,341 (172,649 - 249,476) | 1803.48 (1487.37 - 2149.24) | 0.09 (-0.03 - 0.23) | 0.12 (0.09 - 0.16) |
| Gabon | 4,300 (3,662 - 5,257) | 1055.22 (898.69 - 1289.99) | 6,890 (5,766 - 8,217) | 1077.94 (902.10 - 1285.70) | 1.13 (0.94 - 1.35) | 0.08 (0.05 - 0.10) |
| Gambia | 4,850 (4,128 - 5,610) | 1051.43 (894.85 - 1216.14) | 10,630 (8,880 - 12,641) | 1070.00 (893.89 - 1272.47) | 1.74 (1.49 - 2.04) | -0.00 (-0.04 - 0.03) |
| Georgia | 55,925 (47,374 - 65,588) | 4085.93 (3461.21 - 4791.95) | 32,493 (27,978 - 37,398) | 4415.21 (3801.66 - 5081.77) | -0.30 (-0.36 to -0.24) | 0.37 (0.29 - 0.44) |
| Germany | 263,142 (221,406 - 320,746) | 2032.64 (1710.25 - 2477.60) | 250,281 (205,186 - 302,926) | 2091.85 (1714.94 - 2531.84) | -0.00 (-0.11 - 0.12) | 0.34 (0.08 - 0.61) |
| Ghana | 68,996 (59,259 - 80,051) | 1027.26 (882.27 - 1191.85) | 131,074 (111,378 - 154,171) | 1017.41 (864.53 - 1196.69) | 1.61 (1.39 - 1.90) | 0.01 (-0.02 - 0.05) |
| Greece | 36,874 (29,894 - 44,959) | 1822.22 (1477.28 - 2221.78) | 25,640 (21,285 - 31,420) | 1838.31 (1526.05 - 2252.74) | -0.07 (-0.17 - 0.06) | -0.05 (-0.09 to -0.01) |
| Greenland | 315 (263 - 385) | 2217.71 (1846.59 - 2706.86) | 265 (216 - 318) | 2251.31 (1837.80 - 2708.05) | 0.18 (0.07 - 0.33) | -0.04 (-0.06 to -0.01) |
| Grenada | 1,537 (1,276 - 1,865) | 4600.55 (3818.29 - 5582.30) | 1,038 (855 - 1,300) | 4756.52 (3914.54 - 5956.81) | 0.35 (0.22 - 0.50) | 0.13 (0.08 - 0.17) |
| Guam | 186 (158 - 217) | 446.37 (378.23 - 521.39) | 159 (137 - 187) | 435.58 (374.19 - 510.28) | 0.46 (0.29 - 0.67) | -0.08 (-0.15 to -0.02) |
| Guatemala | 216,311 (181,189 - 261,918) | 5326.25 (4461.42 - 6449.23) | 267,166 (221,067 - 328,710) | 5414.77 (4480.47 - 6662.10) | 1.00 (0.79 - 1.25) | 0.05 (0.02 - 0.07) |
| Guinea | 24,495 (20,812 - 28,891) | 890.13 (756.29 - 1049.89) | 53,887 (44,534 - 64,682) | 891.32 (736.61 - 1069.87) | 1.29 (1.04 - 1.56) | 0.02 (-0.03 - 0.07) |
| Guinea-Bissau | 4,431 (3,694 - 5,290) | 918.45 (765.67 - 1096.60) | 8,341 (7,128 - 9,696) | 928.65 (793.66 - 1079.57) | 1.27 (1.01 - 1.58) | 0.01 (-0.03 - 0.06) |
| Guyana | 11,984 (9,940 - 14,272) | 4076.96 (3381.88 - 4855.44) | 8,856 (7,154 - 10,888) | 4150.30 (3352.47 - 5102.31) | 0.09 (-0.03 - 0.23) | 0.14 (0.08 - 0.20) |
| Haiti | 84,736 (68,494 - 101,542) | 3123.23 (2524.56 - 3742.67) | 137,439 (110,111 - 167,193) | 3157.51 (2529.68 - 3841.07) | 1.26 (1.03 - 1.55) | 0.02 (-0.02 - 0.05) |
| Honduras | 126,731 (103,331 - 150,744) | 5735.90 (4676.82 - 6822.74) | 184,550 (152,995 - 218,839) | 5631.38 (4668.52 - 6677.69) | 1.21 (1.00 - 1.47) | 0.00 (-0.02 - 0.03) |
| Hungary | 92,487 (80,359 - 105,626) | 4339.95 (3770.84 - 4956.45) | 64,301 (54,757 - 74,743) | 4630.94 (3943.59 - 5382.97) | -0.04 (-0.17 - 0.06) | 0.18 (0.15 - 0.20) |
| Iceland | 1,197 (1,008 - 1,400) | 1886.98 (1589.19 - 2207.29) | 1,313 (1,094 - 1,570) | 1944.43 (1619.53 - 2324.77) | 0.35 (0.19 - 0.53) | 0.18 (0.06 - 0.31) |
| India | 14,963,826 (12,606,318 - 18,324,818) | 4582.81 (3860.80 - 5612.14) | 14,911,593 (13,003,210 - 17,040,192) | 4069.77 (3548.92 - 4650.72) | 1.01 (0.91 - 1.10) | -0.95 (-1.33 to -0.57) |
| Indonesia | 305,302 (261,161 - 362,868) | 450.72 (385.55 - 535.70) | 309,380 (267,557 - 364,041) | 459.78 (397.63 - 541.02) | 0.65 (0.55 - 0.76) | -0.71 (-1.11 to -0.31) |
| Iran (Islamic Republic of) | 728,237 (623,931 - 851,248) | 2868.82 (2457.92 - 3353.41) | 602,986 (514,085 - 710,774) | 2988.12 (2547.57 - 3522.27) | 0.67 (0.56 - 0.77) | -0.18 (-0.34 to -0.02) |
| Iraq | 222,631 (187,144 - 266,106) | 2702.99 (2272.15 - 3230.82) | 371,465 (305,625 - 457,622) | 2759.38 (2270.30 - 3399.39) | 1.43 (1.19 - 1.69) | 0.11 (0.08 - 0.14) |
| Ireland | 17,891 (14,551 - 21,442) | 1820.94 (1481.01 - 2182.38) | 18,479 (15,035 - 22,209) | 1853.07 (1507.68 - 2227.14) | 0.41 (0.24 - 0.65) | -0.13 (-0.20 to -0.06) |
| Israel | 26,305 (21,911 - 31,389) | 1715.82 (1429.23 - 2047.51) | 45,895 (38,417 - 55,052) | 1746.55 (1461.97 - 2095.03) | 0.94 (0.72 - 1.20) | -0.01 (-0.04 - 0.02) |
| Italy | 199,204 (169,531 - 240,261) | 2158.27 (1836.79 - 2603.10) | 152,344 (133,254 - 174,996) | 2004.55 (1753.37 - 2302.61) | -0.43 (-0.47 to -0.39) | -0.01 (-0.36 - 0.34) |
| Jamaica | 41,272 (34,856 - 49,443) | 4941.56 (4173.36 - 5919.87) | 28,674 (23,665 - 35,076) | 4910.47 (4052.78 - 6006.88) | 0.29 (0.15 - 0.43) | 0.02 (-0.01 - 0.05) |
| Japan | 1,642,262 (1,454,592 - 1,836,869) | 7112.74 (6299.93 - 7955.60) | 1,016,276 (902,408 - 1,148,989) | 6580.24 (5842.96 - 7439.54) | 0.03 (-0.04 - 0.09) | -0.04 (-0.17 - 0.08) |
| Jordan | 46,943 (38,924 - 55,490) | 2873.97 (2383.00 - 3397.20) | 137,626 (100,406 - 207,572) | 3788.19 (2763.70 - 5713.46) | 3.23 (2.59 - 4.50) | 1.10 (0.93 - 1.28) |
| Kazakhstan | 192,857 (165,276 - 222,995) | 3711.48 (3180.68 - 4291.48) | 203,662 (173,548 - 237,461) | 3753.11 (3198.17 - 4375.97) | 0.26 (0.13 - 0.43) | 0.17 (0.11 - 0.23) |
| Kenya | 91,759 (79,725 - 107,304) | 821.46 (713.73 - 960.63) | 204,339 (176,594 - 240,327) | 1094.76 (946.11 - 1287.56) | 1.74 (1.66 - 1.83) | 0.54 (0.31 - 0.77) |
| Kiribati | 86 (73 - 100) | 289.80 (246.28 - 339.51) | 120 (99 - 146) | 286.09 (236.65 - 348.50) | 0.87 (0.63 - 1.10) | -0.01 (-0.03 - 0.02) |
| Kuwait | 19,271 (15,982 - 22,655) | 3476.39 (2883.11 - 4086.74) | 29,894 (24,769 - 36,127) | 3535.75 (2929.56 - 4272.87) | 2.08 (1.78 - 2.55) | 0.09 (0.05 - 0.13) |
| Kyrgyzstan | 61,676 (51,965 - 71,124) | 3676.51 (3097.64 - 4239.67) | 83,204 (71,491 - 97,717) | 3658.30 (3143.29 - 4296.42) | 0.69 (0.54 - 0.91) | 0.09 (0.04 - 0.15) |
| Lao People's Democratic Republic | 5,669 (4,799 - 6,849) | 307.57 (260.36 - 371.61) | 7,287 (6,067 - 9,020) | 317.31 (264.21 - 392.78) | 1.27 (0.99 - 1.57) | 0.12 (0.08 - 0.16) |
| Latvia | 38,190 (32,515 - 44,094) | 6712.28 (5714.90 - 7749.89) | 19,720 (16,612 - 22,928) | 6638.52 (5592.23 - 7718.22) | -0.30 (-0.39 to -0.23) | 0.29 (-0.03 - 0.60) |
| Lebanon | 31,993 (27,285 - 38,196) | 3058.93 (2608.79 - 3652.10) | 40,340 (33,566 - 49,992) | 3156.37 (2626.36 - 3911.55) | 0.96 (0.78 - 1.17) | 0.09 (0.05 - 0.12) |
| Lesotho | 10,477 (8,890 - 12,168) | 1534.88 (1302.45 - 1782.58) | 9,695 (8,166 - 11,778) | 1537.67 (1295.14 - 1868.13) | 0.35 (0.22 - 0.50) | -0.00 (-0.02 - 0.01) |
| Liberia | 10,344 (8,680 - 12,318) | 915.21 (768.01 - 1089.88) | 20,737 (17,080 - 24,859) | 948.67 (781.37 - 1137.24) | 1.49 (1.26 - 1.85) | 0.03 (-0.01 - 0.08) |
| Libya | 54,645 (45,606 - 66,306) | 3017.52 (2518.37 - 3661.44) | 45,990 (37,682 - 56,837) | 3083.21 (2526.24 - 3810.42) | 0.85 (0.64 - 1.07) | 0.02 (-0.01 - 0.05) |
| Lithuania | 58,793 (50,355 - 68,238) | 7077.55 (6061.80 - 8214.56) | 28,233 (24,085 - 32,734) | 6923.52 (5906.28 - 8027.18) | -0.24 (-0.30 to -0.16) | -0.16 (-0.54 - 0.23) |
| Luxembourg | 1,166 (985 - 1,389) | 1764.47 (1490.77 - 2102.08) | 1,816 (1,493 - 2,205) | 1793.82 (1474.81 - 2177.81) | 0.67 (0.48 - 0.87) | 0.06 (-0.10 - 0.22) |
| Madagascar | 36,596 (31,437 - 42,993) | 670.75 (576.19 - 788.01) | 81,082 (65,914 - 101,075) | 691.03 (561.76 - 861.42) | 1.68 (1.40 - 1.97) | 0.04 (0.01 - 0.07) |
| Malawi | 29,825 (25,245 - 35,945) | 655.55 (554.88 - 790.08) | 55,406 (45,220 - 68,769) | 682.01 (556.64 - 846.51) | 1.15 (0.93 - 1.37) | 0.13 (0.10 - 0.17) |
| Malaysia | 32,096 (27,472 - 37,835) | 488.30 (417.95 - 575.61) | 38,561 (31,850 - 47,048) | 506.47 (418.33 - 617.94) | 1.33 (1.07 - 1.61) | 0.05 (0.03 - 0.07) |
| Maldives | 398 (335 - 475) | 378.49 (318.72 - 452.10) | 403 (336 - 486) | 402.47 (335.72 - 485.56) | 2.42 (2.11 - 2.81) | 0.14 (0.05 - 0.23) |
| Mali | 36,760 (31,612 - 43,624) | 890.12 (765.47 - 1056.33) | 102,972 (87,847 - 122,287) | 889.55 (758.88 - 1056.40) | 1.73 (1.43 - 2.04) | 0.01 (-0.01 - 0.04) |
| Malta | 1,104 (921 - 1,301) | 1261.24 (1051.77 - 1485.98) | 787 (663 - 934) | 1229.18 (1035.18 - 1458.95) | 0.13 (0.01 - 0.25) | -0.32 (-0.53 to -0.10) |
| Marshall Islands | 72 (61 - 87) | 330.36 (279.53 - 395.56) | 59 (49 - 71) | 336.01 (280.50 - 406.53) | 0.82 (0.60 - 1.07) | 0.05 (0.03 - 0.07) |
| Mauritania | 9,821 (8,296 - 11,553) | 1062.48 (897.46 - 1249.83) | 20,073 (17,300 - 23,758) | 1083.25 (933.63 - 1282.13) | 1.30 (1.09 - 1.54) | 0.07 (0.05 - 0.09) |
| Mauritius | 1,662 (1,375 - 2,018) | 503.65 (416.52 - 611.43) | 1,082 (883 - 1,313) | 521.46 (425.67 - 632.94) | 0.59 (0.40 - 0.81) | 0.04 (0.01 - 0.07) |
| Mexico | 2,213,561 (1,897,092 - 2,574,159) | 6624.21 (5677.16 - 7703.31) | 3,363,520 (2,909,083 - 3,869,651) | 10489.06 (9071.90 - 12067.41) | 1.12 (1.02 - 1.22) | 1.89 (1.37 - 2.40) |
| Micronesia (Federated States of) | 154 (128 - 182) | 334.87 (278.73 - 396.82) | 104 (87 - 125) | 341.31 (285.70 - 409.95) | 0.37 (0.20 - 0.54) | 0.07 (0.05 - 0.10) |
| Monaco | 61 (51 - 73) | 1744.34 (1456.14 - 2068.97) | 90 (76 - 106) | 1807.90 (1520.88 - 2136.19) | 0.14 (0.01 - 0.27) | 0.18 (0.15 - 0.22) |
| Mongolia | 28,634 (24,380 - 33,482) | 3181.58 (2708.92 - 3720.16) | 34,715 (29,831 - 40,835) | 3194.86 (2745.39 - 3758.05) | 0.68 (0.49 - 0.91) | 0.09 (0.04 - 0.14) |
| Montenegro | 7,309 (6,369 - 8,403) | 4522.85 (3941.38 - 5199.90) | 5,028 (4,408 - 5,845) | 4512.56 (3956.53 - 5246.04) | 0.04 (-0.04 - 0.14) | 0.01 (-0.03 - 0.05) |
| Morocco | 258,623 (218,391 - 307,006) | 2642.85 (2231.72 - 3137.28) | 262,406 (218,571 - 319,150) | 2679.84 (2232.17 - 3259.34) | 0.57 (0.42 - 0.72) | 0.06 (0.03 - 0.09) |
| Mozambique | 40,521 (34,376 - 48,945) | 653.12 (554.07 - 788.91) | 91,958 (76,503 - 112,427) | 644.59 (536.26 - 788.07) | 1.35 (1.11 - 1.62) | -0.00 (-0.05 - 0.04) |
| Myanmar | 47,092 (39,153 - 56,587) | 318.70 (264.97 - 382.96) | 50,724 (41,551 - 62,161) | 324.85 (266.10 - 398.09) | 0.83 (0.63 - 1.10) | -0.00 (-0.03 - 0.02) |
| Namibia | 10,134 (8,589 - 11,778) | 1686.88 (1429.71 - 1960.68) | 13,799 (11,371 - 16,498) | 1671.69 (1377.62 - 1998.68) | 0.98 (0.73 - 1.18) | 0.02 (-0.02 - 0.05) |
| Nauru | 14 (11 - 16) | 321.21 (271.08 - 377.39) | 13 (11 - 15) | 322.70 (270.56 - 382.59) | 0.22 (0.08 - 0.38) | 0.02 (-0.01 - 0.04) |
| Nepal | 101,659 (86,271 - 120,855) | 1206.61 (1023.97 - 1434.45) | 107,164 (92,164 - 128,381) | 1161.39 (998.82 - 1391.33) | 0.86 (0.65 - 1.10) | -0.44 (-0.69 to -0.19) |
| Netherlands | 48,629 (40,647 - 58,553) | 1784.38 (1491.49 - 2148.52) | 49,240 (40,713 - 61,389) | 1835.93 (1518.00 - 2288.91) | 0.07 (-0.06 - 0.21) | 0.11 (0.08 - 0.14) |
| New Zealand | 32,957 (27,725 - 38,536) | 4118.93 (3465.00 - 4816.20) | 41,441 (35,528 - 47,564) | 4221.25 (3618.94 - 4844.98) | 0.72 (0.56 - 0.92) | -0.08 (-0.15 - -0.00) |
| Nicaragua | 109,409 (92,672 - 132,620) | 6007.60 (5088.59 - 7282.11) | 116,208 (95,862 - 140,324) | 5867.85 (4840.51 - 7085.58) | 0.84 (0.60 - 1.06) | -0.01 (-0.03 - 0.02) |
| Niger | 35,237 (29,556 - 42,111) | 867.27 (727.44 - 1036.46) | 110,157 (94,691 - 130,284) | 863.03 (741.86 - 1020.71) | 2.13 (1.84 - 2.55) | -0.05 (-0.07 to -0.02) |
| Nigeria | 595,332 (515,697 - 690,647) | 1521.63 (1318.09 - 1765.25) | 1,782,229 (1,562,752 - 2,026,204) | 1754.35 (1538.31 - 1994.51) | 1.84 (1.75 - 1.92) | -0.50 (-0.92 to -0.07) |
| Niue | 3 (2 - 3) | 348.93 (291.30 - 418.16) | 1 (1 - 2) | 362.94 (300.46 - 432.93) | -0.11 (-0.19 - 0.01) | 0.13 (0.07 - 0.20) |
| North Macedonia | 20,289 (17,307 - 23,716) | 3851.65 (3285.55 - 4502.14) | 12,633 (10,875 - 14,480) | 3856.49 (3319.61 - 4420.19) | 0.17 (0.06 - 0.27) | 0.09 (0.05 - 0.13) |
| Northern Mariana Islands | 60 (50 - 70) | 493.47 (415.05 - 579.27) | 50 (42 - 60) | 447.71 (371.36 - 529.38) | 0.33 (0.16 - 0.55) | -0.49 (-0.63 to -0.34) |
| Norway | 20,351 (17,517 - 24,049) | 2549.13 (2194.17 - 3012.29) | 24,015 (20,607 - 28,678) | 2599.56 (2230.67 - 3104.37) | 0.27 (0.22 - 0.33) | 0.58 (0.21 - 0.96) |
| Oman | 24,398 (20,161 - 28,920) | 2903.25 (2399.04 - 3441.34) | 36,014 (29,556 - 43,491) | 2944.72 (2416.65 - 3556.04) | 1.62 (1.35 - 1.96) | -0.02 (-0.09 - 0.06) |
| Pakistan | 1,883,946 (1,589,839 - 2,313,081) | 3825.89 (3228.62 - 4697.37) | 3,230,181 (2,717,551 - 3,834,325) | 3780.55 (3180.58 - 4487.63) | 1.52 (1.34 - 1.73) | 2.91 (2.03 - 3.79) |
| Palau | 17 (14 - 20) | 367.06 (307.95 - 428.82) | 12 (9 - 14) | 355.91 (287.51 - 434.12) | 0.52 (0.30 - 0.74) | -0.09 (-0.14 to -0.04) |
| Palestine | 28,169 (23,601 - 33,241) | 2909.17 (2437.36 - 3432.96) | 56,421 (46,887 - 68,155) | 3021.75 (2511.13 - 3650.19) | 1.75 (1.51 - 2.01) | 0.14 (0.12 - 0.17) |
| Panama | 53,992 (45,456 - 64,122) | 6474.58 (5451.04 - 7689.33) | 75,011 (61,174 - 90,166) | 6503.67 (5303.95 - 7817.66) | 0.92 (0.74 - 1.13) | -0.05 (-0.08 to -0.02) |
| Papua New Guinea | 4,694 (4,003 - 5,537) | 276.13 (235.46 - 325.71) | 10,838 (8,878 - 12,998) | 276.69 (226.66 - 331.82) | 1.92 (1.59 - 2.34) | 0.05 (0.02 - 0.09) |
| Paraguay | 75,189 (63,717 - 88,912) | 4503.46 (3816.37 - 5325.43) | 90,952 (76,868 - 106,123) | 4530.12 (3828.61 - 5285.71) | 1.01 (0.82 - 1.24) | 0.05 (0.04 - 0.07) |
| Peru | 368,349 (300,917 - 455,364) | 4437.45 (3625.12 - 5485.71) | 435,358 (358,164 - 540,972) | 4564.75 (3755.36 - 5672.12) | 1.01 (0.79 - 1.23) | 0.08 (0.06 - 0.10) |
| Philippines | 106,421 (91,324 - 126,384) | 422.06 (362.19 - 501.24) | 149,020 (127,798 - 177,906) | 438.31 (375.89 - 523.28) | 1.59 (1.45 - 1.74) | 0.07 (-0.01 - 0.15) |
| Poland | 571,045 (510,500 - 634,124) | 5962.79 (5330.58 - 6621.45) | 194,097 (172,146 - 217,961) | 3297.76 (2924.80 - 3703.22) | -0.17 (-0.21 to -0.11) | -3.33 (-4.11 to -2.55) |
| Portugal | 34,543 (28,440 - 42,091) | 1632.55 (1344.09 - 1989.29) | 22,648 (19,002 - 26,797) | 1662.56 (1394.89 - 1967.10) | -0.09 (-0.30 - 0.09) | 0.14 (0.08 - 0.20) |
| Puerto Rico | 56,801 (47,465 - 67,435) | 5704.25 (4766.70 - 6772.13) | 26,561 (21,578 - 32,551) | 5976.98 (4855.71 - 7324.83) | -0.05 (-0.14 - 0.06) | 0.17 (0.15 - 0.18) |
| Qatar | 4,639 (3,889 - 5,586) | 3709.77 (3109.88 - 4466.97) | 18,832 (15,946 - 22,950) | 3812.83 (3228.52 - 4646.70) | 6.09 (5.42 - 6.96) | -0.40 (-0.77 to -0.02) |
| Republic of Korea | 543,863 (453,746 - 639,606) | 4783.07 (3990.53 - 5625.10) | 292,475 (245,680 - 342,789) | 4814.80 (4044.46 - 5643.09) | 0.16 (0.03 - 0.31) | -0.00 (-0.07 - 0.06) |
| Republic of Moldova | 73,311 (61,537 - 85,976) | 5931.73 (4979.02 - 6956.41) | 30,404 (25,542 - 36,675) | 5821.00 (4890.10 - 7021.75) | -0.13 (-0.22 to -0.05) | 0.09 (0.02 - 0.15) |
| Romania | 204,838 (176,970 - 235,981) | 3678.82 (3178.31 - 4238.14) | 210,591 (183,444 - 246,932) | 6996.36 (6094.49 - 8203.71) | -0.01 (-0.10 - 0.08) | 0.61 (0.22 - 1.00) |
| Russian Federation | 2,658,851 (2,369,117 - 2,981,510) | 7662.72 (6827.71 - 8592.61) | 2,733,043 (2,423,142 - 3,044,455) | 10480.52 (9292.13 - 11674.70) | 0.10 (0.06 - 0.17) | 0.85 (0.52 - 1.18) |
| Rwanda | 22,489 (18,987 - 27,375) | 662.83 (559.62 - 806.83) | 34,405 (28,556 - 42,144) | 692.20 (574.52 - 847.89) | 1.20 (0.97 - 1.45) | 0.11 (0.06 - 0.15) |
| Saint Kitts and Nevis | 637 (538 - 771) | 4514.38 (3813.10 - 5458.87) | 454 (373 - 557) | 4608.63 (3788.88 - 5654.39) | 0.66 (0.45 - 0.88) | 0.09 (0.05 - 0.14) |
| Saint Lucia | 2,487 (2,090 - 2,958) | 4824.28 (4055.70 - 5738.11) | 1,505 (1,225 - 1,855) | 5067.30 (4125.38 - 6247.82) | 0.48 (0.32 - 0.71) | 0.16 (0.13 - 0.19) |
| Saint Vincent and the Grenadines | 1,838 (1,538 - 2,216) | 4472.46 (3744.17 - 5393.73) | 1,123 (915 - 1,392) | 4502.71 (3667.54 - 5578.03) | 0.19 (0.06 - 0.32) | 0.01 (-0.02 - 0.03) |
| Samoa | 276 (231 - 326) | 387.83 (324.81 - 457.25) | 310 (261 - 367) | 387.31 (326.57 - 459.02) | 0.51 (0.36 - 0.66) | 0.05 (0.02 - 0.07) |
| San Marino | 76 (63 - 91) | 1846.32 (1534.83 - 2219.80) | 80 (67 - 97) | 1810.51 (1515.64 - 2195.66) | 0.28 (0.12 - 0.47) | -0.01 (-0.10 - 0.08) |
| Sao Tome and Principe | 610 (510 - 717) | 1075.97 (900.73 - 1265.67) | 870 (745 - 1,034) | 1118.38 (957.67 - 1329.00) | 1.24 (1.01 - 1.53) | 0.02 (-0.03 - 0.07) |
| Saudi Arabia | 192,023 (157,699 - 228,330) | 2930.13 (2406.38 - 3484.16) | 228,990 (185,401 - 283,236) | 3026.85 (2450.69 - 3743.91) | 1.67 (1.40 - 1.98) | 0.08 (0.03 - 0.13) |
| Senegal | 37,463 (32,362 - 44,976) | 1026.03 (886.31 - 1231.78) | 65,807 (54,638 - 78,744) | 1034.61 (859.01 - 1238.00) | 1.37 (1.08 - 1.61) | 0.00 (-0.02 - 0.03) |
| Serbia | 77,445 (64,870 - 90,862) | 3570.79 (2991.00 - 4189.42) | 42,946 (36,981 - 50,426) | 3234.32 (2785.05 - 3797.61) | -0.03 (-0.12 - 0.04) | -0.22 (-0.32 to -0.12) |
| Seychelles | 118 (99 - 139) | 496.31 (419.19 - 584.77) | 122 (102 - 145) | 519.19 (436.11 - 620.32) | 0.91 (0.70 - 1.22) | 0.07 (0.04 - 0.11) |
| Sierra Leone | 16,175 (13,472 - 19,741) | 892.35 (743.23 - 1089.08) | 32,268 (26,882 - 38,135) | 902.30 (751.69 - 1066.35) | 1.29 (1.02 - 1.52) | 0.11 (0.07 - 0.14) |
| Singapore | 36,014 (30,072 - 41,232) | 5545.97 (4630.94 - 6349.52) | 46,808 (40,607 - 54,365) | 5763.86 (5000.25 - 6694.46) | 0.87 (0.70 - 1.09) | 0.10 (0.05 - 0.16) |
| Slovakia | 62,178 (53,373 - 72,097) | 4690.32 (4026.10 - 5438.47) | 41,350 (35,090 - 48,465) | 4827.46 (4096.70 - 5658.08) | 0.11 (0.01 - 0.22) | 0.11 (-0.41 - 0.62) |
| Slovenia | 19,873 (17,166 - 22,918) | 4805.80 (4151.17 - 5542.08) | 32,754 (28,341 - 37,156) | 10489.10 (9075.75 - 11898.96) | 0.29 (0.18 - 0.41) | 2.16 (1.55 - 2.76) |
| Solomon Islands | 509 (432 - 598) | 326.62 (277.63 - 384.18) | 838 (712 - 982) | 322.16 (273.90 - 377.53) | 1.61 (1.32 - 1.97) | -0.04 (-0.06 to -0.02) |
| Somalia | 24,383 (20,507 - 29,078) | 625.90 (526.42 - 746.43) | 63,857 (52,101 - 76,965) | 618.21 (504.39 - 745.10) | 1.78 (1.51 - 2.08) | -0.08 (-0.14 to -0.02) |
| South Africa | 242,998 (210,643 - 283,269) | 1784.98 (1547.31 - 2080.80) | 270,588 (232,617 - 318,872) | 1779.58 (1529.85 - 2097.13) | 0.69 (0.60 - 0.80) | -0.03 (-0.07 - 0.00) |
| South Sudan | 16,598 (14,085 - 19,850) | 632.49 (536.76 - 756.44) | 27,819 (23,007 - 33,813) | 647.72 (535.68 - 787.28) | 0.78 (0.60 - 0.99) | -0.01 (-0.06 - 0.03) |
| Spain | 145,549 (118,593 - 176,686) | 1857.43 (1513.44 - 2254.79) | 121,552 (98,851 - 146,835) | 1875.58 (1525.30 - 2265.70) | 0.17 (0.02 - 0.33) | -0.17 (-0.27 to -0.07) |
| Sri Lanka | 25,293 (21,300 - 30,889) | 457.12 (384.95 - 558.27) | 24,572 (20,619 - 29,598) | 481.43 (403.97 - 579.89) | 0.81 (0.61 - 1.02) | 0.08 (0.05 - 0.11) |
| Sudan | 195,359 (162,444 - 233,277) | 2196.91 (1826.77 - 2623.31) | 380,308 (309,550 - 464,063) | 2292.49 (1865.97 - 2797.37) | 1.36 (1.12 - 1.66) | 0.11 (0.08 - 0.14) |
| Suriname | 5,380 (4,389 - 6,588) | 4129.66 (3369.19 - 5057.05) | 5,893 (4,870 - 7,205) | 4113.41 (3398.85 - 5028.86) | 0.68 (0.52 - 0.86) | -0.02 (-0.05 - 0.01) |
| Sweden | 37,435 (31,237 - 44,559) | 2423.83 (2022.54 - 2885.15) | 48,136 (40,918 - 57,450) | 2643.77 (2247.32 - 3155.31) | 0.17 (0.06 - 0.29) | 0.21 (-0.05 - 0.47) |
| Switzerland | 18,754 (15,893 - 22,474) | 1622.86 (1375.24 - 1944.72) | 21,753 (18,131 - 26,109) | 1632.53 (1360.67 - 1959.42) | 0.23 (0.07 - 0.38) | 0.00 (-0.11 - 0.12) |
| Syrian Arab Republic | 169,683 (141,151 - 201,488) | 2865.24 (2383.46 - 3402.31) | 113,894 (94,011 - 137,784) | 3109.03 (2566.28 - 3761.19) | 0.28 (0.16 - 0.42) | 0.34 (0.27 - 0.41) |
| Taiwan (Province of China) | 11,891 (9,796 - 14,227) | 215.89 (177.85 - 258.29) | 6,205 (4,981 - 7,424) | 210.56 (169.04 - 251.95) | 0.63 (0.46 - 0.86) | -0.02 (-0.09 - 0.05) |
| Tajikistan | 75,804 (64,773 - 88,386) | 3264.54 (2789.50 - 3806.40) | 115,918 (98,391 - 139,930) | 3234.00 (2745.01 - 3903.91) | 1.13 (0.89 - 1.43) | 0.03 (0.01 - 0.06) |
| Thailand | 74,187 (61,511 - 89,826) | 440.06 (364.86 - 532.82) | 44,473 (35,935 - 54,844) | 455.37 (367.95 - 561.57) | 0.70 (0.48 - 0.93) | 0.09 (0.06 - 0.12) |
| Timor-Leste | 996 (842 - 1,197) | 299.43 (252.99 - 359.88) | 1,692 (1,408 - 2,082) | 325.06 (270.46 - 399.94) | 1.02 (0.80 - 1.27) | 0.27 (0.24 - 0.31) |
| Togo | 17,197 (14,554 - 20,293) | 975.67 (825.73 - 1151.38) | 32,501 (27,584 - 38,614) | 982.19 (833.60 - 1166.93) | 1.67 (1.38 - 2.07) | 0.01 (-0.03 - 0.04) |
| Tokelau | 2 (2 - 3) | 363.68 (309.17 - 422.57) | 1 (1 - 2) | 351.90 (294.36 - 424.70) | 0.05 (-0.09 - 0.20) | -0.05 (-0.14 - 0.04) |
| Tonga | 164 (140 - 191) | 391.85 (335.28 - 456.96) | 154 (130 - 182) | 395.24 (333.94 - 466.49) | 0.25 (0.13 - 0.37) | 0.02 (-0.01 - 0.04) |
| Trinidad and Tobago | 18,869 (15,254 - 22,780) | 4643.85 (3754.08 - 5606.35) | 12,877 (10,794 - 15,670) | 4726.96 (3962.08 - 5752.25) | 0.27 (0.12 - 0.41) | -0.03 (-0.06 - 0.01) |
| Tunisia | 93,562 (77,811 - 112,879) | 3012.79 (2505.59 - 3634.84) | 83,228 (66,473 - 102,271) | 3009.26 (2403.48 - 3697.79) | 0.51 (0.36 - 0.69) | -0.02 (-0.07 - 0.03) |
| Turkey | 580,634 (488,260 - 691,514) | 2833.92 (2383.07 - 3375.10) | 537,387 (442,672 - 656,911) | 2901.59 (2390.18 - 3546.95) | 0.57 (0.44 - 0.73) | 0.44 (0.21 - 0.66) |
| Turkmenistan | 50,586 (43,815 - 59,003) | 3370.48 (2919.34 - 3931.28) | 50,450 (42,842 - 58,974) | 3310.35 (2811.17 - 3869.67) | 0.63 (0.46 - 0.80) | 0.09 (0.06 - 0.13) |
| Tuvalu | 11 (10 - 14) | 328.46 (277.66 - 388.73) | 12 (10 - 14) | 316.67 (262.30 - 379.30) | 0.34 (0.15 - 0.51) | -0.07 (-0.12 to -0.02) |
| Uganda | 55,663 (47,318 - 64,963) | 661.11 (561.99 - 771.57) | 133,278 (112,605 - 162,266) | 671.91 (567.69 - 818.05) | 1.66 (1.43 - 1.97) | 0.05 (0.02 - 0.07) |
| Ukraine | 1,538,056 (1,309,168 - 1,820,494) | 13521.49 (11509.27 - 16004.49) | 773,017 (668,488 - 909,195) | 12182.70 (10535.33 - 14328.87) | -0.22 (-0.30 to -0.17) | 0.63 (-0.29 - 1.56) |
| United Arab Emirates | 19,402 (16,104 - 23,034) | 3291.97 (2732.25 - 3908.16) | 45,666 (38,150 - 54,922) | 3410.98 (2849.55 - 4102.35) | 4.12 (3.47 - 4.83) | 0.03 (0.00 - 0.07) |
| United Kingdom | 178,145 (150,685 - 211,046) | 1631.29 (1379.84 - 1932.57) | 136,735 (115,578 - 161,998) | 1160.44 (980.88 - 1374.84) | -0.11 (-0.14 to -0.09) | 0.16 (-0.61 - 0.94) |
| United Republic of Tanzania | 83,304 (70,087 - 98,264) | 689.86 (580.40 - 813.75) | 167,316 (137,723 - 201,645) | 685.64 (564.37 - 826.32) | 1.49 (1.24 - 1.76) | -0.01 (-0.03 - 0.00) |
| United States of America | 1,749,390 (1,520,342 - 2,020,714) | 3128.92 (2719.25 - 3614.21) | 1,655,175 (1,450,855 - 1,903,964) | 2784.88 (2441.11 - 3203.48) | 0.35 (0.29 - 0.40) | -1.11 (-1.56 to -0.64) |
| United States Virgin Islands | 1,668 (1,355 - 2,044) | 5221.11 (4239.94 - 6396.32) | 708 (576 - 856) | 5285.79 (4302.26 - 6395.31) | -0.17 (-0.27 to -0.05) | 0.05 (0.02 - 0.08) |
| Uruguay | 18,513 (15,748 - 22,232) | 2261.63 (1923.85 - 2715.96) | 15,287 (12,616 - 18,672) | 2317.95 (1912.85 - 2831.18) | 0.26 (0.11 - 0.41) | 0.03 (0.01 - 0.06) |
| Uzbekistan | 302,570 (260,786 - 352,744) | 3536.50 (3048.12 - 4122.94) | 353,014 (305,400 - 409,412) | 3498.28 (3026.44 - 4057.17) | 1.00 (0.74 - 1.43) | 0.02 (-0.02 - 0.06) |
| Vanuatu | 230 (194 - 270) | 337.49 (284.82 - 396.50) | 383 (324 - 461) | 328.90 (277.97 - 395.16) | 1.46 (1.17 - 1.80) | -0.09 (-0.11 to -0.07) |
| Venezuela (Bolivarian Republic of) | 468,788 (391,521 - 557,604) | 6608.28 (5519.09 - 7860.29) | 427,752 (359,169 - 519,008) | 6457.67 (5422.29 - 7835.35) | 0.57 (0.40 - 0.77) | -0.08 (-0.10 to -0.06) |
| Viet Nam | 129,614 (110,239 - 154,095) | 488.88 (415.80 - 581.22) | 143,066 (113,564 - 177,081) | 577.75 (458.61 - 715.11) | 0.96 (0.73 - 1.22) | 0.60 (0.52 - 0.69) |
| Yemen | 156,822 (128,737 - 190,726) | 2210.51 (1814.63 - 2688.41) | 320,382 (260,313 - 392,618) | 2323.54 (1887.90 - 2847.42) | 1.77 (1.47 - 2.11) | 0.11 (0.08 - 0.15) |
| Zambia | 25,416 (21,483 - 30,871) | 676.94 (572.18 - 822.24) | 56,808 (46,426 - 69,102) | 686.78 (561.28 - 835.42) | 1.72 (1.47 - 2.01) | -0.01 (-0.03 - 0.01) |
| Zimbabwe | 77,497 (65,725 - 92,322) | 1609.05 (1364.63 - 1916.87) | 100,882 (85,534 - 119,943) | 1602.86 (1359.01 - 1905.72) | 0.67 (0.50 - 0.84) | -0.10 (-0.13 to -0.06) |

**Table S2.** The death cases and ASDR of Pediatric Urinary Tract Infection in 1990 and 2021, with Temporal Trends from 1990 to 2021 in 204 countries or territories.

| **location** | **Num_1990** | **ASDR_1990** | **Num_2021** | **ASDR_2021** | **Num_change** | **EAPC_ASDR** |
| --- | --- | --- | --- | --- | --- | --- |
| Afghanistan | 19 (12 - 28) | 0.45 (0.28 - 0.64) | 37 (22 - 54) | 0.26 (0.15 - 0.38) | 0.61 (0.15 - 1.36) | -1.61 (-1.77 to -1.46) |
| Albania | 1 (0 - 1) | 0.06 (0.03 - 0.11) | 0 (0 - 0) | 0.01 (0.01 - 0.02) | 0.19 (-0.30 - 1.16) | -5.36 (-5.70 to -5.02) |
| Algeria | 22 (15 - 30) | 0.21 (0.14 - 0.28) | 13 (9 - 17) | 0.09 (0.07 - 0.13) | 2.42 (1.09 - 4.16) | -2.48 (-2.85 to -2.11) |
| American Samoa | 0 (0 - 0) | 0.23 (0.17 - 0.30) | 0 (0 - 0) | 0.10 (0.07 - 0.14) | 1.10 (0.59 - 1.65) | -2.19 (-2.40 to -1.98) |
| Andorra | 0 (0 - 0) | 0.06 (0.04 - 0.09) | 0 (0 - 0) | 0.01 (0.01 - 0.02) | 1.73 (0.74 - 3.32) | -4.23 (-4.44 to -4.01) |
| Angola | 21 (8 - 34) | 0.45 (0.17 - 0.71) | 23 (14 - 35) | 0.15 (0.09 - 0.23) | 1.43 (0.68 - 2.43) | -2.43 (-2.78 to -2.07) |
| Antigua and Barbuda | 0 (0 - 0) | 0.04 (0.04 - 0.05) | 0 (0 - 0) | 0.13 (0.11 - 0.15) | 4.45 (3.87 - 5.04) | 3.62 (2.99 - 4.25) |
| Argentina | 5 (5 - 5) | 0.05 (0.04 - 0.05) | 11 (10 - 13) | 0.11 (0.09 - 0.13) | 15.48 (13.91 - 17.18) | 3.88 (3.18 - 4.58) |
| Armenia | 3 (2 - 3) | 0.24 (0.20 - 0.31) | 1 (1 - 1) | 0.13 (0.10 - 0.16) | 0.90 (0.42 - 1.60) | -0.68 (-1.39 - 0.03) |
| Australia | 2 (2 - 3) | 0.06 (0.06 - 0.07) | 2 (1 - 2) | 0.03 (0.03 - 0.04) | 3.54 (2.94 - 4.04) | -1.75 (-2.14 to -1.36) |
| Austria | 0 (0 - 0) | 0.01 (0.01 - 0.01) | 0 (0 - 0) | 0.01 (0.01 - 0.01) | 0.95 (0.69 - 1.20) | -0.82 (-1.51 to -0.13) |
| Azerbaijan | 8 (6 - 10) | 0.33 (0.26 - 0.41) | 6 (4 - 7) | 0.24 (0.18 - 0.30) | 1.47 (0.60 - 2.73) | -0.82 (-1.10 to -0.54) |
| Bahamas | 0 (0 - 0) | 0.09 (0.07 - 0.11) | 0 (0 - 0) | 0.16 (0.12 - 0.21) | 7.93 (6.08 - 10.23) | 1.88 (1.34 - 2.42) |
| Bahrain | 0 (0 - 0) | 0.02 (0.01 - 0.03) | 0 (0 - 0) | 0.01 (0.01 - 0.02) | 4.12 (2.14 - 10.47) | -0.25 (-1.09 - 0.59) |
| Bangladesh | 763 (419 - 1,018) | 1.56 (0.86 - 2.08) | 214 (163 - 279) | 0.47 (0.36 - 0.61) | 0.63 (0.22 - 1.13) | -3.55 (-3.66 to -3.45) |
| Barbados | 0 (0 - 0) | 0.16 (0.13 - 0.19) | 0 (0 - 0) | 0.26 (0.19 - 0.35) | 4.39 (3.28 - 5.55) | 2.21 (1.66 - 2.76) |
| Belarus | 3 (2 - 3) | 0.12 (0.10 - 0.14) | 1 (1 - 1) | 0.06 (0.04 - 0.07) | 0.84 (0.49 - 1.25) | -2.18 (-2.65 to -1.70) |
| Belgium | 0 (0 - 0) | 0.02 (0.02 - 0.02) | 0 (0 - 0) | 0.02 (0.02 - 0.02) | 7.21 (6.17 - 8.24) | -0.48 (-1.02 - 0.07) |
| Belize | 0 (0 - 0) | 0.15 (0.13 - 0.18) | 0 (0 - 0) | 0.18 (0.15 - 0.22) | 9.28 (7.73 - 10.83) | 0.57 (-0.00 - 1.15) |
| Benin | 30 (16 - 43) | 1.24 (0.66 - 1.78) | 35 (22 - 50) | 0.57 (0.35 - 0.83) | 0.72 (0.26 - 1.46) | -2.00 (-2.12 to -1.87) |
| Bermuda | 0 (0 - 0) | 0.01 (0.01 - 0.02) | 0 (0 - 0) | 0.03 (0.02 - 0.04) | 5.56 (4.51 - 6.97) | 2.17 (1.61 - 2.72) |
| Bhutan | 3 (2 - 5) | 1.21 (0.61 - 1.88) | 1 (1 - 2) | 0.63 (0.40 - 0.96) | 1.45 (0.74 - 2.43) | -1.67 (-1.96 to -1.39) |
| Bolivia (Plurinational State of) | 27 (21 - 35) | 1.01 (0.79 - 1.30) | 14 (9 - 19) | 0.39 (0.26 - 0.56) | 2.09 (1.04 - 3.36) | -2.83 (-2.90 to -2.75) |
| Bosnia and Herzegovina | 0 (0 - 0) | 0.03 (0.02 - 0.04) | 0 (0 - 0) | 0.01 (0.01 - 0.02) | 0.01 (-0.38 - 0.74) | -3.77 (-4.26 to -3.28) |
| Botswana | 0 (0 - 0) | 0.03 (0.02 - 0.05) | 0 (0 - 0) | 0.05 (0.02 - 0.07) | 1.74 (0.86 - 3.14) | 1.09 (0.85 - 1.34) |
| Brazil | 304 (268 - 337) | 0.58 (0.52 - 0.65) | 187 (149 - 226) | 0.39 (0.31 - 0.47) | 8.33 (7.45 - 8.96) | -1.51 (-1.95 to -1.07) |
| Brunei Darussalam | 0 (0 - 0) | 0.10 (0.07 - 0.16) | 0 (0 - 0) | 0.10 (0.07 - 0.14) | 2.00 (0.66 - 3.43) | -0.40 (-0.88 - 0.08) |
| Bulgaria | 3 (3 - 4) | 0.20 (0.17 - 0.22) | 1 (1 - 1) | 0.07 (0.06 - 0.09) | -0.56 (-0.65 to -0.46) | -4.13 (-4.88 to -3.37) |
| Burkina Faso | 49 (22 - 74) | 1.04 (0.48 - 1.56) | 62 (33 - 90) | 0.60 (0.32 - 0.87) | 0.64 (0.13 - 1.30) | -1.60 (-1.71 to -1.50) |
| Burundi | 31 (15 - 51) | 1.19 (0.58 - 1.96) | 20 (11 - 35) | 0.34 (0.20 - 0.59) | 0.39 (-0.03 - 0.90) | -2.95 (-3.19 to -2.72) |
| Cabo Verde | 0 (0 - 1) | 0.23 (0.11 - 0.33) | 0 (0 - 0) | 0.06 (0.04 - 0.10) | 1.05 (0.30 - 2.05) | -3.61 (-3.89 to -3.34) |
| Cambodia | 20 (12 - 28) | 0.44 (0.26 - 0.59) | 9 (7 - 11) | 0.17 (0.13 - 0.22) | 1.40 (0.66 - 2.27) | -2.83 (-2.99 to -2.67) |
| Cameroon | 56 (33 - 78) | 1.16 (0.67 - 1.61) | 67 (42 - 96) | 0.50 (0.31 - 0.72) | 0.86 (0.30 - 1.74) | -1.98 (-2.12 to -1.84) |
| Canada | 2 (2 - 2) | 0.03 (0.03 - 0.03) | 1 (1 - 1) | 0.02 (0.01 - 0.02) | 2.80 (2.39 - 3.25) | -1.20 (-1.43 to -0.98) |
| Central African Republic | 5 (3 - 8) | 0.44 (0.21 - 0.67) | 6 (4 - 9) | 0.26 (0.16 - 0.38) | 0.66 (0.16 - 1.25) | -1.12 (-1.23 to -1.01) |
| Chad | 26 (11 - 38) | 0.87 (0.36 - 1.29) | 48 (20 - 75) | 0.53 (0.22 - 0.83) | 0.96 (0.42 - 1.69) | -1.53 (-1.57 to -1.49) |
| Chile | 3 (2 - 3) | 0.07 (0.06 - 0.07) | 3 (3 - 4) | 0.09 (0.08 - 0.11) | 3.68 (3.22 - 4.09) | 2.07 (1.56 - 2.59) |
| China | 378 (229 - 474) | 0.12 (0.07 - 0.15) | 41 (33 - 54) | 0.02 (0.01 - 0.02) | 0.30 (-0.23 - 1.34) | -5.48 (-5.95 to -5.01) |
| Colombia | 38 (33 - 45) | 0.33 (0.28 - 0.38) | 25 (19 - 34) | 0.24 (0.18 - 0.32) | 5.22 (4.26 - 6.23) | -1.63 (-2.18 to -1.08) |
| Comoros | 2 (1 - 3) | 1.01 (0.56 - 1.59) | 1 (1 - 2) | 0.54 (0.37 - 0.80) | 1.18 (0.50 - 2.09) | -1.31 (-1.60 to -1.01) |
| Congo | 3 (2 - 5) | 0.29 (0.15 - 0.44) | 2 (1 - 3) | 0.10 (0.07 - 0.15) | 0.99 (0.47 - 1.74) | -2.51 (-2.88 to -2.13) |
| Cook Islands | 0 (0 - 0) | 0.01 (0.00 - 0.01) | 0 (0 - 0) | 0.00 (0.00 - 0.01) | 0.64 (0.22 - 1.13) | -5.79 (-6.46 to -5.12) |
| Costa Rica | 1 (1 - 1) | 0.10 (0.09 - 0.12) | 1 (1 - 1) | 0.11 (0.09 - 0.13) | 7.34 (6.34 - 8.44) | -1.15 (-1.82 to -0.47) |
| Côte d'Ivoire | 47 (28 - 65) | 0.82 (0.50 - 1.14) | 46 (30 - 64) | 0.40 (0.26 - 0.55) | 0.85 (0.30 - 1.64) | -1.73 (-1.90 to -1.55) |
| Croatia | 1 (1 - 1) | 0.11 (0.10 - 0.12) | 0 (0 - 0) | 0.04 (0.03 - 0.05) | 1.17 (0.82 - 1.59) | -4.92 (-5.99 to -3.85) |
| Cuba | 0 (0 - 0) | 0.02 (0.01 - 0.02) | 1 (0 - 1) | 0.03 (0.02 - 0.03) | 6.62 (5.40 - 7.80) | 2.66 (2.24 - 3.08) |
| Cyprus | 0 (0 - 0) | 0.10 (0.07 - 0.13) | 0 (0 - 0) | 0.02 (0.02 - 0.03) | 0.82 (0.29 - 1.53) | -4.47 (-4.77 to -4.18) |
| Czechia | 3 (3 - 3) | 0.13 (0.12 - 0.14) | 1 (1 - 1) | 0.04 (0.03 - 0.05) | -0.11 (-0.30 - 0.11) | -4.99 (-5.86 to -4.11) |
| Democratic People's Republic of Korea | 6 (4 - 9) | 0.10 (0.06 - 0.15) | 2 (1 - 3) | 0.03 (0.02 - 0.07) | 0.71 (0.15 - 1.45) | -2.64 (-2.84 to -2.44) |
| Democratic Republic of the Congo | 65 (30 - 97) | 0.37 (0.17 - 0.55) | 40 (26 - 58) | 0.11 (0.07 - 0.15) | 0.91 (0.32 - 1.72) | -2.86 (-3.12 to -2.61) |
| Denmark | 0 (0 - 0) | 0.03 (0.02 - 0.03) | 0 (0 - 0) | 0.02 (0.02 - 0.02) | 2.87 (2.40 - 3.38) | -1.19 (-1.51 to -0.88) |
| Djibouti | 1 (1 - 2) | 0.65 (0.36 - 1.01) | 1 (1 - 2) | 0.35 (0.21 - 0.54) | 3.85 (2.10 - 6.02) | -0.80 (-1.22 to -0.38) |
| Dominica | 0 (0 - 0) | 0.03 (0.02 - 0.04) | 0 (0 - 0) | 0.09 (0.05 - 0.13) | 1.85 (0.47 - 3.00) | 3.92 (3.65 - 4.19) |
| Dominican Republic | 3 (2 - 4) | 0.10 (0.07 - 0.14) | 1 (1 - 2) | 0.04 (0.02 - 0.07) | 2.34 (1.27 - 3.62) | -2.76 (-2.96 to -2.56) |
| Ecuador | 4 (4 - 5) | 0.11 (0.10 - 0.13) | 9 (6 - 11) | 0.17 (0.13 - 0.23) | 7.48 (5.67 - 9.42) | -0.19 (-1.05 - 0.67) |
| Egypt | 5 (3 - 9) | 0.02 (0.01 - 0.04) | 6 (4 - 9) | 0.02 (0.01 - 0.02) | 4.73 (1.39 - 8.25) | -0.65 (-1.26 to -0.03) |
| El Salvador | 14 (11 - 18) | 0.65 (0.50 - 0.81) | 4 (3 - 5) | 0.20 (0.14 - 0.27) | 1.45 (0.63 - 2.23) | -4.18 (-4.53 to -3.83) |
| Equatorial Guinea | 1 (0 - 1) | 0.34 (0.16 - 0.52) | 1 (0 - 1) | 0.13 (0.08 - 0.22) | 1.79 (0.70 - 3.40) | -2.58 (-2.80 to -2.35) |
| Eritrea | 12 (7 - 19) | 0.76 (0.42 - 1.19) | 10 (6 - 17) | 0.40 (0.24 - 0.67) | 1.17 (0.56 - 1.96) | -1.96 (-2.06 to -1.85) |
| Estonia | 1 (1 - 1) | 0.18 (0.16 - 0.19) | 0 (0 - 0) | 0.04 (0.04 - 0.05) | 0.08 (-0.10 - 0.28) | -3.69 (-4.32 to -3.04) |
| Eswatini | 0 (0 - 0) | 0.06 (0.03 - 0.09) | 0 (0 - 0) | 0.05 (0.03 - 0.08) | 1.25 (0.42 - 2.61) | -0.47 (-0.64 to -0.29) |
| Ethiopia | 494 (261 - 727) | 2.03 (1.07 - 2.98) | 270 (186 - 369) | 0.61 (0.42 - 0.83) | 0.22 (-0.06 - 0.58) | -3.89 (-4.06 to -3.73) |
| Fiji | 0 (0 - 0) | 0.04 (0.03 - 0.06) | 0 (0 - 0) | 0.06 (0.04 - 0.10) | 2.02 (0.90 - 3.57) | 1.81 (1.36 - 2.26) |
| Finland | 0 (0 - 0) | 0.02 (0.02 - 0.02) | 0 (0 - 0) | 0.02 (0.02 - 0.02) | 0.10 (-0.03 - 0.24) | -0.39 (-0.90 - 0.12) |
| France | 2 (2 - 2) | 0.02 (0.01 - 0.02) | 2 (2 - 2) | 0.02 (0.01 - 0.02) | 1.30 (1.02 - 1.57) | -0.49 (-1.01 - 0.03) |
| Gabon | 1 (0 - 1) | 0.21 (0.12 - 0.31) | 1 (0 - 1) | 0.12 (0.07 - 0.18) | 0.89 (0.12 - 1.76) | -1.03 (-1.27 to -0.79) |
| Gambia | 3 (2 - 5) | 0.69 (0.39 - 1.00) | 3 (2 - 4) | 0.27 (0.17 - 0.38) | 1.33 (0.57 - 2.57) | -2.31 (-2.63 to -1.98) |
| Georgia | 1 (1 - 1) | 0.05 (0.04 - 0.05) | 0 (0 - 0) | 0.02 (0.01 - 0.02) | 1.26 (0.79 - 1.87) | -0.87 (-1.56 to -0.17) |
| Germany | 2 (2 - 2) | 0.01 (0.01 - 0.02) | 2 (2 - 2) | 0.01 (0.01 - 0.02) | 3.88 (3.18 - 4.53) | -0.76 (-1.35 to -0.17) |
| Ghana | 42 (22 - 63) | 0.63 (0.33 - 0.94) | 46 (27 - 84) | 0.36 (0.21 - 0.66) | 2.54 (1.48 - 4.26) | -1.41 (-1.58 to -1.24) |
| Greece | 0 (0 - 0) | 0.01 (0.01 - 0.01) | 0 (0 - 0) | 0.01 (0.01 - 0.02) | 2.92 (2.45 - 3.39) | -0.20 (-0.85 - 0.46) |
| Greenland | 0 (0 - 0) | 0.11 (0.07 - 0.17) | 0 (0 - 0) | 0.04 (0.03 - 0.07) | 0.57 (0.17 - 1.00) | -3.05 (-3.22 to -2.87) |
| Grenada | 0 (0 - 0) | 0.06 (0.05 - 0.08) | 0 (0 - 0) | 0.10 (0.08 - 0.13) | 2.04 (1.24 - 2.82) | 1.61 (1.23 - 2.00) |
| Guam | 0 (0 - 0) | 0.12 (0.09 - 0.16) | 0 (0 - 0) | 0.11 (0.08 - 0.14) | 0.75 (0.37 - 1.34) | -0.04 (-0.30 - 0.23) |
| Guatemala | 15 (13 - 17) | 0.37 (0.32 - 0.42) | 15 (11 - 19) | 0.29 (0.23 - 0.38) | 5.65 (4.62 - 6.76) | -1.67 (-2.36 to -0.99) |
| Guinea | 40 (18 - 59) | 1.46 (0.64 - 2.13) | 29 (17 - 42) | 0.49 (0.29 - 0.69) | 0.26 (-0.09 - 0.87) | -3.06 (-3.18 to -2.94) |
| Guinea-Bissau | 6 (3 - 9) | 1.29 (0.65 - 1.94) | 3 (2 - 4) | 0.32 (0.21 - 0.46) | 0.10 (-0.25 - 0.68) | -3.41 (-3.69 to -3.13) |
| Guyana | 0 (0 - 0) | 0.13 (0.10 - 0.15) | 0 (0 - 1) | 0.22 (0.16 - 0.29) | 3.64 (2.51 - 4.87) | 2.83 (2.40 - 3.27) |
| Haiti | 18 (7 - 25) | 0.65 (0.28 - 0.92) | 21 (10 - 35) | 0.49 (0.22 - 0.80) | 1.45 (0.46 - 2.66) | -1.04 (-1.28 to -0.80) |
| Honduras | 19 (15 - 24) | 0.86 (0.68 - 1.09) | 7 (4 - 15) | 0.22 (0.12 - 0.44) | 2.91 (1.29 - 4.37) | -4.17 (-4.21 to -4.13) |
| Hungary | 2 (2 - 2) | 0.10 (0.09 - 0.11) | 1 (0 - 1) | 0.04 (0.03 - 0.05) | 0.08 (-0.10 - 0.30) | -4.21 (-5.20 to -3.20) |
| Iceland | 0 (0 - 0) | 0.01 (0.00 - 0.01) | 0 (0 - 0) | 0.01 (0.01 - 0.01) | 4.99 (4.21 - 5.80) | 0.55 (-0.31 - 1.42) |
| India | 3,337 (2,250 - 4,296) | 1.02 (0.69 - 1.32) | 1,755 (1,039 - 2,710) | 0.48 (0.28 - 0.74) | 1.85 (1.19 - 2.44) | -2.20 (-2.29 to -2.11) |
| Indonesia | 98 (63 - 128) | 0.15 (0.09 - 0.19) | 56 (44 - 69) | 0.08 (0.07 - 0.10) | 1.98 (1.25 - 2.81) | -1.46 (-1.55 to -1.37) |
| Iran (Islamic Republic of) | 105 (58 - 136) | 0.41 (0.23 - 0.53) | 11 (9 - 13) | 0.05 (0.04 - 0.06) | 1.33 (0.32 - 2.51) | -4.60 (-5.14 to -4.05) |
| Iraq | 5 (3 - 8) | 0.06 (0.04 - 0.10) | 2 (2 - 3) | 0.02 (0.01 - 0.02) | 1.14 (0.13 - 2.13) | -2.83 (-3.06 to -2.60) |
| Ireland | 0 (0 - 0) | 0.02 (0.02 - 0.03) | 0 (0 - 0) | 0.02 (0.01 - 0.02) | 1.39 (1.07 - 1.69) | -1.24 (-1.88 to -0.59) |
| Israel | 1 (1 - 1) | 0.05 (0.05 - 0.06) | 1 (1 - 1) | 0.03 (0.03 - 0.04) | 7.79 (6.62 - 8.80) | -1.67 (-2.19 to -1.14) |
| Italy | 2 (1 - 2) | 0.02 (0.02 - 0.02) | 1 (1 - 1) | 0.01 (0.01 - 0.02) | 3.75 (3.12 - 4.25) | -3.03 (-4.07 to -1.97) |
| Jamaica | 1 (1 - 1) | 0.10 (0.08 - 0.12) | 1 (1 - 1) | 0.12 (0.09 - 0.16) | 5.33 (3.92 - 7.02) | 0.58 (0.12 - 1.04) |
| Japan | 5 (5 - 5) | 0.02 (0.02 - 0.02) | 4 (3 - 4) | 0.02 (0.02 - 0.03) | 5.97 (5.00 - 6.60) | -0.29 (-0.91 - 0.33) |
| Jordan | 1 (1 - 1) | 0.07 (0.05 - 0.09) | 1 (1 - 2) | 0.04 (0.03 - 0.05) | 3.84 (2.16 - 6.25) | -1.80 (-1.93 to -1.67) |
| Kazakhstan | 11 (9 - 13) | 0.21 (0.18 - 0.25) | 12 (10 - 15) | 0.22 (0.18 - 0.27) | 0.69 (0.29 - 1.19) | 0.40 (0.11 - 0.70) |
| Kenya | 63 (40 - 85) | 0.56 (0.36 - 0.76) | 48 (34 - 80) | 0.26 (0.18 - 0.43) | 2.45 (1.68 - 3.45) | -2.22 (-2.53 to -1.91) |
| Kiribati | 0 (0 - 0) | 0.40 (0.26 - 0.57) | 0 (0 - 0) | 0.16 (0.10 - 0.28) | 0.75 (0.32 - 1.32) | -2.37 (-2.55 to -2.18) |
| Kuwait | 0 (0 - 0) | 0.01 (0.01 - 0.01) | 1 (1 - 1) | 0.09 (0.08 - 0.11) | 63.22 (52.58 - 74.78) | 4.56 (2.74 - 6.42) |
| Kyrgyzstan | 5 (4 - 6) | 0.30 (0.26 - 0.34) | 3 (3 - 4) | 0.15 (0.13 - 0.17) | 0.45 (0.15 - 0.84) | -0.85 (-1.41 to -0.30) |
| Lao People's Democratic Republic | 10 (5 - 14) | 0.56 (0.30 - 0.78) | 5 (4 - 7) | 0.22 (0.15 - 0.30) | 0.65 (0.21 - 1.33) | -2.80 (-2.93 to -2.67) |
| Latvia | 1 (1 - 1) | 0.19 (0.17 - 0.21) | 0 (0 - 0) | 0.04 (0.04 - 0.05) | 0.08 (-0.14 - 0.37) | -3.40 (-4.00 to -2.80) |
| Lebanon | 3 (2 - 4) | 0.28 (0.20 - 0.37) | 1 (1 - 2) | 0.09 (0.06 - 0.12) | 2.55 (1.75 - 3.61) | -3.00 (-3.25 to -2.74) |
| Lesotho | 0 (0 - 0) | 0.03 (0.02 - 0.05) | 0 (0 - 0) | 0.05 (0.03 - 0.08) | 1.46 (0.58 - 2.81) | 1.18 (0.77 - 1.59) |
| Liberia | 21 (10 - 31) | 1.89 (0.90 - 2.72) | 8 (5 - 11) | 0.36 (0.21 - 0.52) | 0.02 (-0.33 - 0.57) | -4.15 (-4.61 to -3.69) |
| Libya | 2 (1 - 3) | 0.11 (0.08 - 0.15) | 2 (1 - 3) | 0.12 (0.07 - 0.19) | 2.55 (0.60 - 5.26) | 0.35 (0.09 - 0.61) |
| Lithuania | 1 (1 - 1) | 0.10 (0.10 - 0.11) | 0 (0 - 0) | 0.04 (0.03 - 0.06) | 0.49 (0.22 - 0.79) | -2.80 (-3.41 to -2.18) |
| Luxembourg | 0 (0 - 0) | 0.01 (0.01 - 0.01) | 0 (0 - 0) | 0.01 (0.00 - 0.01) | 4.38 (3.65 - 5.15) | -2.01 (-2.54 to -1.47) |
| Madagascar | 45 (24 - 68) | 0.82 (0.43 - 1.25) | 34 (23 - 51) | 0.29 (0.20 - 0.44) | 0.66 (0.21 - 1.31) | -2.84 (-2.96 to -2.73) |
| Malawi | 40 (19 - 59) | 0.87 (0.42 - 1.29) | 21 (14 - 31) | 0.25 (0.18 - 0.38) | 0.79 (0.36 - 1.39) | -3.24 (-3.44 to -3.05) |
| Malaysia | 6 (4 - 8) | 0.09 (0.06 - 0.12) | 4 (3 - 5) | 0.05 (0.04 - 0.07) | 2.47 (1.23 - 7.00) | -1.85 (-2.15 to -1.54) |
| Maldives | 0 (0 - 0) | 0.35 (0.23 - 0.46) | 0 (0 - 0) | 0.10 (0.08 - 0.14) | 1.32 (0.63 - 2.06) | -4.05 (-4.35 to -3.74) |
| Mali | 54 (21 - 81) | 1.30 (0.50 - 1.96) | 56 (28 - 82) | 0.48 (0.24 - 0.71) | 0.50 (0.07 - 1.19) | -2.72 (-2.82 to -2.62) |
| Malta | 0 (0 - 0) | 0.03 (0.02 - 0.03) | 0 (0 - 0) | 0.04 (0.03 - 0.05) | 4.46 (3.71 - 5.25) | 0.13 (-0.55 - 0.81) |
| Marshall Islands | 0 (0 - 0) | 0.08 (0.05 - 0.12) | 0 (0 - 0) | 0.06 (0.04 - 0.10) | 1.02 (0.46 - 1.67) | -0.35 (-0.75 - 0.05) |
| Mauritania | 6 (4 - 8) | 0.62 (0.40 - 0.87) | 4 (3 - 6) | 0.22 (0.15 - 0.32) | 0.40 (-0.11 - 1.11) | -2.45 (-2.75 to -2.15) |
| Mauritius | 0 (0 - 0) | 0.02 (0.02 - 0.02) | 0 (0 - 0) | 0.07 (0.05 - 0.08) | 14.79 (13.32 - 16.34) | 2.95 (2.30 - 3.61) |
| Mexico | 147 (134 - 163) | 0.44 (0.40 - 0.49) | 81 (63 - 105) | 0.25 (0.20 - 0.33) | 5.21 (4.48 - 6.05) | -2.73 (-3.32 to -2.13) |
| Micronesia (Federated States of) | 0 (0 - 0) | 0.14 (0.10 - 0.19) | 0 (0 - 0) | 0.06 (0.04 - 0.09) | 0.26 (-0.11 - 0.77) | -2.83 (-2.96 to -2.69) |
| Monaco | 0 (0 - 0) | 0.01 (0.00 - 0.01) | 0 (0 - 0) | 0.00 (0.00 - 0.01) | 1.50 (0.49 - 2.65) | -1.96 (-2.29 to -1.62) |
| Mongolia | 3 (2 - 4) | 0.29 (0.21 - 0.40) | 1 (1 - 1) | 0.10 (0.07 - 0.13) | 0.37 (-0.02 - 0.86) | -3.08 (-3.41 to -2.74) |
| Montenegro | 0 (0 - 0) | 0.01 (0.00 - 0.01) | 0 (0 - 0) | 0.00 (0.00 - 0.00) | 0.76 (0.23 - 1.41) | -4.44 (-4.71 to -4.17) |
| Morocco | 26 (16 - 37) | 0.27 (0.16 - 0.38) | 10 (6 - 13) | 0.10 (0.06 - 0.14) | 2.04 (0.93 - 3.70) | -2.17 (-2.47 to -1.87) |
| Mozambique | 114 (58 - 189) | 1.83 (0.93 - 3.05) | 80 (51 - 135) | 0.56 (0.36 - 0.95) | 1.06 (0.41 - 1.84) | -3.13 (-3.29 to -2.96) |
| Myanmar | 84 (40 - 118) | 0.57 (0.27 - 0.80) | 42 (30 - 56) | 0.27 (0.19 - 0.36) | 0.73 (0.23 - 1.40) | -2.04 (-2.25 to -1.83) |
| Namibia | 0 (0 - 0) | 0.03 (0.02 - 0.05) | 0 (0 - 0) | 0.03 (0.01 - 0.04) | 1.42 (0.54 - 2.62) | -0.52 (-0.81 to -0.22) |
| Nauru | 0 (0 - 0) | 0.14 (0.09 - 0.20) | 0 (0 - 0) | 0.10 (0.07 - 0.16) | 0.22 (-0.08 - 0.70) | -0.42 (-0.84 - -0.00) |
| Nepal | 102 (62 - 138) | 1.21 (0.73 - 1.64) | 40 (28 - 58) | 0.43 (0.30 - 0.62) | 1.59 (0.92 - 2.45) | -3.40 (-3.58 to -3.23) |
| Netherlands | 1 (1 - 1) | 0.04 (0.04 - 0.04) | 1 (1 - 1) | 0.04 (0.04 - 0.05) | 1.59 (1.36 - 1.82) | -0.39 (-0.84 - 0.05) |
| New Zealand | 1 (1 - 1) | 0.14 (0.12 - 0.15) | 0 (0 - 1) | 0.05 (0.04 - 0.06) | 1.00 (0.71 - 1.32) | -2.44 (-3.23 to -1.64) |
| Nicaragua | 5 (4 - 7) | 0.28 (0.21 - 0.36) | 2 (1 - 2) | 0.08 (0.06 - 0.13) | 2.58 (1.39 - 3.77) | -3.63 (-3.89 to -3.36) |
| Niger | 60 (25 - 90) | 1.49 (0.62 - 2.22) | 48 (28 - 71) | 0.38 (0.22 - 0.56) | 0.37 (-0.12 - 1.24) | -3.89 (-4.17 to -3.61) |
| Nigeria | 363 (178 - 486) | 0.93 (0.45 - 1.24) | 508 (282 - 728) | 0.50 (0.28 - 0.72) | 0.69 (0.33 - 1.23) | -1.47 (-1.58 to -1.37) |
| Niue | 0 (0 - 0) | 0.08 (0.05 - 0.11) | 0 (0 - 0) | 0.28 (0.22 - 0.37) | -0.16 (-0.40 - 0.12) | 1.07 (0.49 - 1.65) |
| North Macedonia | 0 (0 - 0) | 0.02 (0.01 - 0.02) | 0 (0 - 0) | 0.00 (0.00 - 0.00) | 0.36 (-0.01 - 0.88) | -6.42 (-6.99 to -5.84) |
| Northern Mariana Islands | 0 (0 - 0) | 0.13 (0.09 - 0.19) | 0 (0 - 0) | 0.05 (0.04 - 0.07) | 1.68 (1.05 - 2.51) | -1.82 (-2.26 to -1.37) |
| Norway | 0 (0 - 0) | 0.02 (0.01 - 0.02) | 0 (0 - 0) | 0.01 (0.00 - 0.01) | 1.16 (1.01 - 1.30) | -2.02 (-2.36 to -1.68) |
| Oman | 2 (1 - 3) | 0.23 (0.15 - 0.31) | 1 (1 - 2) | 0.10 (0.08 - 0.13) | 1.76 (0.80 - 3.16) | -2.65 (-3.23 to -2.06) |
| Pakistan | 695 (507 - 932) | 1.41 (1.03 - 1.89) | 1,048 (781 - 1,424) | 1.23 (0.91 - 1.67) | 1.68 (1.10 - 2.33) | 0.05 (-0.17 - 0.28) |
| Palau | 0 (0 - 0) | 0.13 (0.08 - 0.21) | 0 (0 - 0) | 0.07 (0.05 - 0.09) | 0.84 (0.36 - 1.46) | -1.56 (-1.78 to -1.35) |
| Palestine | 1 (0 - 1) | 0.06 (0.03 - 0.10) | 0 (0 - 1) | 0.02 (0.02 - 0.04) | 1.26 (0.47 - 2.31) | -2.48 (-2.68 to -2.29) |
| Panama | 1 (1 - 1) | 0.15 (0.13 - 0.17) | 2 (2 - 3) | 0.20 (0.15 - 0.25) | 6.11 (4.74 - 7.62) | 0.25 (-0.21 - 0.72) |
| Papua New Guinea | 3 (2 - 6) | 0.20 (0.09 - 0.33) | 6 (3 - 10) | 0.16 (0.09 - 0.25) | 1.50 (0.79 - 2.56) | -0.41 (-0.52 to -0.30) |
| Paraguay | 2 (1 - 3) | 0.13 (0.08 - 0.19) | 2 (2 - 4) | 0.12 (0.08 - 0.19) | 3.11 (1.69 - 7.74) | -0.80 (-1.06 to -0.55) |
| Peru | 71 (58 - 88) | 0.86 (0.70 - 1.06) | 28 (18 - 39) | 0.29 (0.19 - 0.41) | 2.96 (1.04 - 4.71) | -3.47 (-3.75 to -3.20) |
| Philippines | 151 (92 - 190) | 0.60 (0.36 - 0.75) | 107 (76 - 135) | 0.32 (0.22 - 0.40) | 1.96 (1.38 - 2.56) | -1.71 (-1.87 to -1.55) |
| Poland | 6 (6 - 6) | 0.06 (0.06 - 0.07) | 2 (2 - 3) | 0.04 (0.04 - 0.05) | 2.61 (2.23 - 3.03) | -3.84 (-5.56 to -2.08) |
| Portugal | 1 (1 - 1) | 0.05 (0.04 - 0.05) | 0 (0 - 0) | 0.03 (0.03 - 0.04) | 14.21 (11.85 - 16.44) | -2.54 (-2.97 to -2.11) |
| Puerto Rico | 1 (0 - 1) | 0.06 (0.05 - 0.06) | 0 (0 - 0) | 0.07 (0.06 - 0.08) | 5.65 (4.50 - 6.90) | 1.31 (0.68 - 1.94) |
| Qatar | 0 (0 - 0) | 0.01 (0.00 - 0.02) | 0 (0 - 0) | 0.00 (0.00 - 0.00) | 2.11 (0.71 - 9.40) | -5.36 (-5.91 to -4.80) |
| Republic of Korea | 3 (2 - 5) | 0.03 (0.02 - 0.04) | 1 (0 - 1) | 0.01 (0.01 - 0.01) | 5.34 (0.73 - 10.27) | -3.60 (-3.88 to -3.32) |
| Republic of Moldova | 3 (2 - 3) | 0.22 (0.19 - 0.25) | 1 (0 - 1) | 0.10 (0.08 - 0.13) | 0.26 (0.08 - 0.46) | -2.70 (-3.38 to -2.02) |
| Romania | 9 (8 - 10) | 0.16 (0.14 - 0.17) | 1 (1 - 1) | 0.04 (0.03 - 0.05) | 0.12 (-0.09 - 0.34) | -5.12 (-5.85 to -4.37) |
| Russian Federation | 46 (45 - 48) | 0.13 (0.13 - 0.14) | 17 (15 - 18) | 0.06 (0.06 - 0.07) | 1.01 (0.85 - 1.18) | -2.67 (-3.01 to -2.33) |
| Rwanda | 51 (28 - 85) | 1.49 (0.84 - 2.50) | 23 (15 - 33) | 0.46 (0.30 - 0.67) | 0.24 (-0.16 - 0.79) | -3.21 (-3.60 to -2.82) |
| Saint Kitts and Nevis | 0 (0 - 0) | 0.11 (0.10 - 0.13) | 0 (0 - 0) | 0.24 (0.19 - 0.30) | 3.28 (2.53 - 4.13) | 1.96 (1.31 - 2.61) |
| Saint Lucia | 0 (0 - 0) | 0.06 (0.05 - 0.08) | 0 (0 - 0) | 0.12 (0.09 - 0.16) | 6.98 (5.59 - 8.43) | 1.68 (1.03 - 2.33) |
| Saint Vincent and the Grenadines | 0 (0 - 0) | 0.12 (0.10 - 0.15) | 0 (0 - 0) | 0.23 (0.18 - 0.29) | 4.25 (3.51 - 5.08) | 1.40 (0.95 - 1.85) |
| Samoa | 0 (0 - 0) | 0.13 (0.09 - 0.20) | 0 (0 - 0) | 0.06 (0.04 - 0.09) | 0.42 (0.10 - 0.85) | -2.46 (-2.51 to -2.40) |
| San Marino | 0 (0 - 0) | 0.01 (0.01 - 0.01) | 0 (0 - 0) | 0.00 (0.00 - 0.01) | 1.40 (0.38 - 2.85) | -2.40 (-2.54 to -2.27) |
| Sao Tome and Principe | 1 (0 - 1) | 1.17 (0.79 - 1.60) | 0 (0 - 0) | 0.24 (0.13 - 0.60) | 0.50 (-0.03 - 1.13) | -3.85 (-4.29 to -3.41) |
| Saudi Arabia | 24 (16 - 33) | 0.37 (0.25 - 0.50) | 5 (3 - 7) | 0.06 (0.04 - 0.10) | 1.78 (0.59 - 3.12) | -5.32 (-5.54 to -5.09) |
| Senegal | 38 (21 - 55) | 1.04 (0.58 - 1.51) | 18 (13 - 26) | 0.29 (0.20 - 0.40) | 0.48 (-0.02 - 1.34) | -3.02 (-3.28 to -2.76) |
| Serbia | 1 (0 - 1) | 0.03 (0.02 - 0.04) | 0 (0 - 0) | 0.01 (0.00 - 0.01) | 0.41 (-0.07 - 1.15) | -4.98 (-5.40 to -4.55) |
| Seychelles | 0 (0 - 0) | 0.09 (0.06 - 0.16) | 0 (0 - 0) | 0.14 (0.08 - 0.19) | 3.08 (0.63 - 5.05) | 2.48 (2.05 - 2.90) |
| Sierra Leone | 30 (12 - 45) | 1.65 (0.67 - 2.49) | 19 (11 - 29) | 0.55 (0.30 - 0.80) | 0.15 (-0.21 - 0.71) | -3.10 (-3.29 to -2.92) |
| Singapore | 1 (1 - 1) | 0.14 (0.13 - 0.16) | 1 (1 - 1) | 0.09 (0.08 - 0.11) | 3.63 (2.99 - 4.19) | -2.31 (-2.97 to -1.64) |
| Slovakia | 1 (1 - 1) | 0.07 (0.05 - 0.08) | 0 (0 - 0) | 0.03 (0.02 - 0.04) | 0.16 (-0.07 - 0.50) | -2.76 (-2.98 to -2.54) |
| Slovenia | 0 (0 - 0) | 0.03 (0.02 - 0.03) | 0 (0 - 0) | 0.01 (0.01 - 0.02) | 0.81 (0.38 - 1.27) | -4.62 (-5.91 to -3.31) |
| Solomon Islands | 0 (0 - 0) | 0.14 (0.09 - 0.21) | 0 (0 - 0) | 0.07 (0.05 - 0.12) | 1.54 (0.86 - 2.50) | -1.73 (-1.93 to -1.54) |
| Somalia | 43 (22 - 72) | 1.11 (0.57 - 1.86) | 48 (25 - 76) | 0.46 (0.25 - 0.74) | 1.36 (0.70 - 2.26) | -1.81 (-2.07 to -1.54) |
| South Africa | 7 (4 - 10) | 0.05 (0.03 - 0.07) | 5 (3 - 7) | 0.03 (0.02 - 0.04) | 1.15 (0.59 - 1.96) | -1.75 (-1.92 to -1.58) |
| South Sudan | 38 (14 - 64) | 1.43 (0.54 - 2.42) | 50 (30 - 77) | 1.15 (0.71 - 1.80) | 0.62 (0.12 - 1.25) | -1.24 (-1.70 to -0.77) |
| Spain | 2 (2 - 3) | 0.03 (0.03 - 0.03) | 2 (2 - 2) | 0.03 (0.03 - 0.03) | 4.75 (3.85 - 5.51) | -0.83 (-1.35 to -0.30) |
| Sri Lanka | 8 (6 - 11) | 0.15 (0.10 - 0.19) | 4 (3 - 6) | 0.08 (0.05 - 0.11) | 2.35 (0.72 - 4.74) | -1.92 (-2.32 to -1.51) |
| Sudan | 33 (17 - 51) | 0.37 (0.19 - 0.58) | 29 (16 - 42) | 0.17 (0.10 - 0.25) | 1.11 (0.49 - 2.23) | -1.55 (-1.78 to -1.31) |
| Suriname | 0 (0 - 1) | 0.31 (0.24 - 0.43) | 1 (0 - 1) | 0.36 (0.22 - 0.50) | 3.75 (1.16 - 6.18) | 0.59 (0.39 - 0.78) |
| Sweden | 1 (1 - 1) | 0.04 (0.03 - 0.04) | 0 (0 - 0) | 0.01 (0.01 - 0.01) | 0.12 (-0.02 - 0.26) | -3.76 (-4.07 to -3.45) |
| Switzerland | 0 (0 - 0) | 0.02 (0.01 - 0.02) | 0 (0 - 0) | 0.01 (0.01 - 0.02) | 4.52 (3.81 - 5.15) | -1.33 (-1.77 to -0.88) |
| Syrian Arab Republic | 49 (33 - 65) | 0.82 (0.55 - 1.09) | 10 (7 - 14) | 0.26 (0.19 - 0.38) | 0.51 (-0.18 - 1.43) | -3.27 (-3.68 to -2.87) |
| Taiwan (Province of China) | 5 (4 - 5) | 0.08 (0.08 - 0.09) | 3 (2 - 3) | 0.09 (0.08 - 0.10) | 8.17 (6.84 - 9.34) | 0.26 (-0.38 - 0.90) |
| Tajikistan | 17 (13 - 22) | 0.75 (0.57 - 0.96) | 16 (11 - 28) | 0.46 (0.30 - 0.77) | 0.74 (0.23 - 1.59) | -1.55 (-1.78 to -1.33) |
| Thailand | 35 (21 - 60) | 0.21 (0.12 - 0.35) | 19 (15 - 24) | 0.20 (0.16 - 0.24) | 3.49 (1.36 - 6.47) | -0.61 (-0.85 to -0.37) |
| Timor-Leste | 2 (1 - 2) | 0.48 (0.22 - 0.69) | 1 (1 - 1) | 0.18 (0.13 - 0.23) | 1.74 (1.03 - 2.71) | -3.57 (-3.75 to -3.39) |
| Togo | 13 (7 - 19) | 0.74 (0.39 - 1.07) | 9 (6 - 13) | 0.27 (0.17 - 0.39) | 0.98 (0.32 - 1.87) | -2.46 (-2.62 to -2.30) |
| Tokelau | 0 (0 - 0) | 0.07 (0.04 - 0.11) | 0 (0 - 0) | 0.21 (0.14 - 0.40) | 0.13 (-0.14 - 0.47) | -1.82 (-2.86 to -0.78) |
| Tonga | 0 (0 - 0) | 0.12 (0.08 - 0.18) | 0 (0 - 0) | 0.08 (0.05 - 0.14) | 0.75 (0.27 - 1.37) | -0.79 (-1.01 to -0.56) |
| Trinidad and Tobago | 0 (0 - 0) | 0.07 (0.06 - 0.08) | 0 (0 - 0) | 0.13 (0.10 - 0.17) | 5.04 (3.56 - 6.65) | 2.38 (1.67 - 3.10) |
| Tunisia | 6 (4 - 8) | 0.18 (0.12 - 0.25) | 2 (1 - 2) | 0.06 (0.04 - 0.08) | 2.56 (1.50 - 4.24) | -3.16 (-3.29 to -3.03) |
| Turkey | 46 (31 - 65) | 0.23 (0.15 - 0.32) | 10 (7 - 13) | 0.05 (0.04 - 0.07) | 1.69 (0.64 - 2.82) | -4.35 (-4.46 to -4.25) |
| Turkmenistan | 6 (5 - 7) | 0.38 (0.33 - 0.43) | 11 (9 - 13) | 0.70 (0.58 - 0.85) | 7.17 (5.24 - 9.80) | 1.59 (1.28 - 1.91) |
| Tuvalu | 0 (0 - 0) | 0.29 (0.15 - 0.45) | 0 (0 - 0) | 0.07 (0.04 - 0.10) | 0.46 (0.14 - 0.85) | -4.92 (-5.12 to -4.72) |
| Uganda | 72 (38 - 114) | 0.85 (0.45 - 1.36) | 93 (57 - 139) | 0.47 (0.29 - 0.70) | 1.38 (0.73 - 2.46) | -1.60 (-1.73 to -1.47) |
| Ukraine | 12 (11 - 14) | 0.11 (0.09 - 0.13) | 3 (3 - 4) | 0.05 (0.05 - 0.07) | 0.06 (-0.20 - 0.40) | -2.07 (-2.34 to -1.81) |
| United Arab Emirates | 1 (0 - 1) | 0.10 (0.08 - 0.15) | 0 (0 - 1) | 0.03 (0.02 - 0.04) | 2.91 (1.36 - 4.76) | -3.32 (-3.63 to -3.01) |
| United Kingdom | 2 (2 - 2) | 0.02 (0.02 - 0.02) | 4 (4 - 5) | 0.04 (0.03 - 0.04) | 5.05 (4.61 - 5.36) | 2.29 (1.27 - 3.32) |
| United Republic of Tanzania | 168 (90 - 261) | 1.39 (0.75 - 2.16) | 152 (102 - 224) | 0.62 (0.42 - 0.92) | 0.93 (0.42 - 1.54) | -2.07 (-2.19 to -1.95) |
| United States of America | 32 (31 - 33) | 0.06 (0.06 - 0.06) | 23 (21 - 25) | 0.04 (0.03 - 0.04) | 0.96 (0.89 - 1.03) | -0.79 (-1.03 to -0.55) |
| United States Virgin Islands | 0 (0 - 0) | 0.10 (0.07 - 0.16) | 0 (0 - 0) | 0.05 (0.03 - 0.08) | 1.17 (0.52 - 2.02) | -1.68 (-1.95 to -1.40) |
| Uruguay | 0 (0 - 0) | 0.05 (0.04 - 0.05) | 1 (0 - 1) | 0.09 (0.08 - 0.11) | 12.65 (11.14 - 14.32) | 3.06 (2.35 - 3.77) |
| Uzbekistan | 20 (16 - 25) | 0.23 (0.19 - 0.29) | 20 (16 - 24) | 0.19 (0.16 - 0.24) | 1.18 (0.65 - 1.84) | 0.82 (0.16 - 1.49) |
| Vanuatu | 0 (0 - 0) | 0.10 (0.07 - 0.15) | 0 (0 - 0) | 0.06 (0.04 - 0.09) | 1.75 (1.10 - 2.66) | -1.40 (-1.69 to -1.11) |
| Venezuela (Bolivarian Republic of) | 10 (9 - 11) | 0.14 (0.12 - 0.15) | 11 (8 - 15) | 0.17 (0.12 - 0.22) | 8.11 (6.05 - 10.37) | -0.33 (-0.97 - 0.32) |
| Viet Nam | 5 (4 - 8) | 0.02 (0.01 - 0.03) | 3 (2 - 5) | 0.01 (0.01 - 0.02) | 1.44 (0.45 - 2.51) | -1.22 (-1.31 to -1.12) |
| Yemen | 16 (9 - 26) | 0.23 (0.12 - 0.36) | 17 (10 - 25) | 0.12 (0.07 - 0.18) | 1.87 (1.00 - 3.06) | -1.59 (-1.78 to -1.39) |
| Zambia | 42 (21 - 64) | 1.12 (0.57 - 1.69) | 31 (20 - 51) | 0.38 (0.24 - 0.61) | 1.51 (0.56 - 3.54) | -2.44 (-2.71 to -2.18) |
| Zimbabwe | 1 (1 - 2) | 0.03 (0.01 - 0.05) | 3 (2 - 5) | 0.05 (0.03 - 0.08) | 1.73 (0.75 - 3.30) | 0.58 (-0.21 - 1.38) |

**Table S3.** The DALYs and age-standardized DALYs Rate of Pediatric Urinary Tract Infection in 1990 and 2021, with Temporal Trends from 1990 to 2021 in 204 countries or territories.

| **location** | **Num_1990** | **DALYs_1990** | **Num_2021** | **DALYs_2021** | **Cases_change** | **EAPC_CI** |
| --- | --- | --- | --- | --- | --- | --- |
| Afghanistan | 1,751 (1,096 - 2,480) | 40.65 (25.45 - 57.57) | 3,414 (2,103 - 4,939) | 24.04 (14.81 - 34.78) | 0.90 (0.39 - 1.60) | -1.37 (-1.58 to -1.17) |
| Albania | 89 (51 - 138) | 7.98 (4.56 - 12.39) | 15 (10 - 21) | 3.33 (2.23 - 4.77) | -0.13 (-0.43 - 0.42) | -2.66 (-3.05 to -2.27) |
| Algeria | 2,143 (1,517 - 2,834) | 19.98 (14.14 - 26.43) | 1,318 (973 - 1,750) | 9.91 (7.32 - 13.15) | 1.19 (0.65 - 2.01) | -1.35 (-1.66 to -1.04) |
| American Samoa | 4 (3 - 5) | 20.68 (15.52 - 27.19) | 1 (1 - 2) | 9.02 (6.17 - 13.05) | 0.67 (0.27 - 1.12) | -2.74 (-2.94 to -2.53) |
| Andorra | 1 (0 - 1) | 6.63 (4.52 - 8.96) | 0 (0 - 0) | 2.32 (1.65 - 3.13) | 0.99 (0.32 - 1.92) | -3.26 (-3.47 to -3.05) |
| Angola | 1,897 (713 - 2,980) | 40.23 (15.13 - 63.20) | 2,089 (1,349 - 3,088) | 13.70 (8.85 - 20.25) | 1.04 (0.41 - 1.91) | -3.42 (-3.73 to -3.11) |
| Antigua and Barbuda | 1 (1 - 2) | 6.99 (5.40 - 9.15) | 2 (2 - 3) | 14.58 (12.38 - 17.39) | 4.01 (3.47 - 4.56) | 2.65 (2.10 - 3.20) |
| Argentina | 566 (493 - 669) | 5.58 (4.86 - 6.60) | 1,090 (931 - 1,271) | 10.70 (9.15 - 12.48) | 10.18 (8.83 - 11.46) | 2.73 (2.11 - 3.35) |
| Armenia | 242 (196 - 301) | 23.18 (18.74 - 28.82) | 94 (75 - 119) | 15.95 (12.66 - 20.06) | 0.30 (-0.00 - 0.76) | 0.58 (-0.32 - 1.49) |
| Australia | 297 (257 - 352) | 7.86 (6.78 - 9.30) | 253 (196 - 323) | 5.32 (4.13 - 6.80) | 2.06 (1.69 - 2.40) | -0.44 (-0.71 to -0.17) |
| Austria | 34 (24 - 49) | 2.49 (1.80 - 3.65) | 32 (22 - 48) | 2.45 (1.71 - 3.67) | 0.49 (0.32 - 0.67) | 0.36 (-0.03 - 0.74) |
| Azerbaijan | 729 (579 - 898) | 30.02 (23.87 - 37.02) | 511 (393 - 645) | 21.66 (16.65 - 27.31) | 0.95 (0.32 - 1.91) | -1.22 (-1.59 to -0.85) |
| Bahamas | 9 (7 - 11) | 10.61 (8.64 - 13.27) | 14 (11 - 18) | 16.99 (13.10 - 21.71) | 5.73 (4.31 - 7.49) | 1.69 (1.08 - 2.30) |
| Bahrain | 6 (4 - 9) | 3.62 (2.45 - 5.23) | 9 (6 - 14) | 3.14 (2.08 - 4.84) | 2.94 (1.77 - 5.88) | -0.87 (-1.31 to -0.43) |
| Bangladesh | 66,473 (36,543 - 88,489) | 135.90 (74.71 - 180.92) | 18,380 (14,100 - 23,858) | 40.16 (30.81 - 52.13) | 0.03 (-0.23 - 0.32) | -3.60 (-3.75 to -3.44) |
| Barbados | 10 (9 - 12) | 16.78 (14.32 - 19.84) | 12 (9 - 16) | 25.82 (19.20 - 34.67) | 3.64 (2.63 - 4.69) | 2.09 (1.36 - 2.83) |
| Belarus | 350 (291 - 421) | 14.56 (12.12 - 17.50) | 139 (108 - 181) | 8.82 (6.84 - 11.44) | 0.45 (0.18 - 0.78) | -1.28 (-1.72 to -0.83) |
| Belgium | 47 (38 - 61) | 2.62 (2.09 - 3.38) | 54 (42 - 70) | 2.84 (2.22 - 3.65) | 4.59 (3.79 - 5.31) | 0.59 (0.19 - 1.00) |
| Belize | 13 (11 - 16) | 16.32 (14.04 - 19.25) | 23 (19 - 28) | 18.43 (15.18 - 22.55) | 7.21 (6.03 - 8.51) | 1.07 (0.37 - 1.77) |
| Benin | 2,652 (1,428 - 3,795) | 109.51 (58.98 - 156.72) | 3,072 (1,923 - 4,455) | 50.53 (31.62 - 73.27) | 0.54 (0.11 - 1.27) | -2.14 (-2.32 to -1.97) |
| Bermuda | 1 (0 - 1) | 4.84 (3.41 - 7.03) | 1 (0 - 1) | 6.21 (4.52 - 8.50) | 2.91 (2.19 - 3.78) | 1.06 (0.83 - 1.30) |
| Bhutan | 279 (142 - 431) | 106.28 (53.99 - 164.30) | 103 (66 - 155) | 55.03 (35.29 - 82.80) | 0.38 (-0.07 - 1.16) | -2.40 (-2.71 to -2.09) |
| Bolivia (Plurinational State of) | 2,457 (1,916 - 3,151) | 91.47 (71.35 - 117.33) | 1,283 (888 - 1,773) | 36.79 (25.47 - 50.86) | 0.88 (0.29 - 1.52) | -2.58 (-2.69 to -2.48) |
| Bosnia and Herzegovina | 59 (43 - 82) | 5.41 (3.90 - 7.48) | 21 (14 - 30) | 4.19 (2.76 - 6.18) | -0.21 (-0.49 - 0.31) | -0.98 (-1.26 to -0.70) |
| Botswana | 23 (14 - 33) | 3.87 (2.35 - 5.55) | 34 (20 - 49) | 4.90 (2.90 - 7.09) | 1.39 (0.67 - 2.44) | 1.37 (1.14 - 1.60) |
| Brazil | 28,042 (25,010 - 31,071) | 53.98 (48.15 - 59.82) | 17,765 (14,534 - 21,178) | 36.87 (30.16 - 43.95) | 4.13 (3.67 - 4.49) | -0.16 (-0.51 - 0.20) |
| Brunei Darussalam | 11 (8 - 15) | 11.79 (8.75 - 16.91) | 11 (8 - 14) | 11.31 (8.41 - 15.00) | 1.79 (0.73 - 2.87) | 0.75 (0.44 - 1.07) |
| Bulgaria | 338 (295 - 384) | 19.48 (16.96 - 22.10) | 109 (87 - 136) | 11.21 (8.89 - 13.97) | -0.64 (-0.72 to -0.55) | -1.61 (-2.40 to -0.82) |
| Burkina Faso | 4,346 (1,998 - 6,490) | 92.10 (42.34 - 137.52) | 5,546 (2,909 - 8,019) | 53.47 (28.04 - 77.31) | 0.53 (0.09 - 1.13) | -1.44 (-1.61 to -1.27) |
| Burundi | 2,714 (1,312 - 4,459) | 103.55 (50.05 - 170.13) | 1,738 (991 - 3,020) | 29.69 (16.93 - 51.58) | 0.27 (-0.13 - 0.84) | -3.23 (-3.61 to -2.84) |
| Cabo Verde | 33 (15 - 48) | 21.01 (9.84 - 30.30) | 9 (6 - 14) | 6.40 (4.51 - 9.80) | 0.36 (0.02 - 0.80) | -4.07 (-4.31 to -3.83) |
| Cambodia | 1,761 (1,013 - 2,377) | 37.79 (21.74 - 51.00) | 752 (569 - 957) | 14.71 (11.13 - 18.71) | 0.81 (0.33 - 1.43) | -3.31 (-3.46 to -3.16) |
| Cameroon | 4,974 (2,917 - 6,897) | 101.88 (59.75 - 141.27) | 5,991 (3,762 - 8,534) | 44.49 (27.94 - 63.37) | 0.72 (0.24 - 1.47) | -2.18 (-2.39 to -1.97) |
| Canada | 268 (220 - 336) | 4.66 (3.82 - 5.84) | 213 (159 - 291) | 3.45 (2.58 - 4.71) | 1.89 (1.62 - 2.17) | -0.61 (-0.78 to -0.44) |
| Central African Republic | 479 (227 - 730) | 39.21 (18.58 - 59.69) | 538 (321 - 775) | 23.55 (14.03 - 33.92) | 0.61 (0.14 - 1.30) | -1.35 (-1.49 to -1.22) |
| Chad | 2,261 (955 - 3,336) | 77.27 (32.64 - 114.00) | 4,249 (1,804 - 6,609) | 47.14 (20.01 - 73.31) | 1.07 (0.47 - 1.89) | -1.53 (-1.60 to -1.47) |
| Chile | 283 (253 - 327) | 7.12 (6.36 - 8.24) | 329 (281 - 399) | 9.02 (7.69 - 10.94) | 2.85 (2.50 - 3.18) | 1.35 (0.90 - 1.81) |
| China | 32,984 (20,005 - 41,196) | 10.36 (6.28 - 12.94) | 3,687 (3,003 - 4,806) | 1.42 (1.16 - 1.85) | -0.12 (-0.45 - 0.54) | -6.86 (-7.15 to -6.57) |
| Colombia | 3,817 (3,289 - 4,461) | 32.73 (28.20 - 38.25) | 2,607 (2,010 - 3,352) | 24.57 (18.94 - 31.59) | 2.73 (2.14 - 3.36) | 0.07 (-0.24 - 0.38) |
| Comoros | 188 (105 - 294) | 88.38 (49.34 - 138.40) | 112 (76 - 165) | 46.55 (31.76 - 68.56) | 0.66 (0.14 - 1.60) | -2.23 (-2.48 to -1.98) |
| Congo | 274 (147 - 408) | 25.99 (14.00 - 38.72) | 186 (127 - 262) | 9.64 (6.56 - 13.56) | 0.81 (0.31 - 1.51) | -3.36 (-3.80 to -2.92) |
| Cook Islands | 0 (0 - 0) | 0.93 (0.62 - 1.26) | 0 (0 - 0) | 0.51 (0.33 - 0.78) | 0.21 (-0.04 - 0.53) | -3.34 (-3.80 to -2.88) |
| Costa Rica | 157 (130 - 190) | 13.98 (11.58 - 16.94) | 141 (115 - 173) | 13.89 (11.32 - 17.05) | 4.73 (4.03 - 5.47) | 0.62 (0.31 - 0.93) |
| Côte d'Ivoire | 4,165 (2,517 - 5,799) | 73.02 (44.13 - 101.67) | 4,090 (2,702 - 5,706) | 35.34 (23.35 - 49.31) | 0.53 (0.10 - 1.16) | -1.98 (-2.23 to -1.72) |
| Croatia | 127 (109 - 149) | 12.86 (11.05 - 15.06) | 42 (33 - 54) | 7.07 (5.45 - 9.10) | 0.45 (0.20 - 0.74) | -1.16 (-1.73 to -0.59) |
| Cuba | 117 (82 - 168) | 4.69 (3.27 - 6.70) | 104 (77 - 141) | 5.83 (4.32 - 7.91) | 3.78 (2.93 - 4.82) | 1.19 (1.01 - 1.38) |
| Cyprus | 18 (14 - 24) | 9.25 (7.16 - 12.15) | 6 (5 - 8) | 2.73 (2.07 - 3.57) | 0.54 (0.12 - 1.07) | -3.45 (-3.79 to -3.10) |
| Czechia | 319 (281 - 366) | 14.50 (12.77 - 16.60) | 179 (130 - 251) | 10.45 (7.58 - 14.61) | -0.30 (-0.45 to -0.11) | -0.91 (-1.85 - 0.03) |
| Democratic People's Republic of Korea | 506 (323 - 752) | 8.51 (5.42 - 12.63) | 142 (81 - 274) | 2.98 (1.69 - 5.75) | 0.44 (-0.07 - 1.14) | -3.18 (-3.40 to -2.95) |
| Democratic Republic of the Congo | 5,787 (2,704 - 8,699) | 32.69 (15.27 - 49.14) | 3,675 (2,482 - 5,224) | 9.67 (6.53 - 13.75) | 0.58 (0.10 - 1.19) | -3.50 (-3.76 to -3.24) |
| Denmark | 31 (26 - 39) | 3.55 (2.92 - 4.38) | 29 (22 - 37) | 3.02 (2.35 - 3.86) | 1.81 (1.48 - 2.17) | -0.25 (-0.52 - 0.02) |
| Djibouti | 99 (55 - 153) | 56.74 (31.58 - 87.99) | 126 (77 - 193) | 30.42 (18.67 - 46.65) | 2.81 (1.47 - 4.84) | -1.92 (-2.39 to -1.45) |
| Dominica | 1 (1 - 2) | 5.32 (3.91 - 7.32) | 1 (1 - 2) | 10.84 (7.01 - 15.04) | 1.45 (0.37 - 2.36) | 2.75 (2.51 - 2.98) |
| Dominican Republic | 301 (228 - 405) | 11.17 (8.44 - 15.02) | 174 (116 - 272) | 5.91 (3.94 - 9.27) | 1.05 (0.64 - 1.50) | -1.68 (-1.90 to -1.46) |
| Ecuador | 486 (431 - 554) | 12.58 (11.16 - 14.32) | 925 (716 - 1,197) | 18.23 (14.11 - 23.61) | 4.64 (3.47 - 6.08) | 1.32 (0.44 - 2.21) |
| Egypt | 805 (531 - 1,164) | 3.63 (2.39 - 5.25) | 1,130 (799 - 1,559) | 3.07 (2.17 - 4.23) | 2.31 (1.03 - 3.61) | 0.48 (0.12 - 0.85) |
| El Salvador | 1,307 (1,030 - 1,643) | 60.57 (47.75 - 76.12) | 376 (279 - 515) | 20.65 (15.36 - 28.33) | 0.66 (0.20 - 1.17) | -3.02 (-3.42 to -2.62) |
| Equatorial Guinea | 59 (29 - 90) | 29.92 (14.63 - 45.83) | 71 (42 - 111) | 12.11 (7.15 - 18.93) | 1.51 (0.63 - 2.86) | -3.16 (-3.35 to -2.96) |
| Eritrea | 1,050 (577 - 1,643) | 65.97 (36.27 - 103.20) | 870 (523 - 1,460) | 34.47 (20.73 - 57.82) | 0.75 (0.23 - 1.48) | -2.00 (-2.18 to -1.82) |
| Estonia | 67 (59 - 78) | 19.31 (17.00 - 22.35) | 17 (13 - 23) | 7.94 (6.08 - 10.57) | -0.19 (-0.33 to -0.03) | -2.31 (-2.90 to -1.72) |
| Eswatini | 24 (15 - 33) | 6.14 (3.81 - 8.67) | 23 (14 - 33) | 5.57 (3.36 - 7.91) | 1.26 (0.44 - 2.49) | -0.01 (-0.12 - 0.10) |
| Ethiopia | 42,854 (22,710 - 63,207) | 175.89 (93.21 - 259.43) | 23,304 (16,129 - 31,863) | 52.55 (36.37 - 71.84) | 0.00 (-0.23 - 0.33) | -4.11 (-4.37 to -3.84) |
| Fiji | 11 (8 - 15) | 3.86 (2.77 - 5.29) | 16 (10 - 25) | 5.79 (3.80 - 9.02) | 1.49 (0.60 - 2.71) | 2.29 (1.66 - 2.93) |
| Finland | 28 (22 - 37) | 2.88 (2.23 - 3.81) | 26 (20 - 35) | 3.07 (2.41 - 4.09) | 0.06 (-0.05 - 0.16) | 0.29 (-0.11 - 0.69) |
| France | 276 (216 - 360) | 2.35 (1.84 - 3.07) | 299 (236 - 388) | 2.58 (2.04 - 3.34) | 0.93 (0.73 - 1.14) | 0.57 (0.33 - 0.81) |
| Gabon | 77 (45 - 115) | 18.99 (11.00 - 28.16) | 70 (45 - 106) | 11.01 (7.00 - 16.53) | 0.87 (0.22 - 1.62) | -1.17 (-1.57 to -0.77) |
| Gambia | 280 (164 - 408) | 60.77 (35.50 - 88.42) | 239 (157 - 340) | 24.11 (15.79 - 34.27) | 0.80 (0.23 - 1.79) | -3.18 (-3.48 to -2.88) |
| Georgia | 90 (75 - 112) | 6.59 (5.45 - 8.19) | 32 (23 - 43) | 4.32 (3.07 - 5.84) | 0.37 (0.13 - 0.70) | -0.31 (-0.97 - 0.36) |
| Germany | 326 (255 - 439) | 2.52 (1.97 - 3.39) | 302 (233 - 408) | 2.53 (1.95 - 3.41) | 2.23 (1.80 - 2.65) | 0.64 (0.32 - 0.96) |
| Ghana | 3,764 (1,987 - 5,602) | 56.04 (29.59 - 83.41) | 4,107 (2,473 - 7,487) | 31.88 (19.20 - 58.11) | 1.51 (0.77 - 2.62) | -1.08 (-1.32 to -0.84) |
| Greece | 40 (29 - 56) | 1.97 (1.43 - 2.79) | 31 (23 - 42) | 2.19 (1.63 - 3.01) | 1.95 (1.60 - 2.30) | 0.80 (0.63 - 0.98) |
| Greenland | 2 (1 - 2) | 10.62 (7.64 - 16.25) | 1 (0 - 1) | 4.88 (3.34 - 7.81) | 0.29 (-0.00 - 0.60) | -2.67 (-2.87 to -2.46) |
| Grenada | 3 (2 - 4) | 8.55 (6.77 - 10.63) | 3 (2 - 3) | 11.56 (9.19 - 14.38) | 2.10 (1.38 - 2.83) | 1.60 (1.26 - 1.95) |
| Guam | 5 (4 - 6) | 11.43 (8.73 - 14.58) | 4 (3 - 5) | 9.93 (7.05 - 13.22) | 0.55 (0.24 - 1.02) | 0.27 (-0.14 - 0.69) |
| Guatemala | 1,435 (1,261 - 1,634) | 35.34 (31.05 - 40.23) | 1,435 (1,128 - 1,815) | 29.08 (22.86 - 36.79) | 2.79 (2.16 - 3.44) | 0.42 (-0.08 - 0.91) |
| Guinea | 3,538 (1,579 - 5,170) | 128.57 (57.37 - 187.86) | 2,620 (1,564 - 3,719) | 43.34 (25.87 - 61.51) | 0.10 (-0.24 - 0.72) | -2.87 (-3.05 to -2.69) |
| Guinea-Bissau | 548 (280 - 823) | 113.60 (58.08 - 170.58) | 257 (170 - 366) | 28.66 (18.92 - 40.79) | -0.05 (-0.37 - 0.50) | -4.01 (-4.37 to -3.65) |
| Guyana | 40 (33 - 49) | 13.68 (11.32 - 16.61) | 45 (35 - 58) | 21.31 (16.19 - 27.36) | 2.66 (1.71 - 3.67) | 2.52 (1.91 - 3.14) |
| Haiti | 1,602 (705 - 2,254) | 59.04 (25.99 - 83.10) | 1,967 (927 - 3,136) | 45.20 (21.30 - 72.04) | 1.08 (0.28 - 2.06) | -0.29 (-0.50 to -0.08) |
| Honduras | 1,736 (1,388 - 2,164) | 78.59 (62.81 - 97.94) | 751 (441 - 1,395) | 22.91 (13.45 - 42.58) | 1.57 (0.71 - 2.46) | -3.83 (-3.88 to -3.78) |
| Hungary | 241 (208 - 280) | 11.30 (9.74 - 13.13) | 89 (66 - 119) | 6.40 (4.76 - 8.59) | -0.06 (-0.21 - 0.13) | -1.10 (-1.85 to -0.34) |
| Iceland | 1 (1 - 2) | 1.62 (1.07 - 2.41) | 1 (1 - 2) | 2.00 (1.41 - 2.77) | 3.19 (2.57 - 3.82) | 0.86 (0.43 - 1.29) |
| India | 297,897 (202,236 - 382,054) | 91.23 (61.94 - 117.01) | 160,543 (98,415 - 243,630) | 43.82 (26.86 - 66.49) | 0.92 (0.55 - 1.27) | -2.20 (-2.33 to -2.08) |
| Indonesia | 8,467 (5,400 - 11,053) | 12.50 (7.97 - 16.32) | 4,832 (3,847 - 5,981) | 7.18 (5.72 - 8.89) | 1.45 (0.89 - 2.07) | -1.69 (-1.79 to -1.58) |
| Iran (Islamic Republic of) | 9,703 (5,480 - 12,518) | 38.22 (21.59 - 49.32) | 1,273 (1,025 - 1,553) | 6.31 (5.08 - 7.70) | 0.18 (-0.21 - 0.89) | -3.83 (-4.62 to -3.03) |
| Iraq | 591 (417 - 854) | 7.18 (5.06 - 10.37) | 448 (323 - 607) | 3.33 (2.40 - 4.51) | 0.86 (0.20 - 1.46) | -2.20 (-2.34 to -2.07) |
| Ireland | 30 (24 - 37) | 3.05 (2.48 - 3.81) | 25 (19 - 33) | 2.52 (1.95 - 3.35) | 1.01 (0.78 - 1.25) | -0.10 (-0.63 - 0.43) |
| Israel | 89 (79 - 101) | 5.79 (5.13 - 6.61) | 104 (86 - 127) | 3.94 (3.26 - 4.82) | 4.90 (4.16 - 5.56) | -0.33 (-0.72 - 0.06) |
| Italy | 250 (204 - 311) | 2.71 (2.21 - 3.37) | 185 (144 - 238) | 2.43 (1.89 - 3.14) | 1.81 (1.39 - 2.30) | 0.32 (0.14 - 0.50) |
| Jamaica | 99 (83 - 119) | 11.80 (9.92 - 14.26) | 80 (62 - 102) | 13.69 (10.57 - 17.48) | 4.14 (2.98 - 5.59) | 1.05 (0.64 - 1.47) |
| Japan | 1,493 (1,071 - 2,074) | 6.47 (4.64 - 8.98) | 981 (710 - 1,342) | 6.35 (4.60 - 8.69) | 3.33 (2.74 - 3.81) | 0.46 (0.32 - 0.60) |
| Jordan | 125 (97 - 158) | 7.68 (5.95 - 9.69) | 197 (144 - 274) | 5.42 (3.95 - 7.53) | 2.68 (1.68 - 3.98) | -1.01 (-1.12 to -0.90) |
| Kazakhstan | 1,025 (876 - 1,209) | 19.73 (16.85 - 23.26) | 1,121 (934 - 1,358) | 20.67 (17.22 - 25.03) | 0.53 (0.22 - 0.88) | 0.10 (-0.29 - 0.49) |
| Kenya | 5,468 (3,531 - 7,403) | 48.96 (31.61 - 66.27) | 4,181 (2,997 - 6,971) | 22.40 (16.06 - 37.35) | 1.89 (1.18 - 2.86) | -1.47 (-1.85 to -1.08) |
| Kiribati | 11 (7 - 15) | 36.19 (23.80 - 51.47) | 6 (4 - 11) | 14.46 (9.26 - 25.07) | 0.58 (0.19 - 1.10) | -2.89 (-3.03 to -2.75) |
| Kuwait | 18 (12 - 24) | 3.16 (2.14 - 4.38) | 85 (71 - 104) | 10.11 (8.37 - 12.26) | 14.96 (11.10 - 20.49) | 3.53 (2.53 - 4.54) |
| Kyrgyzstan | 458 (402 - 522) | 27.33 (23.94 - 31.11) | 337 (298 - 382) | 14.82 (13.08 - 16.81) | 0.39 (0.11 - 0.75) | -0.47 (-1.32 - 0.38) |
| Lao People's Democratic Republic | 885 (469 - 1,247) | 48.02 (25.45 - 67.63) | 431 (303 - 602) | 18.78 (13.20 - 26.20) | 0.35 (-0.00 - 0.86) | -3.19 (-3.32 to -3.05) |
| Latvia | 115 (100 - 134) | 20.27 (17.64 - 23.51) | 24 (18 - 32) | 8.00 (5.99 - 10.69) | -0.18 (-0.35 - 0.06) | -2.47 (-2.97 to -1.97) |
| Lebanon | 278 (203 - 364) | 26.55 (19.42 - 34.77) | 126 (91 - 166) | 9.89 (7.13 - 13.00) | 1.27 (0.77 - 1.85) | -3.16 (-3.44 to -2.88) |
| Lesotho | 26 (16 - 36) | 3.75 (2.34 - 5.32) | 33 (20 - 49) | 5.29 (3.23 - 7.71) | 1.66 (0.79 - 2.93) | 1.79 (1.54 - 2.04) |
| Liberia | 1,887 (902 - 2,715) | 166.98 (79.82 - 240.23) | 702 (420 - 1,014) | 32.14 (19.21 - 46.40) | -0.19 (-0.47 - 0.29) | -4.68 (-5.38 to -3.98) |
| Libya | 209 (156 - 281) | 11.55 (8.59 - 15.53) | 182 (114 - 269) | 12.23 (7.65 - 18.03) | 2.10 (0.62 - 3.98) | 0.91 (0.70 - 1.13) |
| Lithuania | 112 (94 - 135) | 13.47 (11.32 - 16.25) | 33 (24 - 45) | 8.12 (5.92 - 10.97) | 0.17 (-0.05 - 0.44) | -1.65 (-1.98 to -1.32) |
| Luxembourg | 1 (1 - 2) | 1.65 (1.14 - 2.35) | 2 (1 - 2) | 1.55 (1.04 - 2.19) | 2.60 (2.09 - 3.11) | -0.17 (-0.37 - 0.02) |
| Madagascar | 3,925 (2,071 - 5,962) | 71.94 (37.97 - 109.28) | 2,986 (2,015 - 4,486) | 25.45 (17.17 - 38.23) | 0.60 (0.16 - 1.15) | -2.88 (-3.04 to -2.72) |
| Malawi | 3,467 (1,675 - 5,117) | 76.21 (36.82 - 112.48) | 1,801 (1,276 - 2,619) | 22.17 (15.71 - 32.24) | 0.55 (0.19 - 1.03) | -3.78 (-3.99 to -3.56) |
| Malaysia | 497 (344 - 670) | 7.55 (5.23 - 10.19) | 346 (261 - 450) | 4.54 (3.42 - 5.91) | 1.85 (0.97 - 4.91) | -1.08 (-1.44 to -0.71) |
| Maldives | 31 (21 - 41) | 29.95 (19.96 - 39.00) | 9 (7 - 12) | 8.78 (6.70 - 11.94) | 0.49 (0.18 - 0.84) | -3.26 (-3.64 to -2.87) |
| Mali | 4,731 (1,840 - 7,120) | 114.56 (44.55 - 172.40) | 4,965 (2,487 - 7,254) | 42.89 (21.48 - 62.67) | 0.37 (-0.03 - 1.02) | -2.94 (-3.07 to -2.82) |
| Malta | 3 (2 - 3) | 3.17 (2.68 - 3.87) | 3 (2 - 3) | 4.09 (3.24 - 4.92) | 3.12 (2.61 - 3.68) | 1.46 (1.16 - 1.77) |
| Marshall Islands | 2 (1 - 2) | 7.30 (5.06 - 10.57) | 1 (1 - 2) | 5.77 (3.63 - 9.39) | 1.01 (0.44 - 1.70) | -0.90 (-1.48 to -0.31) |
| Mauritania | 511 (329 - 713) | 55.24 (35.55 - 77.13) | 375 (250 - 519) | 20.26 (13.51 - 28.03) | 0.24 (-0.15 - 0.83) | -3.19 (-3.53 to -2.85) |
| Mauritius | 6 (6 - 8) | 1.95 (1.71 - 2.28) | 12 (10 - 15) | 5.89 (4.91 - 7.42) | 10.10 (8.85 - 11.41) | 4.11 (3.55 - 4.68) |
| Mexico | 14,287 (12,915 - 16,084) | 42.76 (38.65 - 48.13) | 9,204 (7,411 - 11,632) | 28.70 (23.11 - 36.27) | 3.68 (3.03 - 4.55) | -0.44 (-0.93 - 0.06) |
| Micronesia (Federated States of) | 6 (4 - 8) | 12.59 (8.77 - 17.63) | 2 (1 - 3) | 5.16 (3.48 - 8.36) | 0.23 (-0.11 - 0.71) | -3.03 (-3.20 to -2.86) |
| Monaco | 0 (0 - 0) | 1.51 (1.00 - 2.22) | 0 (0 - 0) | 1.51 (1.02 - 2.23) | 0.91 (0.28 - 1.56) | -0.30 (-0.42 to -0.19) |
| Mongolia | 240 (174 - 320) | 26.63 (19.38 - 35.60) | 109 (82 - 142) | 10.01 (7.58 - 13.07) | 0.27 (-0.07 - 0.74) | -2.92 (-3.40 to -2.44) |
| Montenegro | 6 (4 - 9) | 3.59 (2.34 - 5.41) | 4 (2 - 5) | 3.17 (1.99 - 4.86) | 0.25 (0.05 - 0.51) | -0.38 (-0.44 to -0.33) |
| Morocco | 2,477 (1,540 - 3,480) | 25.31 (15.73 - 35.56) | 1,021 (698 - 1,378) | 10.42 (7.13 - 14.07) | 1.17 (0.65 - 1.95) | -2.37 (-2.80 to -1.95) |
| Mozambique | 9,911 (5,039 - 16,511) | 159.75 (81.22 - 266.13) | 6,942 (4,465 - 11,719) | 48.66 (31.30 - 82.15) | 0.76 (0.15 - 1.59) | -3.34 (-3.55 to -3.13) |
| Myanmar | 7,178 (3,366 - 10,176) | 48.58 (22.78 - 68.87) | 3,630 (2,589 - 4,814) | 23.24 (16.58 - 30.83) | 0.29 (-0.05 - 0.73) | -2.54 (-2.81 to -2.28) |
| Namibia | 22 (14 - 33) | 3.68 (2.28 - 5.51) | 28 (17 - 40) | 3.40 (2.04 - 4.89) | 1.20 (0.47 - 2.15) | 0.19 (-0.03 - 0.42) |
| Nauru | 1 (0 - 1) | 12.50 (8.65 - 18.07) | 0 (0 - 1) | 9.40 (6.33 - 14.38) | 0.13 (-0.14 - 0.53) | -1.06 (-1.70 to -0.41) |
| Nepal | 8,893 (5,378 - 12,073) | 105.56 (63.83 - 143.30) | 3,485 (2,441 - 4,990) | 37.77 (26.46 - 54.08) | 0.65 (0.23 - 1.21) | -2.94 (-3.12 to -2.75) |
| Netherlands | 122 (105 - 141) | 4.48 (3.86 - 5.18) | 129 (108 - 151) | 4.80 (4.02 - 5.65) | 1.27 (1.06 - 1.48) | 0.50 (0.18 - 0.83) |
| New Zealand | 117 (105 - 132) | 14.63 (13.07 - 16.52) | 70 (57 - 86) | 7.09 (5.76 - 8.76) | 0.57 (0.38 - 0.77) | -0.91 (-1.64 to -0.18) |
| Nicaragua | 521 (403 - 663) | 28.62 (22.12 - 36.40) | 218 (157 - 303) | 11.01 (7.93 - 15.28) | 1.04 (0.47 - 1.64) | -2.37 (-2.66 to -2.07) |
| Niger | 5,321 (2,244 - 7,936) | 130.97 (55.23 - 195.34) | 4,288 (2,570 - 6,269) | 33.60 (20.13 - 49.12) | 0.13 (-0.26 - 0.96) | -4.58 (-4.88 to -4.29) |
| Nigeria | 32,178 (15,925 - 42,958) | 82.24 (40.70 - 109.80) | 45,633 (25,862 - 64,761) | 44.92 (25.46 - 63.75) | 0.67 (0.32 - 1.17) | -1.68 (-1.81 to -1.55) |
| Niue | 0 (0 - 0) | 7.36 (5.09 - 10.41) | 0 (0 - 0) | 25.19 (19.83 - 32.94) | -0.07 (-0.34 - 0.25) | 1.16 (0.20 - 2.13) |
| North Macedonia | 22 (15 - 30) | 4.12 (2.82 - 5.62) | 9 (6 - 14) | 2.74 (1.70 - 4.15) | 0.16 (-0.07 - 0.48) | -0.96 (-1.21 to -0.71) |
| Northern Mariana Islands | 1 (1 - 2) | 11.79 (8.48 - 17.04) | 1 (0 - 1) | 5.07 (3.71 - 7.07) | 0.89 (0.43 - 1.55) | -2.86 (-3.36 to -2.35) |
| Norway | 23 (18 - 30) | 2.91 (2.29 - 3.78) | 19 (14 - 27) | 2.08 (1.47 - 2.87) | 0.84 (0.71 - 0.96) | -0.32 (-0.71 - 0.07) |
| Oman | 186 (128 - 246) | 22.14 (15.19 - 29.32) | 129 (103 - 160) | 10.57 (8.40 - 13.12) | 1.25 (0.52 - 2.30) | -1.01 (-1.63 to -0.38) |
| Pakistan | 61,852 (45,014 - 82,992) | 125.61 (91.41 - 168.54) | 92,162 (68,739 - 124,985) | 107.86 (80.45 - 146.28) | 1.45 (0.94 - 2.10) | 0.51 (0.18 - 0.85) |
| Palau | 1 (0 - 1) | 12.20 (7.77 - 18.75) | 0 (0 - 0) | 6.11 (4.39 - 8.52) | 0.68 (0.24 - 1.25) | -1.93 (-2.24 to -1.63) |
| Palestine | 68 (44 - 104) | 7.03 (4.50 - 10.73) | 75 (51 - 101) | 4.01 (2.74 - 5.42) | 1.22 (0.62 - 1.95) | -1.37 (-1.54 to -1.20) |
| Panama | 144 (120 - 168) | 17.25 (14.42 - 20.14) | 245 (199 - 299) | 21.27 (17.27 - 25.95) | 3.78 (2.92 - 4.76) | 1.33 (1.06 - 1.59) |
| Papua New Guinea | 306 (145 - 505) | 18.00 (8.52 - 29.73) | 563 (311 - 869) | 14.37 (7.95 - 22.18) | 1.33 (0.68 - 2.34) | -0.57 (-0.74 to -0.40) |
| Paraguay | 239 (162 - 335) | 14.34 (9.68 - 20.04) | 277 (191 - 389) | 13.78 (9.50 - 19.37) | 1.80 (1.06 - 3.71) | -0.18 (-0.40 - 0.04) |
| Peru | 6,461 (5,297 - 7,966) | 77.83 (63.82 - 95.97) | 2,688 (1,785 - 3,687) | 28.18 (18.72 - 38.66) | 1.23 (0.35 - 2.03) | -2.65 (-2.93 to -2.36) |
| Philippines | 12,841 (7,793 - 16,211) | 50.93 (30.91 - 64.29) | 9,064 (6,461 - 11,386) | 26.66 (19.00 - 33.49) | 1.27 (0.89 - 1.74) | -1.39 (-1.59 to -1.19) |
| Poland | 914 (765 - 1,106) | 9.54 (7.99 - 11.55) | 338 (280 - 416) | 5.74 (4.75 - 7.07) | 1.55 (1.26 - 1.91) | -1.72 (-2.55 to -0.87) |
| Portugal | 105 (93 - 121) | 4.96 (4.37 - 5.70) | 49 (40 - 60) | 3.60 (2.92 - 4.43) | 7.10 (5.79 - 8.26) | -1.12 (-1.40 to -0.85) |
| Puerto Rico | 85 (69 - 107) | 8.50 (6.93 - 10.71) | 43 (34 - 54) | 9.72 (7.68 - 12.19) | 3.20 (2.51 - 3.94) | 0.89 (0.32 - 1.47) |
| Qatar | 5 (3 - 7) | 3.66 (2.10 - 5.49) | 13 (8 - 20) | 2.67 (1.67 - 4.07) | 3.91 (2.35 - 7.22) | -1.58 (-1.90 to -1.26) |
| Republic of Korea | 632 (445 - 882) | 5.56 (3.91 - 7.76) | 242 (160 - 357) | 3.99 (2.63 - 5.88) | 2.18 (0.18 - 4.07) | -0.90 (-1.05 to -0.76) |
| Republic of Moldova | 280 (244 - 328) | 22.63 (19.73 - 26.53) | 63 (49 - 81) | 12.13 (9.47 - 15.42) | 0.08 (-0.08 - 0.25) | -1.73 (-2.55 to -0.89) |
| Romania | 882 (771 - 1,000) | 15.85 (13.85 - 17.97) | 243 (183 - 327) | 8.07 (6.07 - 10.87) | -0.21 (-0.35 to -0.05) | -2.19 (-2.92 to -1.46) |
| Russian Federation | 5,641 (4,948 - 6,548) | 16.26 (14.26 - 18.87) | 3,196 (2,476 - 4,115) | 12.25 (9.50 - 15.78) | 0.54 (0.41 - 0.70) | -1.09 (-1.52 to -0.66) |
| Rwanda | 4,381 (2,451 - 7,310) | 129.13 (72.24 - 215.44) | 1,962 (1,278 - 2,883) | 39.47 (25.72 - 58.01) | -0.00 (-0.33 - 0.46) | -4.39 (-4.76 to -4.02) |
| Saint Kitts and Nevis | 2 (2 - 2) | 12.86 (11.01 - 15.26) | 2 (2 - 3) | 23.57 (18.84 - 29.11) | 3.23 (2.44 - 4.12) | 2.72 (2.16 - 3.27) |
| Saint Lucia | 4 (4 - 6) | 8.55 (6.89 - 10.83) | 4 (3 - 5) | 13.81 (10.55 - 17.57) | 4.86 (3.79 - 6.09) | 2.08 (1.63 - 2.54) |
| Saint Vincent and the Grenadines | 6 (5 - 7) | 13.67 (11.30 - 16.50) | 6 (5 - 7) | 22.19 (18.04 - 27.30) | 3.45 (2.83 - 4.16) | 1.57 (1.17 - 1.96) |
| Samoa | 9 (6 - 13) | 12.04 (8.01 - 17.83) | 5 (3 - 7) | 5.68 (3.74 - 8.81) | 0.32 (0.01 - 0.74) | -2.28 (-2.35 to -2.20) |
| San Marino | 0 (0 - 0) | 1.88 (1.24 - 2.68) | 0 (0 - 0) | 1.37 (0.84 - 2.11) | 0.85 (0.21 - 1.68) | -0.81 (-0.87 to -0.74) |
| Sao Tome and Principe | 58 (39 - 80) | 102.87 (69.58 - 140.79) | 16 (9 - 41) | 21.16 (11.63 - 53.13) | 0.19 (-0.18 - 0.73) | -4.88 (-5.37 to -4.39) |
| Saudi Arabia | 2,216 (1,531 - 2,993) | 33.82 (23.36 - 45.67) | 545 (394 - 790) | 7.21 (5.21 - 10.44) | 1.71 (0.71 - 2.94) | -5.27 (-5.51 to -5.03) |
| Senegal | 3,352 (1,881 - 4,820) | 91.81 (51.52 - 132.02) | 1,641 (1,167 - 2,305) | 25.80 (18.34 - 36.23) | 0.08 (-0.29 - 0.79) | -3.59 (-3.90 to -3.29) |
| Serbia | 101 (70 - 142) | 4.64 (3.23 - 6.53) | 36 (24 - 52) | 2.70 (1.78 - 3.93) | 0.20 (-0.14 - 0.68) | -1.58 (-1.74 to -1.41) |
| Seychelles | 2 (1 - 3) | 7.63 (5.48 - 13.73) | 3 (2 - 4) | 12.50 (7.39 - 16.84) | 2.83 (0.55 - 4.55) | 2.85 (2.22 - 3.48) |
| Sierra Leone | 2,645 (1,086 - 3,979) | 145.95 (59.91 - 219.52) | 1,737 (962 - 2,553) | 48.57 (26.91 - 71.38) | -0.00 (-0.32 - 0.61) | -3.55 (-3.73 to -3.37) |
| Singapore | 103 (91 - 117) | 15.90 (14.00 - 18.05) | 95 (79 - 118) | 11.69 (9.73 - 14.52) | 2.22 (1.82 - 2.57) | -0.21 (-0.61 - 0.19) |
| Slovakia | 117 (93 - 149) | 8.85 (6.98 - 11.23) | 48 (36 - 65) | 5.63 (4.15 - 7.61) | -0.02 (-0.21 - 0.22) | -1.15 (-1.34 to -0.96) |
| Slovenia | 24 (18 - 32) | 5.75 (4.43 - 7.65) | 26 (17 - 37) | 8.17 (5.52 - 11.98) | 0.42 (0.10 - 0.78) | 1.27 (0.55 - 1.99) |
| Solomon Islands | 20 (12 - 30) | 12.56 (7.99 - 19.14) | 17 (12 - 29) | 6.66 (4.48 - 11.26) | 1.33 (0.72 - 2.29) | -2.13 (-2.37 to -1.88) |
| Somalia | 3,761 (1,931 - 6,323) | 96.55 (49.56 - 162.30) | 4,161 (2,237 - 6,606) | 40.28 (21.66 - 63.96) | 1.14 (0.47 - 2.16) | -2.21 (-2.61 to -1.80) |
| South Africa | 746 (508 - 1,008) | 5.48 (3.73 - 7.41) | 553 (426 - 755) | 3.64 (2.80 - 4.96) | 0.79 (0.40 - 1.46) | -1.18 (-1.38 to -0.98) |
| South Sudan | 3,283 (1,253 - 5,561) | 125.12 (47.73 - 211.91) | 4,293 (2,632 - 6,712) | 99.96 (61.27 - 156.27) | 0.69 (0.20 - 1.43) | -0.46 (-1.16 - 0.24) |
| Spain | 286 (241 - 346) | 3.65 (3.08 - 4.41) | 235 (195 - 293) | 3.63 (3.00 - 4.52) | 3.01 (2.40 - 3.56) | 0.17 (-0.21 - 0.55) |
| Sri Lanka | 691 (500 - 897) | 12.48 (9.04 - 16.22) | 353 (230 - 488) | 6.91 (4.50 - 9.57) | 1.51 (0.27 - 3.10) | -1.40 (-1.94 to -0.86) |
| Sudan | 3,042 (1,567 - 4,655) | 34.21 (17.62 - 52.35) | 2,743 (1,684 - 3,949) | 16.53 (10.15 - 23.80) | 0.69 (0.17 - 1.48) | -1.93 (-2.24 to -1.62) |
| Suriname | 39 (30 - 53) | 29.72 (23.15 - 40.31) | 48 (31 - 67) | 33.74 (21.73 - 46.53) | 2.80 (0.91 - 4.42) | 0.47 (0.18 - 0.77) |
| Sweden | 73 (62 - 87) | 4.74 (4.03 - 5.62) | 46 (33 - 63) | 2.52 (1.80 - 3.47) | -0.02 (-0.14 - 0.09) | -1.55 (-1.81 to -1.28) |
| Switzerland | 28 (22 - 36) | 2.41 (1.93 - 3.10) | 29 (22 - 38) | 2.16 (1.65 - 2.83) | 2.67 (2.19 - 3.14) | -0.04 (-0.19 - 0.12) |
| Syrian Arab Republic | 4,335 (2,916 - 5,754) | 73.20 (49.24 - 97.16) | 878 (664 - 1,226) | 23.96 (18.11 - 33.48) | 0.10 (-0.27 - 0.69) | -3.22 (-3.88 to -2.55) |
| Taiwan (Province of China) | 388 (356 - 420) | 7.04 (6.47 - 7.62) | 217 (190 - 250) | 7.37 (6.44 - 8.48) | 4.56 (3.84 - 5.22) | 1.22 (0.38 - 2.07) |
| Tajikistan | 1,540 (1,176 - 1,993) | 66.34 (50.66 - 85.85) | 1,460 (992 - 2,454) | 40.72 (27.66 - 68.46) | 0.63 (0.17 - 1.40) | -1.89 (-2.20 to -1.57) |
| Thailand | 2,910 (1,754 - 4,993) | 17.26 (10.40 - 29.61) | 1,615 (1,315 - 1,978) | 16.54 (13.47 - 20.25) | 1.69 (0.68 - 3.17) | 0.18 (-0.01 - 0.38) |
| Timor-Leste | 138 (61 - 199) | 41.60 (18.48 - 59.84) | 79 (58 - 104) | 15.17 (11.05 - 19.88) | 0.79 (0.35 - 1.47) | -3.57 (-3.88 to -3.26) |
| Togo | 1,153 (612 - 1,654) | 65.43 (34.74 - 93.87) | 811 (514 - 1,159) | 24.50 (15.53 - 35.02) | 0.51 (0.03 - 1.24) | -2.86 (-3.02 to -2.70) |
| Tokelau | 0 (0 - 0) | 6.24 (3.98 - 10.34) | 0 (0 - 0) | 19.30 (12.78 - 35.64) | 0.06 (-0.21 - 0.43) | -0.76 (-2.45 - 0.95) |
| Tonga | 5 (3 - 7) | 11.14 (7.58 - 16.17) | 3 (2 - 5) | 7.44 (4.70 - 12.39) | 0.49 (0.06 - 1.04) | -1.26 (-1.56 to -0.95) |
| Trinidad and Tobago | 36 (29 - 45) | 8.96 (7.24 - 10.96) | 39 (30 - 50) | 14.17 (11.03 - 18.32) | 3.74 (2.53 - 5.06) | 2.06 (1.25 - 2.87) |
| Tunisia | 557 (387 - 736) | 17.93 (12.45 - 23.71) | 185 (140 - 239) | 6.70 (5.07 - 8.63) | 1.23 (0.59 - 2.15) | -2.65 (-2.83 to -2.48) |
| Turkey | 4,419 (3,018 - 6,065) | 21.57 (14.73 - 29.60) | 1,174 (885 - 1,533) | 6.34 (4.78 - 8.28) | 0.74 (0.23 - 1.28) | -3.54 (-3.68 to -3.39) |
| Turkmenistan | 515 (449 - 595) | 34.31 (29.91 - 39.65) | 931 (768 - 1,123) | 61.06 (50.38 - 73.70) | 5.53 (4.06 - 7.57) | 2.10 (1.69 - 2.51) |
| Tuvalu | 1 (0 - 1) | 26.45 (13.75 - 40.38) | 0 (0 - 0) | 6.24 (4.09 - 9.28) | 0.18 (-0.06 - 0.47) | -4.42 (-4.69 to -4.15) |
| Uganda | 6,278 (3,356 - 9,982) | 74.57 (39.86 - 118.56) | 8,050 (4,989 - 12,072) | 40.58 (25.15 - 60.86) | 1.22 (0.62 - 2.25) | -1.58 (-1.80 to -1.35) |
| Ukraine | 2,028 (1,589 - 2,586) | 17.83 (13.97 - 22.74) | 801 (590 - 1,062) | 12.62 (9.30 - 16.74) | -0.06 (-0.28 - 0.21) | -0.75 (-1.41 to -0.08) |
| United Arab Emirates | 66 (49 - 89) | 11.13 (8.33 - 15.15) | 62 (45 - 84) | 4.61 (3.36 - 6.29) | 2.66 (1.23 - 4.27) | -1.89 (-2.20 to -1.57) |
| United Kingdom | 291 (249 - 350) | 2.67 (2.28 - 3.21) | 463 (406 - 530) | 3.93 (3.45 - 4.49) | 3.39 (3.02 - 3.70) | 1.84 (0.57 - 3.13) |
| United Republic of Tanzania | 14,653 (7,878 - 22,766) | 121.34 (65.24 - 188.53) | 13,168 (8,888 - 19,378) | 53.96 (36.42 - 79.41) | 0.63 (0.19 - 1.27) | -2.05 (-2.25 to -1.85) |
| United States of America | 3,908 (3,473 - 4,476) | 6.99 (6.21 - 8.01) | 2,996 (2,554 - 3,568) | 5.04 (4.30 - 6.00) | 0.92 (0.86 - 0.99) | -1.00 (-1.17 to -0.83) |
| United States Virgin Islands | 4 (3 - 5) | 12.33 (9.12 - 17.17) | 1 (1 - 1) | 7.34 (4.93 - 10.54) | 0.48 (0.05 - 1.01) | -1.18 (-1.47 to -0.89) |
| Uruguay | 45 (39 - 53) | 5.47 (4.71 - 6.52) | 62 (50 - 74) | 9.38 (7.63 - 11.28) | 8.52 (7.40 - 9.66) | 1.72 (1.01 - 2.43) |
| Uzbekistan | 1,870 (1,524 - 2,325) | 21.86 (17.82 - 27.18) | 1,852 (1,519 - 2,263) | 18.35 (15.05 - 22.42) | 0.90 (0.45 - 1.42) | 1.33 (0.32 - 2.35) |
| Vanuatu | 6 (4 - 10) | 9.31 (6.14 - 14.03) | 7 (5 - 10) | 5.76 (4.03 - 8.36) | 1.53 (0.90 - 2.43) | -1.58 (-2.04 to -1.13) |
| Venezuela (Bolivarian Republic of) | 1,159 (1,004 - 1,375) | 16.34 (14.15 - 19.39) | 1,234 (950 - 1,610) | 18.63 (14.34 - 24.30) | 4.24 (3.08 - 5.71) | 1.12 (0.69 - 1.56) |
| Viet Nam | 503 (363 - 791) | 1.90 (1.37 - 2.98) | 351 (261 - 474) | 1.42 (1.06 - 1.92) | 1.15 (0.40 - 2.09) | -0.69 (-0.79 to -0.58) |
| Yemen | 1,547 (870 - 2,363) | 21.81 (12.27 - 33.31) | 1,699 (1,017 - 2,428) | 12.32 (7.37 - 17.61) | 1.14 (0.56 - 2.10) | -1.58 (-1.88 to -1.29) |
| Zambia | 3,685 (1,858 - 5,568) | 98.16 (49.49 - 148.31) | 2,700 (1,764 - 4,326) | 32.65 (21.32 - 52.29) | 1.18 (0.23 - 3.54) | -3.27 (-3.44 to -3.11) |
| Zimbabwe | 177 (105 - 243) | 3.67 (2.17 - 5.04) | 353 (192 - 525) | 5.60 (3.05 - 8.35) | 1.98 (0.94 - 3.58) | 2.20 (1.84 - 2.56) |
